# Biodegradability of polyhydroxyalkanoate (PHA) biopolyesters in nature: a review

**DOI:** 10.1007/s10532-025-10164-y

**Published:** 2025-08-11

**Authors:** Martin Koller, Dustin Heeney, Anindya Mukherjee

**Affiliations:** 1 Institute of Chemistry, University of Graz, NAWI Graz, Heinrichstrasse 28/IV, 8010 Graz, Austria; 2 PHAXTEC, Inc., 2 Davis Drive, Research Triangle Park, Durham, NC 27709 USA; 3 GO!PHA, 12324 Hampton Way, Wake Forest, NC 27587 USA

**Keywords:** Biodegradation, Biopolymers, Sustainability, Circular economy, Composting, Natural polymers, Polyhydroxyalkanoates, Alternatives and substitutes

## Abstract

In the search for sustainable alternatives and substitutes to overcome plastic pollution, polyhydroxyalkanoates (PHA) stand out as the gold standard. The very fact that PHA are microbially produced from renewable carbon sources, biodegraded by microbial action, and possess the beneficial properties of over 50% of the world’s plastics has caught the attention of a wide range of producers, converters, brand owners, and policy makers with a view to replace conventional fossil-based plastics with these natural materials. PHA are readily biodegraded by the enzymatic toolbox of living organisms, aligning with the principle of natural circularity. Over 150 different monomeric building blocks of PHA have been identified, leading to a wide variety of naturally accessible PHA biopolyesters with diverse properties that include thermoplastic and crosslinkable polymers for single use and durable uses for packaging and personal care and as paints, coatings and adhesives, and as fibers for fabrics and textiles. The type of monomer and microstructure, as well as the environment, play important roles in their production and biodegradation. This comprehensive paper reviews the degradability of commercially available and other PHA types with varying microstructures in fresh water, sea water, soil, as well as in home and industrial composting and anaerobic conditions. Unlike previous reviews the authors integrate information from diverse biodegradation studies and provide a holistic view and understanding of the biodegradability of the PHA biopolymer family in nature and in industrial environments.

## Introduction and the basics of polyhydroxyalkanoates (PHA)

The concept and practice of Circular Economy, and especially Circular Bioeconomy, is experiencing a dynamic upswing around the world. In this context, the scope of bio-based and biodegradable polymers like PHA is expanding due to their intrinsic sustainability and circularity and the opportunity to overcome prevailing global issues like depletion of resources and plastic pollution; PHA are intrinsically circular materials and are already contributing to sustainable development and economic growth (Saranya et al. 2020; Mukherjee and Koller 2022a).

PHA are naturally produced and degradable polymers (biopolymers), similar to polysaccharides (starch, cellulose, hemicellulose, chitin, etc.), polyamides (proteins), nucleic acids, lipids, cutin, suberin, or polyphenols such as lignin. Being biobased, biosynthesized, biodegradable, and biocompatible, PHA stand out among the vast array of diverse bio-attributed polymers (“biopolymers”) and are being industrially produced with thermoplastic properties. Indeed PHA production is an emerging field of industrial biotechnology (“white biotechnology”), which makes these biopolymers the go-to class of materials in finding solutions for the ever increasing plastics pollution crisis (Wei et al. 2020; Mukherjee and Koller 2022b). More specifically, it is exclusively microbes which, on the one hand, biosynthesize PHA, and, on the other hand, biocatalyze biodegradation; hence, it is the world of microbes, which makes PHA sustainable and circular (Choi et al. 2023).

In fact, PHA are the only group of intrinsically natural so called “bioplastics” with thermoplastic as well as thermoset characteristics that makes PHA industrially significant (Mukherjee and Koller 2023). The global market for “bioplastics” is estimated at US$ 17.54 B in 2023, and is currently experiencing a significant growth in demand, including microbial PHA biopolyesters. From 2024 to 2030, the market for all “bioplastics” is expected to increase at a Compound Annual Growth Rate (CAGR) of 10.4%. This growing demand is primarily driven by the increasing need for ecologically benign packaging and personal care materials (Lackner et al. 2023).

Originally found as large intracellular granules in 1888 by MW Beijerinck in Bacillus-shaped bacterial cells and identified as PHA in 1926 by Maurice Lemoigne, they were later confirmed to function as carbon and energy storage molecules in microorganisms under unfavorable environmental conditions. Further investigations detailed PHA inclusions´ crucial role in protecting cells against stress factors, such as osmotic imbalance, UV radiation, and heat shock (Obruca et al. 2021). The simplest form of PHA, poly(3-hydroxybutyrate) or P(3HB), is ubiquitous and is the best described type. Recently comprehensively reviewed by Seebach, short chain P(3HB) biopolyesters composed of less than 150 monomeric units have been found to occur in all life forms, including humans, where they act as cellular components, e.g., ion channels. This review emphasized that there is literally “No Life on this Planet” without P(3HB) (Seebach 2023).

Chemically, PHA constitute linear polyesters composed of hydroxycarboxylic acids, predominantly 3-hydroxyalkanoates, while a restricted number of PHA isolated from natural samples also contain 4-, 5-, and 6-hydroxyalkanoates (Rehakova et al. 2023). Unsaturated hydroxyalkenoates are also polymerized into PHA by some strains (Gao et al. 2022). In many cases, the hydroxyl group is not located on the terminal carbon atoms, which makes most PHA monomers chiral molecules. Importantly, all natural chiral PHA building blocks are *R*-configured due to the specificity of the enzymes catalyzing polymerization, namely PHA synthases, which possess active sites exclusively recognizing and incorporating *R*-configured substrates. This stereospecificity guarantees the accurate orientation of monomers during the polymerization process, resulting in the generation of structurally regular and biologically functional polyesters (Agnew and Pfleger 2013). After the life span of the PHA, chiral monomers can be chemically recycled and used as synthons for synthesis of marketable chiral chemicals (Chen and Wu 2005).

PHA are classified based on the number of carbon atoms present in the monomeric building blocks; short chain length PHA or *scl*-PHA monomers contain 3 to 5 carbon atoms, medium chain length PHA or *mcl*-PHA monomers contain 6–14 carbon atoms, and the not as well studied group of long chain length PHA or *lcl*-PHA monomers comprise 15 and more carbon atoms. Based on the material properties of these groups, *scl*-PHA stand out as typical thermoplastics with low elongation at break and high tensile strength and crystallinity with the exception of high fraction of 4-hydroxybutyrate—4HB monomer containing *scl*-PHA being of non-chiral nature. *Mcl*-PHA and *lcl*-PHA have attracted attention as flexible elastomers and sticky resins with low to no crystallinity and having melting points not too far above room temperature, typically far below the boiling point of water, and remarkably low glass transition temperature far below the freezing point of water (see Table 1). To address the vast variety of different types of PHA, termini like the “PHAome” (Chen and Hajnal 2015) or the “PHAmily” (Lackner et al. 2024) have been introduced by scientists active in this field.

**Table 1:** Major physical and mechanical properties of the most important industrialized types of PHA

PHA	Group	Type	Degree of crystallinity *X*_*c*_ [%]	Melting point *T*_*m*_ [°C]	Glass transition temperature *T*_*g*_[°C]	Elongation at break [%]	Tensile strength [MPa]	Tensile modulus (Young´s modulus) [MPa]	Production strains typically used in industry	Selected Refs.
P(3HB)	Homopolyester	*scl*-PHA	60–80; 55–62	170–180	4.8–5.0	2–5	25–50	700–900; 1765	*C. necator*	Zhila and Shishatskaya (2018); Sudesh et al. (2000); Das et al. (2018); Thellen et al*.* (2018); Doi et al. (2019); Fiorese et al. (2009)
P(4HB)	Homopolyester	*scl*-PHA	57	60	− 51	1000	50	70	Rec. *Escherichia coli,* 3HB-leaky mutants of *C. necator* JMP222	Martin and Williams (2003); Steinbüchel et al. (1994)
P(3HB-*co*-3HV)	Heteropolyester (Copolyester)	*scl*-PHA	30–69; 7–34. 35–60% for 10 and 20 mol-% 3HV	102–165	− 10–20	8–12; 1.26–4.49	21–36; 152–22.1	900–1500	*C. necator*	Zhila and Shishatskaya (2018); Das et al. (2018); Thellen et al. (2018); Doi et al. (2019); Verhoogt et al. (1994); Modi et al. (2011); Luzier (1992)
P(3HB-*co*-4HB)	Heteropolyester (Copolyester)	*scl*-PHA	30–53	150–162; 52–143	Amorphous at high 4HB fraction; about − 34 to 3 at low fractions (below 30%)	5.7–365.4	7.3–15.4	34–425	*C. necator*	Zhila and Shishatskaya (2018); Doi et al. (2019); Ishida et al. (2001)
P(3HB-*co*-3HV-*co*-4HB)	Heteropolyester (Terpolyester)	*scl*-PHA	26–35	164–166	− 5	47.6–365.4	5.3–8.8	34.3–130.5	*C. necator*	Zhila and Shishatskaya (2018)
P(3HB-*co*-3HV-*co*-4HB-*co*-3HHx)	Heteropolyester (Quarterpolyester)	*scl-mc*l-PHA	39–45	161–169	− 4.4 to − 0.7	49.0–93.7	7.3–11.7	127.8–419.4	*C. necator*	Zhila and Shishatskaya (2018)
P(3HB-*co*-3HHx)	Heteropolyester (Copolyester)	*scl*-*co*-*mcl*-PHA	27.2 (11% 3HHx)	115–138 106.3 (11–13% 3HHx,)	1.2 (11% 3HHx) − 0.21 (11–13% 3HHx,)	12 (11% 3HHx) 11.5 (11–13% 3HHx)	11 (11% 3HHx) 6 (11–13% 3HHx)	17 (11% 3HHx) 267 (11–13% 3HHx)	*Aeromonas* sp., rec. *C. necator*	Zeng et al. (2022); Hassan et al. (2006)
Different types of mcl-PHA	Heteropolyester (Co-, ter-, or quarterpolyester)	*mcl*-PHA	20–40 (for P(3HHx-*co*-3HO)	Ca. 40–70 (highly dependent on composition)	Ca. − 50 to − 30	100–300 (highly dependent on composition)	2–9 (highly dependent on composition)	4–34 (highly dependent on composition)	*Pseudomonas putida*	Zinn (2010); Larrañaga and Lizundia (2019)
P(3HO)	Homopolyester	*mcl*-PHA	37.5	39–52	− 36 to − 34	204	1.8	1.5–11.4	*Pseudomonas mendocina*	Rai et al. (2011); Basnett et al. (2012)

**Fig. 1:**
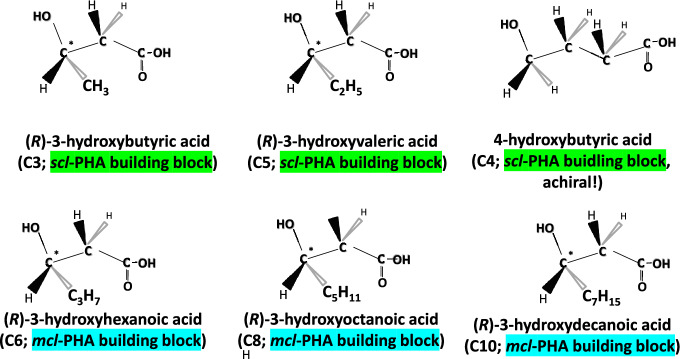
Industrially important *scl*- and *mcl*-PHA building blocks

The class of PHA synthase present in a microbial PHA production strain determines the group of PHA to be produced. While Class I, III, and IV synthases specifically catalyze the polymerization of *scl*-PHA building blocks, Class II synthases are responsible for biosynthesis of *mcl*- and *lcl*-PHA (Tsuge et al. 2015). A noteworthy exception is the randomly built-up hybrid *scl*-*mcl*-PHA poly(3-hydroxybutyrate-*co*-3-hydroxyhexanoate) [P(3HB-*co*-3HHx)], a tough and flexible material currently witnessing rapidly increasing industrial production, especially for manufacturing marine biodegradable single-use foodservice items like teabags, coffee pods, drinking straws, plates and cutlery. Biosynthesis of this intriguing copolyester by members of the *Aeromonas* genus (e.g., *Aeromonas caviae* or *Aeromonas hydrophila*) is enabled by presence of an atypical Class I PHA synthase, which is able to polymerize not only monomers with 4 and 5 carbons (*scl*-PHA monomers), but also 3-hydroxyalkanoate (3HHx), a typical *mcl*-PHA building block (Ushimaru et al. 2014). Industrial endeavors to produce P(3HB-*co*-3HHx) on a larger scale are also based on recombinant *Cupriavidus necator* strains (Tan et al. 2021). Figure 1 presents best studied monomers making up different types of PHA.

**Fig. 2:**
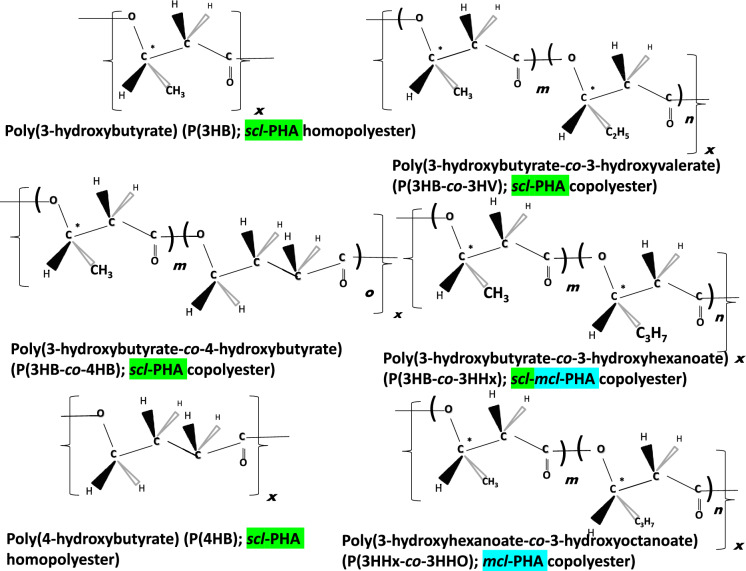
Industrially relevant and commercial PHA homo- and co-, ter and quarterpolyesters where **x = **degree of polymerization; m = molar fraction of 3HB; **n = **molar fraction of 3HV; **o = **molar fraction of 4HB; **p = **molar fraction of 3HHx, and **q = **molar fraction of 3HO

PHA are categorized into homo- and heteropolyesters based on their monomeric composition. Homopolyesters contain only one type of monomer; poly(3-hydroxybutyrate) [P(3HB)] and poly(4-hydroxybutyrate) [P(4HB)] are of industrial significance. Narancic et al. (2020) has shown that specific physicochemical, biological, and biodegradation properties of PHA biopolymers make them appealing for biomedical applications, such as tissue engineering, drug delivery systems including as tailored nanoparticles. P(3HB) possesses thermoplastic properties with high crystallinity and brittleness (Hänggi 1990), and P(4HB) exhibits very high elongation at break (~ 1000%) and low tensile modulus behaving like an elastomer with biomedical uses, such as for sutures and surgical wires. P(4HB) was the first PHA with an FDA approval for use in medical devices (Mitra et al. 2021). Among heteropolymers (two or more types of monomers present in the polyester chain), co-, ter-, and quarterpolyesters are industrially relevant (Koller and Mukherjee 2022). Specifically, copolyesters of 3-hydroxybutyrate (3HB) with 3-hydroxyvalerate (3HV), 4-hydroxybutyrate (4HB), and 3-hydroxyhexanoate (3HHx): namely poly(3-hydroxybutyrate-*co*-3-hydroxyvalerate) [P(3HB-*co*-3HV)], poly(3-hydroxybutyrate-*co*-4-hydroxybutyrate) [P(3HB-*co*-4HB)], and (3HB-*co*-3HHx) respectively, are already being industrially produced (Koller and Mukherjee 2022). These three copolyesters exhibit lower crystallinity than the homopolyester P(3HB), making them easier to process and more prone to biodegradation in natural environments (Kourmentza et al. 2017). Figure 2 illustrates the most important representative PHA homo- and heteropolyesters. *Mcl*-PHA is represented by P(3HHx-*co*-3HO).

Table 1 summarizes important physical and mechanical properties of the industrially relevant PHA along with the most important microbial production strains used to produce them industrial scale.

The major characteristics of these PHA biopolyesters, from a functional, and chemical property standpoint are as follows:

- *a) Biosynthesis*: Microbes use heterotrophic renewable carbon resources found in nature, such as carbohydrates, lipids, or alcohols like glycerol to biosynthesize PHA. Cyanobacteria (Price et al. 2022) and hydrogen-oxidizing “Knallgas” bacteria (Lambauer et al. 2023) synthesize photoautotrophic and chemolithotrophic PHA based on CO_2_ utilization, respectively. Several type-II methanotrophs utilize CH_4_ as the sole carbon source for PHA biosynthesis (Rodríguez et al. 2020).
- The most studied metabolic pathway generating P(3HB) from substrates like carbohydrates follows catabolic breakdown of the substrate to the central metabolite acetyl-CoA, which, under nutritionally imbalanced conditions (excess of carbon source, limitation of other growth-essential nutrients like nitrogen- or phosphate source), deviates from the citric acid cycle towards P(3HB) biosynthesis. The first step in P(3HB) biosynthesis is the condensation of two acetyl-CoA molecules by the 3-ketothiolase enzyme (phaA), generating acetoacetyl-CoA. This, in turn, is reduced by acetoacetyl-CoA reductase (phaB) to (*R*)-3-hydroxy-butyryl-CoA, the substrate of PHA synthase (phaC), which then carries out the polymerization reaction to generate P(3HB). Genes encoding these key enzymes are typically grouped together on the *phaCAB* gene cluster (Braunegg et al. 1998).
- *Scl*-PHA copolyester production requires structurally related carbon sources acting as precursors for desired building blocks during cultivation, in addition to the main carbon source (e.g., sugar), which yields 3HB building block. Such precursor compounds constitute propionic acid, valeric acid, and levulinic acid (structurally related to 3-hydroxyvalerate, 3HV), or *γ*-butyrolactone, 1,4-butandiol, and 4-hydroxybutyrate (structurally related to 4HB) (Braunegg et al. 1998). Alternatively, genetic engineering efforts have also created *scl-*PHA copolyesters in several industrially relevant microbial species such as *Cupriavidus necator* (Nangle et al. 2020; Tanaka et al. 2021)*, Escherichia coli* (Wang et al. 2014)*, Methylosinus* sp. (Nguyen and Lee 2021), and *Halomonas* sp. (Ye et al. 2018) without the need to feed precursor compounds. These GMO strains mostly produced P(3HB-*co*-4HB) with varying degrees of 4HB molar ratios and P(3HB-*co*-3HHx) copolyesters using recombinant organisms (Tang et al. 2022). For example *C. necator* harboring the *Chromobacterium* sp. PHA synthase gene (Anis et al. 2012), or the recombinant *C. necator* strain Re2058/pCB113 was utilized to produce P(3HB-*co*-3HHx) copolyester on crude palm kernel oil as the sole carbon source (Murugan et al. 2016).
- *b) Biodegradability*: An outstanding property of PHA biopolyesters is their biodegradability, which constitutes the core of the present review. PHA are degraded by microbes (bacteria, fungi) synthesizing PHA depolymerase enzymes (phaZ) into natural end-products, namely CO_2_ and water under aerobic conditions; under anaerobic conditions, such as those prevailing in biogas and anerobic digestion plants, CH_4_ is also generated. Besides classical PHA depolymerases, more recent studies demonstrate PHA degradation through the actions of unspecific enzymes, such as triacylglycerol lipase-like enzymes (de Vogel et al. 2021). Importantly, the end-products of aerobic and anerobic biodegradation can be reused for PHA biosynthesis illustrating the circularity of PHA biosynthesis and breakdown, being perfectly embedded into Nature´s closed material cycles. The biodegradability of PHA matches the 12 Principles of Green Chemistry: Principle 10 postulating that “chemical products should be designed so that at the end of their function they break down into innocuous degradation products and do not persist in the environment.” (Ivanković et al. 2017).
- The first reported study on microbial PHA biodegradation was elucidated by Lepidi et al. in the early 1970s*.* Lepidi et al. fed micro-fungi with radio-labeled P(3HB) and demonstrated the organisms accumulating radioactive compounds originating from the radio-labeled P(3HB) substrate, thus unambiguously confirming for the first time the degradability of P(3HB) by the action of living organisms (Lepidi et al. 1972).
- At this juncture the authors would like to mention a few words on the “conventional plastic biodegradation”. The area is of immense interest and significance given the amount of such plastics of petrochemical origin that are produced and discarded. These studies are being typically carried out under conditions that do not prevail in nature, such as, excessive concentrations of hydrolytic enzymes when studying “bio”-degradation of the polyester poly(ethylene terephthalate) (PET) (Koller et al. 2023). Reports on “biodegradation” of petrochemical polyolefins (polyethene and related hydrocarbon plastics) are often contradictory, misleading, and erroneous, as has recently been comprehensively and critically reviewed by Jendrossek (2024). Indeed, current reasonable approaches to address the plastic crisis using biotechnology are mostly restricted to biopolyesters, hence, PHA (Wei et al. 2020).
- *c) Thermoplasticity*: PHA possess thermoplastic characteristics, they can be melted and formed into various shapes when heated; upon cooling, they solidify again. This thermoplasticity renders them suitable for various processing techniques in existing conventional plastics equipments, such as injection molding (Cinelli et al. 2019), melt extrusion (Thellen et al. 2008), and extrusion film blowing (Teixeira et al. 2020; Cinelli et al. 2019). Additive manufacturing (3D printing) (Kovalcik 2021), electrospinning (Brunetti et al. 2020), or compression molding (Rastogi and Samyn 2020) are further processing techniques successfully applied with PHA.
- *d) Solvent and water solubility*: Increasingly, PHA are applied as solvent-borne coating materials (Rodríguez-Contreras et al. 2017). Hence, PHA are functional like fossil-based plastics, but PHA biopolymers do not create persistent microplastics and do not accumulate in the environment. Natural PHA are typically water-insoluble, but are soluble in various organic solvents. This solubility depends on the type and arrangement of monomers in the PHA chain and enables the preparation of PHA specimens via solvent casting: PHA is dissolved in adequate solvents; from this solution, the solvent evaporates, leaving behind thin PHA films of custom-made thickness, which can be used for biodegradation studies. Due to their hydrophobicity, PHA biopolyesters prepared as dispersions in water have also been extensively studied for their potential use as paper coating materials (Samrot et al. 2021). Coating PHA biopolyesters on paper equips it with novel properties, such as increased water-repellency, while maintaining paper’s environmentally beneficial characteristics such as the recycling and composting capacity (Basak et al. 2024). In addition, PHA coating can functionalize the coated material, e.g., antimicrobial effects, as previously shown in the case of titanium implants dip-coated with gentamicin-loaded PHA (Rodríguez-Contreras et al. 2019).
- *e) Variability in monomer composition*: As has been mentioned earlier, physical and mechanical properties of PHA biopolymers can be tuned by varying the monomeric composition during their biosynthesis. Different hydroxycarboxylic acids of varying chain lengths and functional groups incorporated into the PHA polyester chain generates PHA with varying elongation at break, tensile and flexural modulus, and impact properties. The monomers that are incorporated into PHA chains *in statu nascendi* depend on both, the carbon source used (specific precursor compounds generate structurally related PHA monomers) and the PHA synthase present in the microbial production strain. While many strains exclusively produce *scl*-PHA, like the best described PHA producer *Cupriavidus necator*, others like *Pseudomonas* sp. are specific for *mcl*-PHA production (Braunegg et al. 1998). Besides the types of monomers, their distribution within the biopolyester imparts material properties as well. For example, PHA heteropolymers having a random distribution of the monomers is distinct from blocky structured PHA (*b*-PHA), where blocks of one monomer alternate with blocks of other monomers, resulting in PHA polyesters that consist of different monomer segments (“soft” and “hard” segments) and this results in material properties that are different from random copolyesters despite both having the same overall monomer content. This difference results in the random copolymer having different physical and mechanical properties compared to the block copolymer (Mai et al. 2023). McChalicher and Srienc has demonstrated that films with blocky structured copolymers [segments of PHB and random P(3HB-*co*-3HV)] maintained higher elasticity over time in comparison to films of random copolymers of similar comonomer composition (McChalicher and Srienc 2007).
- *f) Inertness*: PHA polymers have good durability and can be made into many commodity items with long shelf life. PHA are chemically inert to most household liquids materials and will not breakdown generally when used daily. A toothbrush, cup or a spoon made of PHA will not disintegrate in the bathroom or the cutlery drawer, respectively; only when subjected to a microbial enzyme catalyzing disintegration, such as encountered at the compost heap, or long-term exposure in the soil, freshwater or marine environments. This was confirmed, when Mergaert et al. (1993) noticed biodegradation of P(3HB) and P(3HB-*co*-3HV) in soil rich in microorganisms, but not in sterile buffer solution, and by Luzier (1992), who demonstrated the stability of ICI-BIOPOL™ shampoo bottles made of P(3HB-*co*-3HV) in humid air, but 100% mass loss after 6, 40, 60, 75, and 350 weeks in anaerobic sludge, estuarine sediment, aerobic sewage, soil, and marine water, respectively. Moreover, PHA are water-insoluble due to their hydrophobic nature; this enables their existence as inert cytoplasmic inclusion bodies (“carbonosomes”, “intracellular PHA granules”) in microbial cells (Jendrossek 2020). This hydrophobicity of PHA makes it also interesting for packaging of food, where high oxygen and water vapor barrier are required (Koller 2014). Importantly, these barrier properties also depend on the PHA composition; using the ASTM Standards F1249-99 (*Standard Test Method for Water Vapor Transmission Rate Through Plastic Film and Sheeting Using a Modulated Infrared Sensor*) and D3985-99 (*Oxygen Gas Transmission Rate Through Plastic Film and Sheeting Using A Coulometric Sensor*), respectively, Thellen et al. (2008) showed that melt extruded films of P(3HB-*co*-3HV) reveal higher barrier properties to oxygen and water vapor in comparison to the P(3HB) film specimens. For specific applications, however, additional surface modifications or copolymerization with hydrophilic monomers can be applied to reduce PHA hydrophobicity. In addition, most PHA biopolymers exhibit high UV stability protecting the PHA-rich microbial cells against excessive UV radiation (Slaninova et al. 2018), and this property of PHA has already been demonstrated through the use of PHA granules in UV-protective cosmetics (Liu et al. 2024).
- *g) Mechanical properties*: The mechanical properties of PHA, such as toughness, tensile strength, elasticity, or elongation at break, are strongly dependent on monomers present in the PHA and molecular mass (see Table 1). These properties mimic those of conventional plastics, making them the materials of choice for various applications, where fossil plastics can easily be replaced. Importantly, PHA can be processed in existing plastics processing machines, which facilitates the integration of PHA as a commercial material in existing manufacturing plants (Bugnicourt et al. 2014).

## Biodegradability in nature

In general, the term “biodegradation” describes the natural disintegration of organic materials by the action of prokaryotic (e.g., bacteria) or eukaryotic (e.g., fungi) organisms or by the isolated biocatalytic parts thereof (enzymes) (Lucas et al. 2008). Jendrossek (2024) defined “biodegradation” as simply the conversion of organic compounds into H_2_O and CO_2_. However, these definitions do not provide sufficient information on the environmental conditions (temperature, microbial community, pH-value, humidity, oxygen and nutrients availability, etc.), timescale and degree to which the decomposition process occurs (Harrison et al. 2018). Although frequently used synonymously, biodegradation and composting are not identical processes: While biodegradation is the overarching term for degradation of polymers in nature, composting calls for specific conditions, such as those found at homes and carried out at ambient temperatures, or during industrial compositing under elevated temperatures that use specific microorganisms. Importantly, “compostable” refers to the capability of organic materials to biodegrade and turn into compost, also referred to as organic fertilizer; “biodegradation” *per se* does not necessarily generate compost (Lavagnolo et al. 2020). Hence, each biodegradation environment able to biodegrade materials (soil, fresh water, sea water, compost) has its own requirements (Mohee and Unmar 2007). Standard EN 13432 is considered to be the “gold standard” for setting the requirements in categorizing a material as “biodegradable” [EN 13432:2001. *Requirements for packaging recoverable through composting and biodegradation- Test scheme and evaluation criteria for the final acceptance of packaging*]. It provides the following definitions for various types of biodegradation and composting for packaging materials:

- Soil biodegradation: 90% or more of the material degraded (carbon content metabolized, e.g., converted to CO_2_) at 20–25 °C in less than 24 months
- Fresh water biodegradation: 90% or more of the material degraded at 20–25 °C in less than 56 days
- Marine biodegradation: 90% or more of the material degraded at 20–25 °C in less than 6 months, and in addition, 10% or less of the remnants having a particle size above 2 mm after 84 days
- Home composting: 90% or more of the material degraded at 20–30 °C within less than 12 months; in addition, 10% or less of the remnants having a particle size above 2 mm after 6 months
- Industrial composting: 90% or more of the material degraded at 50–70 °C in less than 6 months; in addition, 10% or less of the remnants having a particle size above 2 mm after 12 weeks

All five criteria need to be fulfilled to categorize a polymer as “biodegradable”. Of note, commercial polymers, which do not correspond to these definitions and/or are labeled “green plastic”, are a direct result of organizations’ or individuals’ artful use of “greenwashing”, a term coined in the 1980s by Jay Westervelt to denounce dubious marketing of products and questionable practices to make products or materials appear ecologically benign and sustainable (Lackner et al. 2023).

Importantly, commercial PHA fulfills all the above-mentioned biodegradation criteria, as evidenced by the individual certifications for PHA produced by relevant companies.

Mechanistically, biodegradation processes proceed in four phases (Lucas et al. 2008):

- *Biodeterioration phase*: “Biodeterioration” describes the mechanical breakdown of the material structure. It can be understood as a surface degradation that alters the mechanical, physical and chemical material properties. Biodeterioration takes place when a material is exposed to abiotic environmental factors, such as pressure, abrasion, (UV) light, temperature, humidity, high or low pH, or chemicals. When studying PHA biodegradation, this biodeterioration typically involves surface roughening, formation of holes and cracks, and color change (Arcos-Hernandez et al. 2012). Biodeterioration is the initial step to prepare the polymer for microbial attack in the subsequent “Biofragmentation phase”.
- *Biofragmentation phase*: “Biofragmentation” or “cleavage/depolymerization” is the biotic (enzymatic) or abiotic breakdown of a material. For polymers, it is a hydrolytic process, cleaving bonds in polymer chains, resulting in the generation of oligo- and monomers (hydroxycarboxylic acids in the case of PHA biodegradation). During biofragmentation of PHA, established several decades ago, microorganisms colonize the surface of the polymer and excrete extracellular depolymerase enzymes (PhaZ). The enzymes degrade the material into the building blocks, e.g., 3HB or 3HV (Ishida et al. 2001). Importantly, biodeterioration and depolymerization are considered rate determining steps in PHA biodegradation (Arcos-Hernandez et al. 2012).
- *Bioassimilation phase*: “Bioassimilation” refers to the uptake of the products of biofragmentation or the building blocks (mono- and oligomeric hydroxy acids in the case of PHA) by living cells, either via easy transport mechanisms supported by membrane carriers, or after biotransformation steps (Meereboer et al. 2020).
- *Mineralization phase*: Inside the cells, bioassimilated products undergo catabolic reactions to generate cell structure elements, energy, and the ultimate products of the final mineralization released by cells. In the case of PHA, besides cell mass, energy and excreted small molecules (e.g., small carboxylic acids), CO_2_, water and humus are also produced as a result of biodegradation under aerobic conditions, while during anaerobic breakdown of PHA CH_4_ is also released (Luzier 1992).

**Fig. 3:**
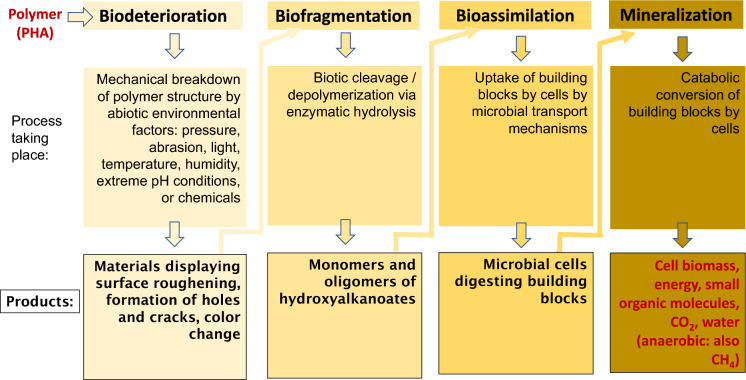
Schematic of the four phases of biodegradation: Biodeterioration, Biofragmentation, Bioassimilation, Mineralization

Figure 3 provides a schematic of the four phases of biodegradation.

In theory, all chemical materials, be it biopolymers or xenobiotics (e.g., fossil plastics) can undergo biodegradation at least to some extent. Lavagnolo et al*.* remarked: “*Without any timescale specification, all materials are therefore inherently biodegradable, whether it takes a few weeks or a million years to break down into water, carbon dioxide and methane*.” (Lavagnolo et al. 2020) Polymers classified as “biodegradable” based on standardized test methods, norms and certificates need to disintegrate in each environment within a strictly defined time frame (as is the case for fruits, vegetables and other organic products undergoing disposal as “bio-waste”) and as described above according to the guidelines set by the standard EN 13432. Other products such as glass and especially the top-selling fossil plastics, despite expected disintegration into tiny fragments (e.g., micro- and nanoplastic particles), will not (bio)degrade within a span of several human generations or even millennia. Diverse environmental and material-specific factors determine the biodegradation rates of materials:

- *a) Composition of a material*: No serious scientific article unambiguously reports on the ultimate biodegradation of fossil-based plastics such as polyolefins like poly(ethene) (PE), poly(propene) (PP), poly(vinyl chloride) (PVC), or poly(styrene) (PS) in natural environments, although studies postulate a certain mass loss of PS (Jiang et al. 2021) or PE (Poma et al. 2022) when fed to wax moth larvae. However, results presented in these studies only substantiate biofragmentation of these polymers, and do not confirm biodegradation of the full-carbon-backbones material and final mineralization, as required by established standards and norms (Koller et al. 2023). In contrast to polyolefins, polyesters such as PET are prone to biodegradation *sensu stricto* by specific esterases (“PETases”) due to the presence of ester bonds linking the PET monomers (terephtalic acid and ethylene glycol). However, presence of these specific esterases is limited to a handful of discovered microorganisms, and degradation rates achieved to date (under optimized laboratory conditions, in the presence of excess isolated enzymes) are far too slow to offer a practical solution in reducing or reusing PET waste. As has already been reported, PET is biodegraded by these wild type microorganisms extremely slowly and only when the ester backbone can become accessible through other abiotic mechanisms (Urbanek et al. 2021). This contrasts with natural PHA polyesters, which constitute carbon- and energy sources for an innumerable number of natural organisms. Moreover, PET’s most touted property, namely its excellent mechanical recyclability, leads to the release of excessive amounts of microplastics with each recycling cycle (Guo et al. 2022). In contrast, the composition and microstructure of PHA biopolyesters was fine-tuned by Nature in a way to make them biodegradable by Nature’s biocatalytic toolbox; thus, upon biodegradation no PHA microparticles (MP) are left in the environment. This was confirmed by Cheng et al. who demonstrated biodegradability of spherical microbeads consisting of P(3HB-*co*-3HV) similar to those microplastics found in conventional cosmetics and personal care products in marine environments (17.0 ± 6.1% mass loss during 60 days of incubation). In contrast, microbeads consisting of PE and even poly (lactic acid) (PLA) were not degraded at all under the same conditions (Cheng et al. 2022). A recent study by Zha and colleagues applied a multi-omics approach to study the effect of airborne biodegradable PHA MP and non-biodegradable PP MP on lung and liver in mouse models. It was shown that PHA MP induce less pulmonary and hepatic toxicity compared to PP MP, suggesting PHA is a potential, less hazardous substitution for PP. In this exploratory and short study (42 days of exposure to MP once every 6 days), synthesized PHA MP exerted less inflammatory effects to mice than did PP MP (Zha et al. 2025).
- For different types of PHA, the composition and the chain length of the monomers strongly impact biodegradation rate. This was demonstrated well by Li et al. who tested enzymatic degradation of P(3HB), P(3HB-*co*-19-mol-%-3HV), P(3HB-*co*-19-mol-%-3HHx), and P(3HB-*co*-9-mol-%-3HO) in solutions of *Ralstonia pickettii* T1 PHA depolymerase for 25 h. Although crystallinity decreased in the order of P(3HB) > P(3HB-*co*-19-mol-%-3HV) > P(3HB-*co*-19-mol-%-3HHx) > P(3HB-*co*-9-mol-%-3HO), the degradation rate decreased in the following order P(3HB-*co*-19-mol-%-3HV) > P(3HB) > P(3HB-*co*-19-mol-%-3HHx) > P(3HB-*co*-9-mol-%-3HO). Hence, despite being less crystalline, PHA containing long side chains [here especially P(3HB-*co*-9-mol-%-3HO)] are less prone to enzymatic attack because the geometric structure impedes the access of the depolymerases to the ester bonds that make up the polyester backbone chains (Li et al. 2007).
- *b) Crystallinity*: Crystallinity of PHA is strongly impacted by the monomeric composition (Volova et al. 2017; Zhila and Shishatskaya 2018). PHA homopolyester P(3HB) with high crystallinity typically shows slower degradability than PHA copolyesters with low crystallinity, provided that the present monomers have similar side chain lengths, such as 3HB, 3HV, and 4HB (compare above-discussed study by Li et al. 2007). In this case, building blocks like 3HV and 4HB interrupt the structure of the typically highly crystalline P(3HB) bulk matrix, but are not too bulky in their structure, like 3HO or 3HD, which would prevent enzymatic attack of the ester bonds. This was only recently substantiated by Vodicka and colleagues, who studied degradation of P(3HB) and P(3HB-*co*-4HB) copolyesters in simulated body fluids. All PHA samples tested in this study were produced by the recently discovered strain *Aneurinibacillus* sp. H1 and were processed to thin solvent-cast films of about 11 µm thickness. P(3HB-*co*-4HB) copolyesters of low crystallinity revealed faster weight reduction in synthetic gastric juice and artificial colonic fluid than the highly crystalline homopolyester P(3HB) (Vodicka et al. 2022). A seminal study by Weng demonstrated the different biodegradation rates of P(3HB), P(3HB-*co*-3-mol-%-3HV), P(3HB-*co*-20-mol-%-3HV), P(3HB-*co*-40-mol-%-3HV), and P(3HB-*co*-10-mol-%-4HB) (all supplied by Ningbo Tianan Biomaterials Co. Ltd. China) under composting conditions according to the standard norm ISO 1855-1. These materials were processed to solvent-cast films and subjected towards conditions simulating an aerobic industrial composting process at a temperature of 58 °C ± 2 °C and constant aeration. Biodegradation was monitored via CO_2_ evolution. After 10 days, all films showed strong disintegration, with the less crystalline copolyesters disintegrating faster than the higher crystalline P(3HB). After 20 days, more than 50% of the films biodegraded for all types of PHA tested. The ultimate degree of biodegradation after 110 days of biodegradation according to ISO 14855-1 amounted to 90.5%, 89.3%,80.2%, 90.3% and 79.7% for P(3HB-*co*-40-mol-%-3HV), P(3HB-*co*-20-mol-%-3HV), P(3HB-*co*-3-mol-%-3HV), P(3HB-*co*-10-mol-%-4HB), and P(3HB), respectively. Lower crystallinity resulted in faster biodegradation under given conditions. Remarkably, cellulose, used as the biodegradable “positive reference” material showed a degree of biodegradation of 83.1%, lower than for P(3HB-*co*-40-mol-%-3HV), P(3HB-*co*-20-mol-%-3HV), and P(3HB-*co*-10-mol-%-4HB) (Weng et al. 2013).
- *c) Surface morphology*: Studies show that rougher surfaces of a biopolymer sample increase biodegradation compared to smoother surfaces. Wang et al. compared biodegradation of films made of Ecoflex, P(3HB), and P(3HB-*co*-3HHx) in activated sludge. Ecoflex, displaying the smoothest surface, degraded at a slower rate than P(3HB), and at much slower rate than P(3HB-*co*-3HHx) films, which had the roughest surface structure according to SEM studies. The synergistic action of low crystallinity and rough surface structure benefit biodegradation. Mechanistically, surface morphology impacts polymer degradation via enhanced contact between polymer chains and water, depolymerase enzymes, and microorganisms (Wang et al. 2004).
- *d) Environmental conditions*: Factors like pH-value, temperature, UV radiation, and humidity also strongly impact the rate of biodegradation. The correlation between these factors and biodegradation rates for both PHA homo- and heteropolyesters was postulated three decades ago by (Mergaert et al. 1993). The pH-value, as an abiotic degradation factor, is decisive in the reduction of the polymer’s molecular mass during the biodeterioration phase, which in turn makes the material more susceptible towards microbial and enzymatic attack in the subsequent biofragmentation phase (Jendrossek et al. 1996). Most importantly, ample presence of catalytically active microbes surrounding the PHA article is key for fast biodegradation. This was established in the 1990s, when Briese et al. (1994) noticed the fast and complete degradation of bottles consisting of BIOPOL® P(3HB-*co*-3HV) in highly microbially dense aerobic sewage sludge within three months, while much slower degradation of such bottles was observed in environments with less dense microbial activity, such as different soils, sludge from a lake or fresh water from the Danube River. Detailed studies by the authors showed that biodegradation of the polymer was highly dependent on the culture medium’s pH-value (maximum biodegradation for pH values between pH 7 and pH 8, very slow degradation below pH 6 and above pH 9) (Briese et al. 1994). In 2017, Prudnikova et al. studied the 16S rRNA genes isolated from the microorganisms degrading P(3HB), P(3HB-*co*-3HV), P(3HB-*co*-4HB), and P(3HB-*co*-3HHx). They found that the evolution of the PHA-degrading microbial community differs for different types of PHA when tested in the same environment (Prudnikova et al. 2017). Similar and more detailed results were reported soon afterwards by Volova et al. who demonstrated that, in the same soil quality, P(3HB) biodegradation was performed by members of the genera *Mitsuaria*, *Chitinophaga*, and *Acidovorax*. Copolyester biodegradation, in contrast, was catalyzed by *Roseomonas massiliae* and *Delftia acidovorans* in the case of P(3HB-*co*-3HV), by *Roseateles depolymerans*, *Streptomyces gardneri*, and *Cupriavidus* sp. for P(3HB-*co*-4HB), while *Pseudoxanthomonas* sp., *Pseudomonas fluorescens*, *Ensifer adhaerens*, and *Bacillus pumilus* were specific for P(3HB-*co*-3HHx) biodegradation (Volova et al. 2017). This was recently confirmed by Derippe et al. who noticed different microbial consortia building biofilms on different types of PHA and on cellulose incubated in the same sample of marine water; they observed that the microbial consortia that formed the biofilm on the specific PHA types were the most optimal ones to biodegrade those PHA types (Derippe et al. 2024).
- *e) Shape and thickness of a specimen to be biodegraded*: Thin films biodegrade faster than bulky granular materials, for P(3HB) and P(3HB-*co*-3HV) (Boyandin et al. 2013; Volova et al. 2010), and the biodegradation rate of pulverized PHA exceeded the rate of PHA film for the same PHA composition and crystallinity. A high surface-to-volume ratio generally favors faster biodegradation (Scandola et al. 1998). Article form is an important factor in their biodegradation and is a shortcoming of standardized biodegradation test methods: Most standard test methods are predominantly limited to specimens of small, standardized size, volume, and thickness, and typically do not match the real geometry and bulkiness of commercial articles made from PHA materials that need to be disposed of in composting or leaked to the environment. Morphology of polymer surfaces impacts biodegradability: Rougher surfaces make the PHA biopolymers more prone to microbial attack and result in better biofilm formation than smooth, compact surfaces, even those with higher overall crystallinity (Wen and Lu 2012). A recent paper by Komiyama et al. tested PHA biodegradability in diverse aqueous environments and in different forms: powder, cast films, undrawn fibers, and multifold-drawn fibers. They demonstrated that PHA in powder form displayed the fastest biodegradation, while multifold-drawn fibers degraded more slowly than undrawn fibers (Komiyama et al. 2021).
- *f) Mutual influence of different components in polymer blends on the degradation rate*: Biodegradable polymers are frequently blended together to produce articles for specific functional properties. It is pivotal to emphasize that the biodegradation behavior of such biopolymer blends has not been explored sufficiently. As Narancic et al. (2018) has shown, blends of PLA and poly(ε-caprolactone) (PCL) can be home composable, while PLA alone is only industrially compostable. Home compostability of P(3HB)/PLA (20/80) blendsis not described, but pure P(3HB) is home compostable. Similarly, P(3HB)/poly(butylene succinate) [PBS] (50/50) blends did not meet the home composting criteria, and neither blend readily degraded in marine, fresh water, and soil, nor in anaerobic aquatic digestion plants and under anaerobic degradation conditions. These findings deserve special attention because a thorough understanding of the biodegradability of “bioplastic blends” is of utmost importance for proper solid waste management, while such blends will most likely become more important in order to achieve functional properties for various in the future. As is emphasized by the authors, biopolymer blends *per se* will become materials of choice for various commercial articles to reduce plastic pollution but do not yet constitute a real panacea for this problem. This area of the science and technology of biobased and biodegradable materials requires further research in order to set-up a proper and holistic end-of-life management for these materials (Narancic et al. 2018). In this context, we should also consider that compostable and/or biodegradable polymers could result in problems if not sorted out of recycling streams consisting of other waste materials (Di Bartolo et al. 2021). Hence, it always must be kept in mind that challenges during the life cycle of PHA and other biopolymers and their blends thereof not only underly production, processing, and application, but, importantly, also end-of-life waste management (Saranya et al. 2020).

### Specific mechanisms involved in PHA biodegradation in different environments

The entire life cycle of replacement biobased and biodegradable materials needs to be assessed to be able to make a well-founded assessment on whether they are indeed more sustainable than established materials (Di Bartolo et al. 2021;  Narodoslawsky et al. 2015). In this context, the mechanisms under which biodegradation occurs, especially under realistic, natural conditions, deserve special attention. In principle, the biodegradation of all polymers is dependent on the susceptibility of the polymer’s backbone to microbes and their secreted enzymes’ ability to reach the backbone and can occur via two mechanisms: via surface erosion or via bulk erosion. However, biodegradation of polymers like PHA predominantly occurs at the surface, where depolymerase enzymes (phaZ) attack the polyester chains. These enzymes are too bulky to penetrate the crystalline polymer structure, which contrasts with degradation driven by other factors, such as by small chemical molecules or radicals (Meereboer et al. 2020). For PHA, it was shown by Doi et al. that mechanistically, all *scl*-PHA samples were degraded via surface erosion, where depolymerase enzymes first attack the amorphous P(3HB) regions on the surface of a polymer sample; after that, crystalline P(3HB) regions are depolymerized (Doi et al. 1990). As has been described by Jain and Tiwari (2015), “*the biodegradation efficiently begins from surface and gradually wrap the inner molecular conformation*”. Similar conclusions were drawn by Arcos-Hernandez et al., who observed that during ongoing P(3HB-*co*-3HV) biodegradation in soil, molecular mass and polydispersity (molecular mass distribution) of the remaining undegraded PHA samples did not drastically change. PHA biodegradation was unambiguously confirmed via CO_2_ evolution measurement (Arcos-Hernandez et al. 2012). Boyandin et al. (2013) also noticed an increase in percent crystallinity of the remaining PHA samples during degradation when buried in tropical soil; these authors ascribed this finding to the initial consumption of the amorphous regions of the polymer, leaving behind the crystalline phase (Boyandin et al. 2013). Recently, Prudnikova and colleagues confirmed an increase of P(3HB) crystallinity and decrease of molecular mass during biodegradation in soil, which was attributed to the preferential degradation of the amorphous regions by microbes (Prudnikova et al. 2024). Non-crystalline and hence amorphous PHA biopolymers are sufficiently flexible to allow water and enzyme penetration into the interior of the material. This was observed for completely amorphous *mcl*-PHA which undergo biodegradation via bulk erosion (Ho et al. 2002). While there are a numerous of studies reporting *scl*-PHA biodegradation under most diverse conditions there are only few reports of biodegradation studies of *mcl*-PHA, especially under realistic environmental conditions. Interestingly, Keridou et al. recently elucidated that degradation of P(4HB) homopolyester follows two different mechanisms depending on the degradation conditions: While compression-molded P(4HB) films exposed to abiotic hydrolytic degradation at different pH- and temperature conditions underwent bulk degradation and random chain scission, those samples subjected to enzymatic attack displayed surface erosion and depolymerization, typical for PHA biodegradation (Keridou et al. 2021). This matches well with recent studies by Vodicka et al., who observed degradation of P(3HB), P(3HB-*co*-36-mol-%-4HB), and P(3HB-*co*-66-mol-%-4HB) films (11 µm thickness for each sample) in simulated body fluids. When incubated in simulated gastric fluid, samples were disintegrated by rapid hydrolysis due to the high acidity (pH-value 1.6), hence, via abiotic mechanism. In contrast, incubation in artificial colonic fluid (pH-value 7.8) resulted in biodegradation via enzymatic hydrolysis in a surface erosion process, monitored by microscopic observation (Vodicka et al. 2022).

**Fig. 4:**
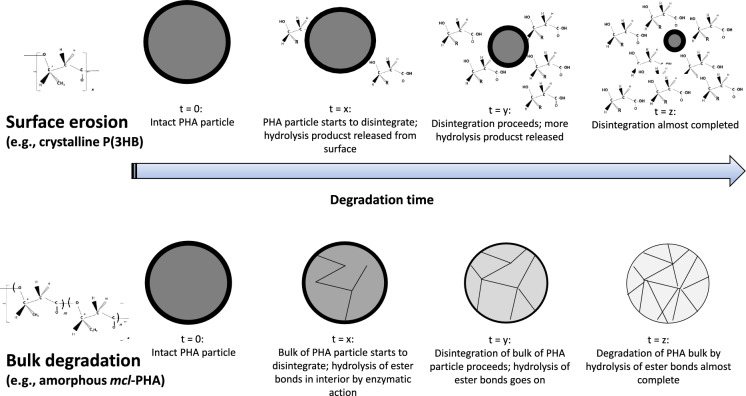
Schematic of surface erosion (upper part) *vs.* bulk degradation (bottom part)

Figure 4 illustrates schematically the difference between surface erosion and bulk degradation.

In principle, extracellular PHA hydrolysis occurs in two phases: First, enzymes (extracellular PHA depolymerases, e-PhaZ) are adsorbed on the PHA surface through the enzyme’s active sites. Second, the hydrophobic domain binding site and the catalytic site of the enzymes induce hydrolytic cleavage of PHA, releasing oligomers of PHA building blocks into the environment, which are then assimilated by organisms, and subsequently undergoes mineralization inside the cells as the terminal reaction.

### Biodegradability and compostability test methods, standards and certifications used for PHA

As Narancic et al. (2020) has emphasized, it is of utmost importance to understand type of waste management options available for the biodegradation of biopolymers like PHA and others including blends. This understanding is essential in designing articles with biobased biodegradable materials to replace single use plastics in a sustainable manner, and ultimately to avoid further accumulation of plastic waste. Biodegradability of biopolymers and their blends must be assessed in a variety of diverse conditions including available organized waste management sytems and natural environments at various temperatures and other abiotic stresses, and the timeframe. This is where standards and norms come into play, and are described in the subsequent section. The underlying test schemes clearly define the biodegradation conditions and timeframes to be used and specify the degree of biodegrdation that needs to be achieved. Importantly, it should be noticed that currently employed temperatures for tests according to established international ISO and EN biodegradation standards are higher than temperatures in “real”, unmanaged environments. This and other specific conditions needs to be revisited when developing standards and certifications on which policies for plastic waste management can then be implemented (Narancic et al. 2020).

The following test standards are used most widely and deserve to be discussed:

The European Norm for compostability of packaging (EN 13432) is considered the “gold standard” used for characterizing a “plastic” as “biodegradable”; it requires, in addition to those criteria mentioned above a clear and detailed description of the product, and a total of four replicate tests is required to be carried out:

- Test on biodegradation: chemical break down of the polymer. The certification requirement is that 90% of carbon gets converted to CO₂ at 28 °C within 1 year.
- Test on disintegration: The physical falling apart of the material into small fragments. The certification requirement for quantification is 90% disintegration of the product at 28 °C within 6 months. Because the disintegration process is strongly dependent on the material’s thickness, texture, and/or density, the materials and products are to be certified up to a certain thickness only.
- Test on ecotoxicity: Does the composted product exert any negative effect on plants? Plant toxicity testing is part of all standards for industrial and home compostability and prescribes the use of two plant species. In contrast, earthworm toxicity testing is needed to get certified under the Australian standard AS 5810.
- Test on heavy metals and fluorine content. Note: Each biodegradation standard has its individual heavy metal limits, with EN 13432 and AS 4736/5810 (Australia) being the most stringent.

Here, it should be considered that “biodegradability”, “ecotoxicity” and “heavy metal content” are material characteristics, while “disintegration” is a characteristic of both material and final product shape.

Most recently, the health issues related to the presence of perfluoroalkyl compounds (PFAS) has come to light. This class of compounds are used mostly during the processing of plastics, paper or a combination of the two as a process aid. There is a huge push to eliminate their use and most producers have ceased production. While individual tests to identify their presence exist, systematic and standardized testing of such compounds are embryonic and needs to become the norm (Müller et al. 2025).

Industrially compostable: Products certified “OK compost INDUSTRIAL-certified” are those that compost only in industrial composting facilities, at temperatures between 55 and 60 °C. Therefore, such products should not go into home compost (online resource 1) facilities. This contrasts with “Home composting”, a process occurring at ambient temperature, resulting in a slower conversion of organic carbon present in the biodegradable or compostable material to CO₂ and water. Home composting generally occurs at a slower pace compared to industrial composting. Defining and monitoring home composting is not trivial; therefore, there is noticeable lack of regulation in this area. Indeed, the first “home composting” standard was not published until 2010, in Australia, some decades after the first standards for “industrial composting” were already established. Importantly, one should keep in mind that, in case “home composting” is not properly performed, even “home compostable” certified products could fail to compost. Consequently, appropriate training of end-consumers on home composting processes and compost application (proper use of generated compost) is necessary. In any case, “home composting” of “home compostable” biomaterials makes sense: It reduces the volume of mixed plastics to be managed through established waste management systems. Moreover, it empowers the end-consumer to be responsible for the separation, treatment and the ultimate end-use of the biomaterial waste, thus contributing to increased public awareness and involvement in waste management (online resource 2).

It should be noted that until 2015, no EU-wide standard for “home composting” was available. The need for such dedicated standard for home compostable packaging was emphasized by the European Parliament and the Council of the EU in a Directive of April 2015 amending the Packaging and Packaging Waste Directive (PPWD). This Directive (EU) 2015/720 aims at minimizing the consumption of lightweight plastic carrier bags, which are frequently used in households to collect and carry garden and kitchen waste to home composting piles in private gardens (online resource 3). It was not before 2020, when the norm prEN 17427 (“*Packaging—Requirements and test scheme for carrier bags suitable for treatment in well managed household composting plants*”) was introduced (note: here, the term “well-managed household composting plants” is emphasized, underlining the need for appropriate education of end-consumers). The following four aspects are addressed by this norm: (a) characterization; (b) biodegradation in well managed home composting; (c) disintegration in well managed home composting; and (d) home compost quality. This norm specifically refers to the end-of-life fate of “plastic” bags. Ultimate biodegradation of the test material needs to take place when exposed to microbes active under mesophilic conditions (between 15 °C and 45 °C), not under industrial composting conditions (online resource 4). PHA fulfills the home composting criteria according to norm prEN 17427.

Before the establishment of norm prEN 17427, a range of different standards and associated certificates regulated bioplastics’ “home compostability” on national levels, in most cases rooted in above-discussed norm EN 13432. A “home compostability” certification scheme is offered, e.g., by the certification institution Vinçotte. For home compostable packaging, TÜV Austria awards the “Ok compost HOME” label based on Vinçotte’s certification scheme (online resource 5). This is in addition to TÜV Austria’s “OK biodegradable” label awarded for certified biodegradability in soil, fresh water and marine water, respectively (online resource 6). Hence, different certifications are required to make a bioplastic material both “biodegradable” and “home compostable”, two characteristics which should not be confused. Importantly, TÜV Austria´s “Ok compost HOME” certification does not constitute a specific standard but is rather a list of all the technical demands required by a packaging material to obtain this certification. “Ok compost HOME” can be considered the basis of subsequent “home compostability” standards established in other countries. As an example, the Australian standard AS 5810 (“*Biodegradable plastics—Biodegradable plastics that are suitable for home composting*”) from 2010 is also based on TÜV Austria´s “Ok compost HOME” (online resource 7), while DIN CERTCO, in turn, provides the NF T51-800 certification for “home compostability” based on AS 5810 (online resource 8). In Italy, UNI 11183:2006, a national standard for “*composting at ambient temperature*” (online resource 9), while in 2015, the French Standard “*NF T 51-800 Plastics—Specifications for plastics suitable for home composting*” were introduced, which also constitute part of the DIN CERTCO certification scheme and complies with the Australian standard AS 5810 (online resource 10).

In the last decade several standards have come into existence to determine and categorize biodegradability and compostability of materials under different conditions (aerobic/anaerobic, industrial/home, etc.). These standards prescribe the test schemes that need to be applied to evaluate and determine the compostability and biodegradability of “biomaterials” such as PHA or cellulose- or starch-based plastic-like materials. In general, those standards comprise the requirements to test parameters regarding the characterization of the material (e.g., chemical composition like the assessment of heavy metal levels), its disintegration ability, its aerobic biodegradation into CO_2_, biomass and water within a defined period (typically 6 months), anaerobic digestion for CH_4_ and CO_2_ formation, and ecotoxicity. Bioplastics certified according to EN 13432 can be recognized by conformity marks such as the “Seedling”, “OK compost”, or “DIN geprüft”. Standards and specifications have been developed by several authorities such as the European Committee for Standardization (EN), the American Society for Testing and Material (ASTM), the International Organization for Standardization (ISO), the British Standard Institution (BSI), etc. In Italy, UNI 11183:200622, a national standard exists relating to the suitability of biodegradable plastics for composting at ambient temperature (online resource 9). EN ISO standards, in turn, were originally issued by ISO, and later implemented on the European level by the European Committee de Normalisation (CEN). Moreover, UNI 11183:200622 specifies the biodegradability requirements of plastic materials which are utilized to make products disposable through home composting, i.e., via aerobic biodegradation at ambient temperature (21 °C to 28 °C) (online resource 9).

#### Additional standards of use to test biodegradability of materials

ASTM D5338 (“*Standard Test Method for Determining Aerobic Biodegradation of Plastic Materials Under Controlled Composting Conditions, Incorporating Thermophilic Temperatures*”) is a standard biodegradation test that measures aerobic biodegradation of materials under controlled composting conditions. Here, the material to be tested is exposed to a mixed bacterial and fungal inoculum for a minimum of 90 days; respirometry is applied to measure biodegradation (online resource 11).

ASTM D6400 (“*Standard Specification for Labeling of Plastics Designed to be Aerobically Composted in Municipal or Industrial Facilities*”): This globally recognized biodegradation test standard is most often used to test composting of materials including paper, textiles, and foams. It favors thermophilic microorganisms thriving above 50 °C, hence, it is a test standard for industrial composting. ASTM D6400 is aligned with ISO 14855 (online resource 12).

ISO 14855 (“*Determination of the ultimate aerobic biodegradability of plastic materials under controlled composting conditions—Method by analysis of evolved carbon dioxide*”) constitutes a biodegradation test method that determines the ultimate aerobic biodegradability and disintegration of plastic materials under controlled composting conditions via CO_2_ evolution. ISO 14855 requires testing for a minimum of 90 days (online resource 13). For example, Weng et al. provided a detailed biodegradation study for different types of commercialized PHA (P(3HB), P(3HB-*co*-3HV), P(3HB-*co*-4HB)) based on this test method, showing that all these commercially available biopolyesters confirm to this norm (Weng et al. 2011).

ISO 16929 (“*Plastics—Determination of the degree of disintegration of plastic materials under defined composting conditions in a pilot-scale test*”): This standard constitutes a standard composting method that determines the degree of disintegration of materials in a pilot-scale test under defined composting conditions. Importantly, it works in a realistic compost environment more representative of microbial populations than laboratory-scale degradation set ups, and more realistic than ISO 14855. Also, under the conditions used for this standard, thermophilic microorganism thriving above 50 °C are key, hence, it is also considered an industrial composting standard. ISO 16929 typically runs for 12 weeks and is frequently needed for consumer- and agricultural goods such as plastics, tarps, and textiles (online resource 14).

ISO 17556 *(“Plastics—Determination of the ultimate aerobic biodegradability of plastic materials in soil by measuring the oxygen demand in a respirometer or the amount of carbon dioxide evolved”*): This standard specifies a method for determining the ultimate aerobic biodegradability of plastic materials in soil by measuring the oxygen demand in a closed respirometer or the amount of carbon dioxide evolved. The method is designed to yield an optimum degree of biodegradation by adjusting the humidity of the test soil. The maximum testing period amounts to 2 years, using standard soil consisting of a mixture of 70% industrial quartz sand, 16% natural soil, 10% kaolinite clay, plus 4% mature compost.

ISO 14853 (“*Plastics—Determination of the ultimate anaerobic biodegradation of plastic materials in an aqueous system—Method by measurement of biogas production*”). This standard specifies a method for the determination of the ultimate anaerobic biodegradability of plastics by anaerobic microorganisms. The conditions described in ISO 14853 do not necessarily correspond to the optimum conditions for the maximum degree of biodegradation to occur. The test calls for exposure of the test material to sludge for a period of up to 90 d, which is longer than the normal sludge retention time (25 to 30 d) in anaerobic digesters, although digesters at industrial sites can have much longer retention times.

ISO 14593 (“*Water quality—Evaluation of ultimate aerobic biodegradability of organic compounds in aqueous medium—Method by analysis of inorganic carbon in sealed vessels (CO*_*2*_* headspace test)*”) is a biodegradation test in solution that evaluates ultimate aerobic biodegradability of organic compounds in an aqueous medium. This method, however, is rather applied for inorganic carbon compounds than for biopolymers (online resource 15).

ASTM D 5988-96 is the “*Standard Test Method For Determining Aerobic Biodegradation In Soil Of Plastic Materials Or Residual Plastic Materials After Composting*”. It determines the degree and rate of aerobic biodegradation of plastics (including formulation additives that may be biodegradable) in contact with soil, or a mixture of soil and mature compost, under laboratory conditions (online resource 16).

For biodegradation in aquatic environments, specific standards apply. This is of major importance since plastic pollution of fresh- and marine water is of the utmost concern of the Anthropocene. Such standards and norms for plastic biodegradation in water were reviewed by Lavagnolo et al., and are briefly summarized here as follows (Lavagnolo et al. 2023):

ISO 14851 (“*Determination of the ultimate aerobic biodegradability of plastic materials in an aqueous medium—Method by measuring the oxygen demand in a closed respirometer*”): The test material is incubated in a chemically defined liquid medium, essentially free of organic compounds; the medium gets spiked with microbes from activated sludge. Incubation occurs for a minimum of 28 days at 21 °C under dark conditions. Evolved CO_2_ is absorbed by a KOH solution and determined via titration.

EN ISO 14 852:2021: Determines the ultimate aerobic plastics’ biodegradability in an aqueous medium by analysis of evolved CO_2_.

EN ISO 18 830:2017: *Plastics—Determination of aerobic biodegradation of non-floating plastic materials in a seawater/sandy sediment interface*. Measures the oxygen demand in closed respirometers.

EN ISO 19 679:2020: *Plastics—Determination of aerobic biodegradation of non-floating plastic materials in a seawater/sediment interface*. Measures evolved CO_2_.

EN ISO 22 404:2021: *Plastics. Determination of the aerobic biodegradation of non-floating materials exposed to marine sediment*. Measures evolved CO_2_.

EN 14 047:2002: *Packaging. Determination of the ultimate aerobic biodegradability of packaging materials in an aqueous medium*. Measures evolved CO_2_.

EN 14 047:2002: Packaging. Determination of the ultimate aerobic biodegradability of packaging materials in an aqueous medium. Measures the oxygen demand in closed respirometers.

ASTM D6691: This standard determines biodegradation under laboratory conditions by incubation in natural marine water enriched with inorganic nutrients and containing indigenous microorganisms. Incubation occurs at 21 °C for a minimum of 28 days in the dark. Generated CO_2_ is absorbed by an aqueous KOH solution and determined via titration.

ASTM D7081-05: Standard specifications for non-floating biodegradable plastics in the marine environment.

ASTM D7991-22: Standard test method for determining aerobic biodegradation of plastics buried in sandy marine sediment under controlled laboratory conditions.

**Fig. 5:**
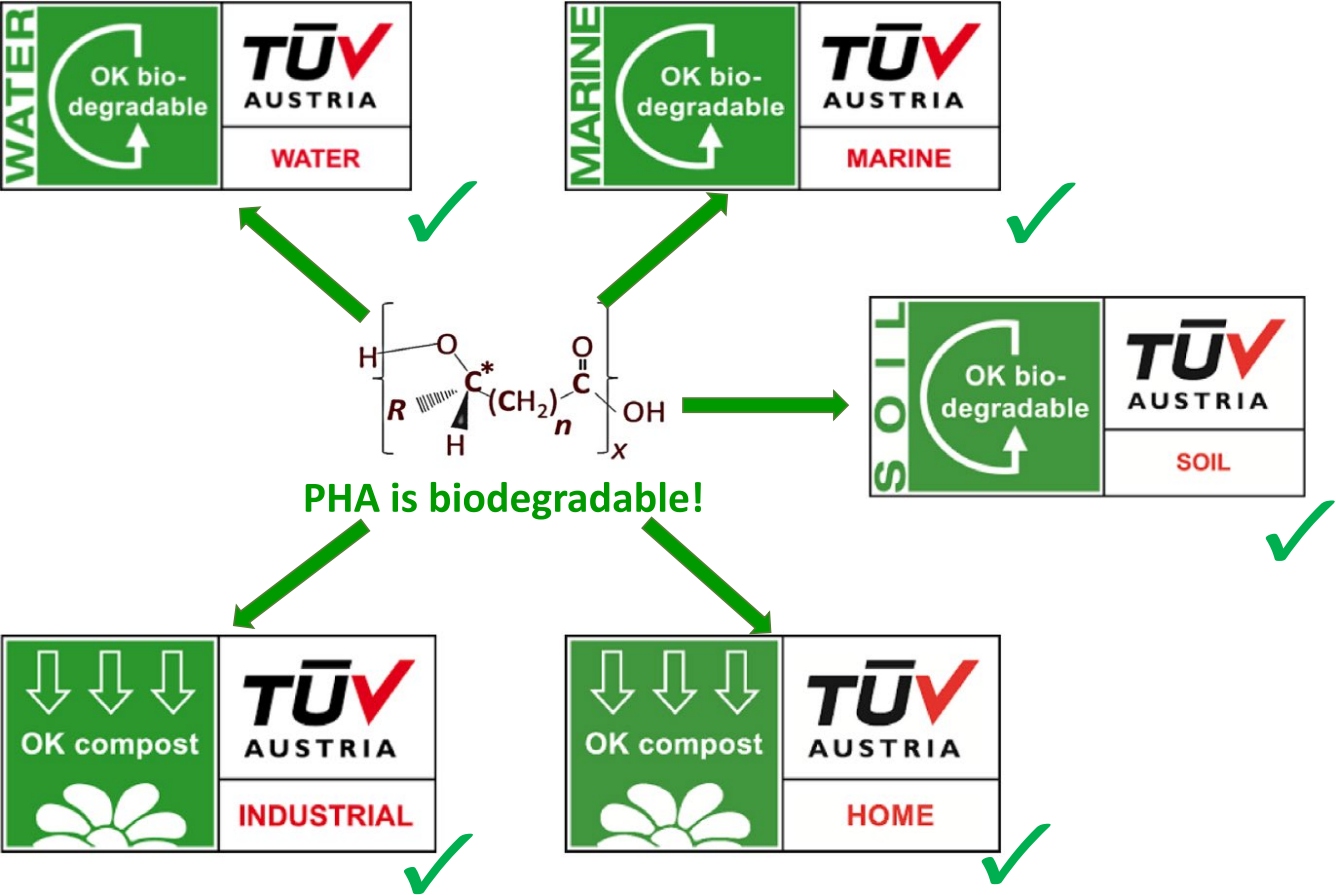
Biodegradation of PHA according to TÜV Austria certifications awarded to industrially produced PHA biopolyesters [based on (online resource 17)]

Biodegradability and compostability of PHA biopolyesters have been scrutinized under diverse environments and test conditions, i.e., soil, water, marine, as well as industrial and home composting. PHA producing companies offering such products all have certifications for PHA materials that correspond to the above-mentioned standards through certification bodies such as DIN CERTCO, BPI and TÜV Austria that verify biodegradability and compostability claims of each product and are advertised with the certification body’s logos and labels. Figure 5 visualizes what makes PHA “biodegradable” in the context of TÜV Austria certifications awarded to industrially produced types of PHA.

## Case studies for PHA biodegradation in given environments

As mentioned earlier, biodegradation of different types PHA has been broadly studied, and their biodegradability have been well established in the scientific community. Research has typically focused on screening for PHA-degrading enzymes from diverse microorganism under controlled and optimized conditions in laboratory settings, however, insufficient attention has been dedicated to monitoring biodegradation performance in “real” and dynamic environmental settings such as the ocean, fresh water, home composting, and different types of soil (Kim et al. 2023). In 1995, Mas-Castellà et al*.* emphasized that “*laboratory conditions set up to run biodegradability tests do not totally represent the natural environmental conditions that plastic waste may encounter*” (Mas-Castellà et al. 1995). As a prime example, the number of studies testing P(3HB-*co*-4HB) biodegradation under optimized conditions by isolated enzymes is already unmanageable; even comparing and discussing the most seminal studies among them would exceed to scope of present review. What is needed for a straightforward assessment of the end-of-life fate of biomaterials like PHA in the environment, studies under “real” conditions, such as in different types of soil and water, without the addition of excessive amounts of depolymerase enzymes under optimized, non-realistic conditions. Especially for the medically relevant homopolyester P(4HB), such “real life” biodegradation studies are simply not available, despite the fact that P(4HB) indeed is confirmed to be readily biodegradable *in vivo* (Stock et al. 2000), with an absorption rate of 8–52 weeks (Martin and Williams 2003), which enabled the FDA approval for Tepha’s strong and flexible P(4HB) sutures and meshes in 2007 (Guo and Martin 2015). Moreover, enzymatic biodegradability of P(4HB) under optimized laboratory conditions is also well established (Keridou et al. 2021; Su et al. 2003).

The rule of thumb is that PHA biodegradability in aqueous environments, both fresh water and marine environments is slower compared to that in soil  and activated wastewater sludge, since water provides a strong dilution of catalytic microorganisms in comparison to soil and activated sludge (Kliem et al. 2020).

The following sections compile available data on PHA degradation in relevant “real” environments, reporting not only on the type of PHA used, PHA´s origin (production strains, manufacturers), the exact environmental test conditions (as disclosed in the original literature), the shape and geometry of the PHA specimens tested, but, importantly, also on the method used to monitor PHA degradation. It must be stressed that most studies restrict degradability assessment that are based on mass loss determination, which is well established and convenient, but also associated with ambiguities due to eventual mechanical fragmentation processes. Other studies, in contrast, report CO_2_ evolution measurement, or O_2_ depletion when aerobic degradation is studied. This demonstrates that standards and norms to assess biodegradability of a material are important especially when comparing materials across different end-of-life environments. Even more sophisticated and precise approaches to determine the fate of degraded materials determine evolution of ^13^C- or ^14^C-labeled CO_2_ from isotopically labeled test specimens.

In addition, this section also includes key recent works on biodegradation of PHA blends in different environmental settings.

Studies detailed in the subsequent sections are summarized in Table 2.

**Table 2:** Case studies for quantification of PHA samples of different composition and shape in realistic environments

Type of PHA	Environment	Origin	Environment	Shape of test specimens	Test conditions (how was degradation monitored?)	Degree of degradation /degradation rate	Ref.
P(3HB)	P(3HB)						
P(3HB)	Soil	Probably produced according to the process developed by Imperial Chemical Industries (ICI), UK	Two different soil qualities; periodical water addition to maintain humidity	Films	Measuring radioactive CO_*2*_ gas evolution from ^14^C-labeled P(3HB)	70% after 18 weeks in soil quality 1, about 30–35% in soil quality 2	Luzier (1992)
P(3HB)	Soil	ICI, UK	Sandy soil, clay soil, loamy soil, hardwood forest soil, and pinewood forest soil; 15, 28 and 40°C, water content of soil 12–44% RH, constant pH-value	Injection-molded, dumbbell-shaped tensile-test pieces (83 mm × 2 mm, mass 1.75 g)	Mass loss determination	At 15 °C: daily mass loss of P(3HB) amounted to about 0.05, 0.03, 0.06, 0.12, and 0.10% in hardwood-, sandy-, pinewood-, clay-, and loamy soil, respectively	Mergaert et al. (1993)
						At 28 °C: 0.12, 0.06, 0.12, 0.10, and 0.04 for the same soil qualities	
						At 40 °C: 0.14, 0.29, 0.21, 0.48, and 0.46%	
P(3HB)	Soil	Imperial chemical industries (ICI), UK	Forest soil covered with fallen leaves, sandy soil on a riverbank, activated sludge soil located near a WWTP, and farm soil 28, 37, and 60 °C tested	Thin solvent-casting sheets	Mass loss determination	After 25 days: 98.9% in activated sludge soil at 37 °C 68.8% in farm soil at 37 °C Sandy soil and forest soil: 10% and 7% at 37 °C 68.9% in activated sludge soil at 28 °C 41.3% in farm soil at 28 °C Sandy soil and forest soil: 5.8% and 10.5% at 28 °C 60 °C: 4.9, 4.5, 30.5, and 14.8% for in forest-, sandy-, activated sludge-, and sludge soil	Mergaert et al. (1993)
P(3HB)	Soil	*C. eutrophus* B10646, Russian Academy of Sciences	“Agro-transformed field soil, village Minino, Krasnoyarsk”; 35 days, 28 °C, 50% humidity	Films (no info in size, thickness and mass)	Mass loss determination	After 21 days: 60% mass loss	Prudnikova et al. (2017)
						After 35 days: about 90% mass loss	
P(3HB)	Soil	Goodfellow, USA	Soil with 40 and 100% relative humidity, RH	Solvent-cast films, about 15 µm thickness	Observation of changes in appearance, chemical signatures, mechanical properties, and molecular mass	Complete degradation after 2 weeks in soil with 100% humidity; significant reduction of mechanical properties after 3 days	Kim et al. (2023)
						40% humidity: no excessive changes after 6 weeks	
P(3HB)	Soil	Imperial chemical industries (ICI), UK	Soil from the coastal area near Ravenna, Italy. 22 ± 3 °C	Powder and thin compression-molded films	CO_2_ evolution according to ASTM D 5988-96	Complete degradation in powder form after less than 3 months	Modelli et al. (1999)
						After 60 days: 20 mmol CO_2_ released from powder, about 2 mmol from compression-molded film	
P(3HB)	Soil	*Wautersia eutropha* B5786, Russian Academy of Sciences	Soil at two different climate test stations in Vietnam; depth 15 cm	Solvent-cast films, about 10 µm thickness and granules	Mass loss and crystallinity determination	After 10 months: films at more humid test stations completely degraded; granules degraded by 55%. Daily loss of 0.33 and 0.13% for films and granules, respectively	Boyandin et al. (2013)
						After 12 months: films at less humid test stations degraded by 47%; granules degraded by 28%. Daily loss of 0.18 and 0.08% for films and granules, respectively	
P(3HB)	Soil	*C. necator*	Garden soil, 6 months; no environmental conditions reported	Solvent-cast films; thickness not reported	Mass loss determination	64.3% in 6 months	Jain and Tiwari (2015)
P(3HB)	Soil	Metabolix Inc.	Soil mixture of 43% certified organic topsoil, 43% no-till farm soil, and 14% sand; 60% humidity	Injection-molded films of 0.6 mm thickness	CO_2_ evolution according to ASTM D5988-03	After 660 days: about 70% (only slightly lower than for cellulose)	Gomez and Michel Jr. (2013)
P(3HB)	Soil	Sigma Aldrich	Garden of Biological School of University Sains, Malaysia	Solvent-casted P(3HB-*co*-4HB) films (1.2 × 1.2 cm, strongly varying thickness from 0.3 to 6 mm, average mass 0.02 g)	Mass loss determination	After 5 weeks: about 43% degradation	Vigneswari et al. (2019)
P(3HB)	Soil	Fluka	Acidic forest soil well-shaded under fallen decomposing leaves (FS), unshaded alkaline forest soil along of a freshwater stream (FSst), and brackish mangrove soil (MS); Malaysia Films buried in 2 cm depth	Solvent-cast films	Mass loss determination, observation of surface erosion, molecular mass determination, thermophysical data	After 112 days in soil: 4.6% in FS, 73.5% in FSst, and 100% in MS)	Lim et al. (2005)
P(3HB)	Soil	PHB Industrial S/A, Brazil	“Intermediate mangrove compartment” in George Town, Malaysia, at the estuary of the Pinang River. Degradation on surface and after burial	Solvent-cast films	Microscopic observation of film degradation, mass loss determination	About 50–70% mass loss after eight weeks for all tested surface conditions. For buried samples, mass loss amounted to 80 to almost 100% after 8 weeks	Sridewi et al. (2006)
P(3HB)	Soil	Microbial P(3HB), synthetic P(3HB), and “syndio-rich P(3HB)”	Soil (room temperature, pH-value 7.4); 150 g soil plus 41.8 mL water and 0.538 g PHA per test bottle		According to ASTM D2980-17E0; measuring the CO_2_ released into the headspace of test bottles	Biodegradation after 90 days: About 80, 54, and 70% for microbial, “syndio-rich” and synthetic P(3HB), respectively	Quinn et al. (2023)
						According to calculations: 90% for “syndio-rich”, synthetic, and microbial P(3HB) after 268, 145, and 105 days, respectively	
P(3HB)	Soil	*Cupriavidus eutrophus* B10646, Russian Academy of Sciences. Glucose as carbon source	“Agro-transformed soil”, humus-rich and slightly alkaline, collected in the temperate zone of Siberia	Polymer disks (solvent-casted) 30 mm in diameter, 0.035–0.045 mm thickness, and 35 ± 5 mg of mass	Mass loss determination, change of crystallinity and molecular mass	21 °C: about % mass loss after 35 days 28 °C: 97% mass loss after 35 days	Volova et al. (2017)
						Degree of crystallinity: constant for 35 days, insignificant change of molecular mass and polydispersity	
P(3HB)	Soil	*C. eutrophus* B10646, Russian Academy of Sciences	Soil in Eastern Siberia (“chernozem soil”: fertile black soil) and “ref ferralitic soil” under tropical conditions in India	Solvent-cast films (0.035–0.045 mm thickness, mass 35 ± 5 mg)	Mass loss determination, change of crystallinity and molecular mass	50% mass loss after 64.8 days in Siberian soil, and 126.4 days in Indian soil	Prudnikova et al. (2024)
P(3HB)	Soil	Enmat Y 1000, Tianan Biological Materials Co. Ltd., PR China	Soil consisting of 1/3 field soil and 2/3 forest soil	Melt-processed samples produced via compression-molding to 20 × 20 × 0.2 rectangular specimens	According to ISO 17556; determination of the amount of CO_2_ evolved by CO_2_ absorption in KOH solution	Complete biodegradation	Narancic et al. (2018)
P(3HB)	Compost	Ningbo Tianan Biomaterials Co. Ltd., PR China	According to ISO 14855-1:2005; aerated composting vessel at 58 °C ± 2 °C	Solvent-cast films	Macroscopic observation of film degradation, CO_2_ evolution	79.7% biodegradation after 110 days (cellulose reference: 83.1%)	Weng et al. (2011)
P(3HB)	Fresh water	Imperial chemical industries (ICI), UK	Natural freshwater ponds	Injection-molded, dumbbell-shaped tensile-test pieces (83 mm × 2 mm, mass 1.75 g	Mass loss determination	After 6 months 7% mass loss	Mergaert et al. (1995)
						After 1 year 34% mass loss	
P(3HB)	Fresh water	Goodfellow	Rainwater collected from ponds at University of Bayreuth, Germany. Illuminated by 16h/8h light/dark cycles, constant temperature of 25 °C	Films obtained by hot pressing (compression molding)	Mass loss determination	About 8.5% mass loss after 1 year	Bagheri et al. (2017)
P(3HB)	Fresh water	Sigma Aldrich	Lake near the School of University Sains, Malaysia	Solvent-casted P(3HB-*co*-4HB) films (1.2 × 1.2 cm, strongly varying thickness from 0.3 to 6 m, average mass 0.02 g)	Mass loss determination	After 5 weeks: about 43% degradation	Vigneswari et al. (2019)
P(3HB)	Fresh water	Microbial P(3HB), synthetic P(3HB), and “syndio-rich P(3HB)”	Soil (25 °C, pH-value 7.2); 7.2 mg activated sludge was mixed with 192.8 mL mineral salt solution and 16.9 mg PHA samples per test bottle	Not reported	According to ISO14851; measuring the CO_2_ released into the headspace of test bottles	Biodegradation after 90 days: About 50% for all tested samples. According to calculations: 90% for “syndio-rich”, synthetic, and microbial P(3HB) after 383, 433, and 282 days, respectively	Quinn et al. (2023)
P(3HB)	Fresh water	Enmat Y 1000, Tianan Biological Materials Co. Ltd., PR China	Defined aqueous medium at 21 °C with microbes from activated sludge	Melt-processed samples produced via compression-molding to 20 × 20 × 0.2 rectangular specimens	According to ISO 14851. Determination of the amount of CO_2_ evolved by CO_2_ absorption in KOH solution	Almost complete degradation after 28 days	Narancic et al. (2018)
P(3HB)	Marine	P(3HB) produced by *C. necator* from butyric acid	Marine water at Kanagawa Prefectural Fishery Experiment Station, Jogashima (Japan) in outdoor tank (1.5 m water depth, 22 + 3 °C)	Thin solvent-casted films (thickness 50–150 µm, 5 × 10 cm in size)	Surface erosion	About 12 µm surface erosion after three weeks	Doi et al. (1992a, b)
P(3HB)	Marine	Imperial chemical industries (ICI), UK	Seawater in the harbor of Zeebrugge, Belgium	Injection-molded, dumbbell-shaped tensile-test pieces (3 mm × 2 mm, mass 1.75 g)	Mass loss determination	31% mass loss within 270 days of submersion; no molecular mass changes	Mergaert et al. (1995)
P(3HB)	Marine	Metabolix	a) inoculum consisting of 13 marine microbial species at 30 °C	Screw-extruded films 1.27 × 1.27cm in size	a) ASTM 6691 (respiratory measurements)	a) 70% mineralization after 40 days, 80% after 100 days	Thellen et al. (2008)
					b) Mass loss determination	b) static conditions: 90% and 20–30% mass loss after 18 days with and without addition of sea sediment. Dynamic conditions: 50% and 20% mass loss after 63 days with and without addition of sea sediment	
			b) natural seawater at 21 °C				
P(3HB)	Marine	*Ralstonia eutropha* B5786, Institute of Biophysics SB RAS, Russia	Tropical marine environments in the South China Sea (27 °C–30° C)	Thin solvent-casted films (0.1 mm thickness, 30 mm diameter, 73 mg mass) and as compacted pellets	Mass loss determination	42% mass loss after 180 days for films. Pellets: degradation started after 80 days (about 40% mass loss after 180 days)	Volova et al. (2010)
P(3HB)	Marine	Goodfellow	Artificial seawater from a coral reef aquarium	Films obtained by hot pressing (compression molding)	Mass loss determination	About 6% mass loss after 1 year	Bagheri et al. (2017)
P(3HB)	Marine		Natural dynamic and static sea water at Akabane Fishing Port, Japan; 19–26 °C	Thin films (50 µm thickness)	Mass loss determination	After 2 weeks: 60% mass loss under dynamic conditions, about 5% mass loss after 5 weeks	Tsuji and Suzuyoshi (2022)
P(3HB)	Marine	P(3HB) produced by *Halomonas* sp. SF2003 on glucose	Marine water (Northwest Atlantic) and microbial consortium previously detached from PHA samples incubated in sea water	Solvent-casted P(3HB) disks of 6 mm^2^ diameter	Oxygen consumption	4.5 × 10^–3^ µmol O_2_ per mm^2^ P(3HB) surface after 2 months of incubation	Derippe et al. (2024)
P(3HB)	Marine	P(3HB) from ICI	Bathyal seafloor off Misaki port in the northern Pacific Ocean, Japan. 757 m depth	Uniform spherical microbeads 50–150 µm in size, prepared via melt homogenization	Mass loss determination	45% mass loss after 5 months in deep sea	Hyodo et al. (2024)
P(3HB)	Marine	P(3HB) from ICI	Seawater and soil samples collected from Tokyo Bay. 5 L of seawater mixed with 1 kg of soil; 27 °C	Uniform spherical microbeads 50–150 µm in size, prepared via melt homogenization	BOD	85% degradation after 25 days (reference cellulose: 77%)	Hyodo et al. (2024)
P(3HB)	Marine	Enmat Y 1000, Tianan Biological Materials Co. Ltd., PR China	Incubation in seawater from Belgium at 30°C	Melt-processed samples produced via compression-molding to 20 × 20 × 0.2 rectangular specimens	According to ISO 14851. Determination of the amount of CO_2_ evolved by CO_2_ absorption in KOH solution	Almost complete degradation after 28 days	Narancic et al. (2018)
P(3HB)	Activated sludge	P(3HB) produced at Tsinghua University, Beijing, PR China	Immersed in reactors filled with nutrient-depleted activated sludge at RT	Thin solvent-cast films, 100 µm thickness	Mass loss determination	20% mass loss after 18 days (reference material Ecoflex: 5% mass loss after 18 days)	Wang et al. (2004)
P(4HB)	Soil	**No literature data available!**					
P(4HB)	Fresh water	**No literature data available!**					
P(4HB)	Marine	**No literature data available!**					
P(4HB)	Anaerobic	**No literature data available!**					
P(4HB)	Compost	**No literature data available!**					
P(4HB)	Sludge	**No literature data available!**					
P(3HB-*co*-3HV)	Soil	Imperial Chemical Industries (ICI), UK P(3HB-*co*-10%-3HV)	Sandy soil, clay soil, loamy soil, hardwood forest soil, and pinewood forest soil; 15, 28 and 40 °C, water content of soil 12–44% RH, constant pH-value	Injection-molded, dumbbell-shaped tensile-test pieces (83 mm × 2 mm, mass 1.75 g)	Mass loss determination	At 15°C: daily mass loss of P(3HB) amounted to about 0.05, 0.03, 0.06, 0.12, and 0.10% in hardwood-, sandy-, pinewood-, clay-, and loamy soil, respectively	Mergaert et al. (1993)
						At 28 °C: 0.12, 0.06, 0.12, 0.10, and 0.04 for the same soil qualities	
						At 40 °C: 0.14, 0.29, 0.21, 0.48, and 0.46%	
P(3HB-*co*-3HV)	Soil	3HV content 12 to 72 mol-%	Mixture of 90% of a natural soil and 10% of organic compost; 25 °C, 65% RH	Solvent-casted films of P(3HB-*co*-3HV), thickness 0.06 mm	CO_2_ evolution analysis according to ASTM D 5988-03	10–15 weeks: more than 40% degradation According to modelling data: 50% degradation to be reached between 3.3 and 4.4 months, min. 60% degradation after 6 months, about 80% after 9 months, and 90% between 3.3 and 4.4 months	Arcos-Hernandez et al. (2012)
P(3HB-*co*-3HV)	Soil	Goodfellow, USA P(3HB-*co*-8%-3HV)	Soil with 40 and 100% relative humidity, RH	Films, 10 µm thickness	Observation of changes in appearance, chemical signatures, mechanical properties, and molecular mass	Complete degradation after 2 weeks in soil with 100% humidity; significant reduction of mechanical properties after 3 days 40% humidity: no excessive changes after 6 weeks	Kim et al. (2023)
P(3HB-*co*-3HV)	Soil	*Wautersia eutropha* B5786, Russian Academy of Sciences(3HV content not reported)	Soil at two different climate test stations in Vietnam; depth 15 cm	Solvent-cast films, about 10 µm thickness and granules	Mass loss and crystallinity determination	After 10 months: Films at more humid test stations degraded by 61%; granules degraded by 35%. After 12 months: films at less humid test stations degraded by 47%; granules degraded by 28%. Daily loss of 0.18 and 0.08% for films and granules, respectively. Films at less humid test stations degraded by 14%; granules degraded by 35%. After 12 months: films at less humid test station degraded by 8%; granules degraded by 28%	Boyandin et al. (2013)
P(3HB-*co*-3HV)	Soil	*C. eutrophus* B10646, Russian Academy of Sciences P(3HB-*co*-12-mol-%-3HV)	“Agro-transformed field soil, village Minino, Krasnoyarsk”; 35 days, 28 °C, 50% humidity	Films (no info in size, thickness and mass)	Mass loss determination	After 21 days: about 70% degradation	Prudnikova et al. (2017)
						After 28 days: more than 80% degradation	
P(3HB-*co*-3HV)	Soil	P(3HB-*co*-6.2-mol%-3HV) (origin not reported)	Garden soil from the campus of UNESP, Rio Claro, Brazil, 35.6% humidity, pH 5.1	Films (thickness 0.1 µm)	Macroscopic observation; FT-IR	Complete degradation after 30 days	Gonçalves et al. (2009)
P(3HB-*co*-3HV)	Soil	P(3HB-*co*-6.2-mol%-3HV) produced by *Halomonas campisalis* (MCB B-1027) in 14 L bioreactor	“Garden soil”; humidity 15%, 20%, 25% and 30%, 28 ± 2 °C	Films (9 × 6 cm, 20 µm thickness)	Mass loss determination	95% mass loss at humidity of 25% and 30% after 8 weeks; 12% and 23.6% mass loss after 8 weeks at humidity of 15% and 20%	Kulkarni et al. (2011)
P(3HB-*co*-3HV)	Soil	P(3HB-*co*-5-mol%-3HV); Polymer Chemistry Laboratory of RIKEN Institute, Japan	“Intermediate mangrove compartment” in George Town, Malaysia, at the estuary of the Pinang River. Degradation on surface and after burial	Solvent-cast films	Microscopic observation of film degradation, mass loss determination	About 50–70% mass loss after 8 weeks for all tested surface conditions. For buried samples, mass loss amounted to 80 to almost 100% after 8 weeks	Sridewi et al. (2006)
P(3HB-*co*-3HV)	Soil	*Cupriavidus eutrophus* B10646, Russian Academy of Sciences. Glucose as carbon source and valerate as 3HV-precursor P(3HB-*co*-12-mol%-3HV)	“Agro-transformed soil”, humus-rich and slightly alkaline, collected in the temperate zone of Siberia	Polymer disks (solvent-casted) 30mm in diameter, 0.035–0.045 mm thickness, and 35 ± 5 mg of mass	Mass loss determination, change of crystallinity and molecular mass	21 °C: about 60% mass loss after 28 days, about 90% mass loss after 35 days 28 °C: about 85% mass loss after 28 days, complete degradation after 35 days Slight increase in degree of crystallinity, decrease in molecular mass and increase of polydispersity	Volova et al. (2017)
P(3HB-*co*-3HV)	Compost	Ningbo Tianan Biomaterials Co. Ltd., PR China P(3HB-*co*-3-mol-%-3HV), P(3HB-*co*-30-mol-%-3HV), and P(3HB-*co*-40-mol-%-3HV)	According to ISO 14855-1:2005; aerated composting vessel at 58 °C ± 2 °C	Solvent-cast films	Macroscopic observation of film degradation, CO_2_ evolution	80.2%, 89.3%, and 90.5% biodegradation after 110 days for P(3HB-*co*-3%-3HV), P(3HB-*co*-30%-3HV), P(3HB-*co*-40%-3HV), respectively (cellulose reference: 83.1%)	Weng et al. (2011)
P(3HB-*co*-3HV)	Fresh water	P(3HB-*co*-10%-3HV) and P(3HB-*co*-20%-3HV)	Natural freshwater ponds	Injection-molded, dumbbell-shaped tensile-test pieces (83 mm × 2 mm, mass 1.75 g)	Mass loss determination	After 356 days: 77% mass loss for P(3HB-*co*-10%-3-HV), complete degradation of P(3HB-*co*-20%-3HV)	Mergaert et al. (1995)
P(3HB-*co*-3HV)	Fresh water	P(3HB-*co*-12-mol-%-3HV) copolyester obtained by Monsanto Co	Pond water at the Hongo campus of University of Tokyo; immersion of samples at 25 °C for 28 days under continuous stirring	Powder, cast film (100 µm thickness), undrawn monofilament fibers (0.5 mm thickness), and fivefold-drawn fibers (0.2 mm thickness)	Biochemical oxygen demand (BOD) and mass loss determination	BOD tests after 28 days: Powder: 20% Cast films: 20% Undrawn fibers: 20% Fivefold-drawn fibers: 7–8% Mass lost results after 28 days: Cast films: more than 90% Undrawn fibers: 30% Fivefold-drawn fibers: 65%	Komiyama et al. (2021)
P(3HB-*co*-3HV)	Marine	P(3HB-*co*-3HV) copolyesters produced by *C. necator* from butyric and valeric acid; 3HV content 4, 9, 13, 14, 15, 21, and 61 mol-%	Marine water at Kanagawa Prefectural Fishery Experiment Station, Jogashima (Japan) in outdoor tank (1.5 m water depth, 22 + 3 °C)	Thin solvent-casted films (thickness 50–150 µm, 5 × 10 cm in size) for copolyesters with 4, 21, and 61 mol-% 3HV, melt-extruded plates for 9, 13, and 15%, and monofilament fibers for 14 mol-% 3HV	Surface erosion, mass loss determination	Surface erosion 13, 22, and 15 µm for films consisting of P(3HB-*co*-4-mol-%-3HV), P(3HB-*co*-21-mol-%-3HV), and P(3HB-*co*-61-mol-%-3HV), respectively, at (22 + 3 °C) after 3 weeks Surface erosion of 130, 140, and 100 µm for P(3HB-*co*-9-mol-%-3HV), P(3HB-*co*-13-mol-%-3HV), and P(3HB-*co*-15-mol-%-3HV), respectively, after 17 weeks at 21—+ 6 °C for melt-extruded plates Monofilament P(3HB-*co*-14-mol-%-3HV) fiber: 25% mass loss after four weeks, 65% after eight weeks (21 + 6 °C)	Doi et al. (1992a, b)
P(3HB-*co*-3HV)	Marine	P(3HB-*co*-10%-3HV) and P(3HB-*co*-20%-3HV)	Marine environment in Zeebrugge harbor	Injection-molded, dumbbell-shaped tensile-test pieces (83 mm × 2 mm, mass 1.75 g)	Mass loss determination	49–52% mass loss within 270 days	Mergaert et al. (1995)
P(3HB-*co*-3HV)	Marine	Metabolix P(3HB-*co*-3HV) samples (5, 8, and 12% 3HV)	Dynamic incubation in seawater with and without addition of marine sediment. Parallel studies under static conditions	Screw-extruded films 1.27 × 1.27 cm in size	CO_2_ evolution according to according to ASTM 6691	Dynamic conditions: with marine sediment: about 80% mineralization of all samples (5, 8, and 12% 3HV) after 3 weeks, almost complete mineralization after 100 days Static conditions: almost complete mass loss after 49 days with marine sediment, almost 90% without sediment	Thellen et al. (2008)
P(3HB-*co*-3HV)	Marine	P(3HB-*co*-12-mol-%-3HV) copolyester obtained by Monsanto Co	Seawater from Tokyo Bay; 25 °C, continuous stirring	Undrawn monofilament fibers (0.5 mm thickness)	Biochemical oxygen demand (BOD) and mass loss determination	BOD tests after 28 days: 25% degradation Mass lost results after 28 days: 90%	Komiyama et al. (2021)
P(3HB-*co*-3HV)	Marine	P(3HB-*co*-6%-3HV) and P(3HB-*co*-11%-3HV) produced by *Halomonas* sp. SF2003 on glucose plus valeric acid	Marine water (Northwest Atlantic) and microbial consortium previously detached from PHA samples incubated in sea water	Solvent-casted P(3HB-*co*-3HV) disks of 6 mm^2^ diameter	Oxygen consumption	4.4 × 10^−3^ µmol(O_2_) and 3.4 × 10^−3^ µmol(O_2_) per mm^2^ P(3HB-*co*-3HV) surface for P(3HB-*co*-6%-3HV) and P(3HB-*co*-11%-3HV), respectively, after 2 months	Derippe et al. (2024)
P(3HB-*co*-3HV)	Marine	P(3HB-*co*-8-mol-%-3HV) [ENMAT Y1000P PHA (Tianan Biological Materials Co. Ltd., PR China)]	a) Immersion in natural sea water in in Lorient harbor, France, 180 days b) Biodegradation in solid inoculum with foreshore sand, in a solid–liquid inoculum with sand and seawater and in a liquid inoculum with seawater	Extruded films, 200 × 120mm, 200 µm thickness	a) Mass loss determination, surface erosion observation b) CO_2_ evolution	a) after 120 days: 11% mass loss; after 180 days: 36%; complete disintegration after 9 months b) Foreshore sand: 80% biodegradation after 600 days Solid/liquid medium containing foreshore sand and seawater: 90% biodegradation after 210 days Seawater inoculum with two concentrations of biofilm (5 and 50%): 97% biodegradation after 200 days in 5% inoculum, 90% after 300 days in 50% inoculum	Deroiné et al. (2015)
P(3HB-*co*-3HV)	Marine	P(3HB-*co*-3-mol-%-3HV) (ENMAT Y1000P PHA, Tianan Biological Materials Co. Ltd., PR China)	NF EN ISO 19679 test method (aerobic biodegradation of non-floating plastics at a seawater/sediment interface); 25 °C	Microbeads obtained by an emulsion-evaporation process (diameter 50 to 100 µm)	CO_2_ evolution	90% after 250 days	Volant et al. (2022)
P(3HB-*co*-3HV)	Marine	P(3HB-*co*-8-mol-%-3HV) from ICI	Bathyal seafloor off Misaki port in the northern Pacific Ocean, Japan. 757 m depth	Uniform spherical microbeads 50–150 µm in size, prepared via melt homogenization	Mass loss determination	44% mass loss after 5 months in deep sea	Hyodo et al. (2024)
P(3HB-*co*-3HV)	Marine	P(3HB-*co*-8-mol-%-3HV) from ICI	Seawater and soil samples collected from Tokyo Bay. 5 L of seawater mixed with 1 kg of soil; 27 °C	Uniform spherical microbeads 50–150 µm in size, prepared via melt homogenization	BOD	74% degradation after 25 days (reference cellulose: 77%)	Hyodo et al. (2024)
P(3HB-*co*-4HB)	Soil	Green Biological Material Co. Ltd. from Tianjin, PR China	Experimental soil pond in Beijing, plants growing on the surface	Extrusion-casted thin films (no info in size, thickness and mass)	Visual observation and photographic documentation	Films buried in 20 cm depth (aerobic conditions): almost complete degradation of P(3HB-*co*-10%-4HB) films after 2 months, complete degradation after 3 months Films buried in 40 cm depth (anaerobic conditions): Films start to disintegrate after 1 month; complete disappearance after 3 months	Weng et al. (2013)
P(3HB-*co*-4HB)	Soil	Green Biological Material Co. Ltd. from Tianjin, PR China	Garden soil, 60 days, room temperature, 20% water content	Solvent-cast films	Mass loss and molecular mass changes	54, 70, 80, 93, and 82% after 60 days at 5, 7, 10, 15 and 20 mol-% 4HB	Wen and Lu (2012)
P(3HB-*co*-4HB)	Soil	*Cupriavidus eutrophus* B10646, Russian Academy of Sciences. 10% 4HB	“Agro-transformed field soil, village Minino, Krasnoyarsk”; 21 days, 28 °C, 50% humidity	Films (no info in size, thickness and mass)	Mass loss determination	Complete degradation after 28 days, about 97% after 21 days	Prudnikova et al. (2017)
P(3HB-*co*-4HB)	Compost	Ningbo Tianan Biomaterials Co. Ltd., PR China P(3HB-*co*-10-mol-%-4HB)	According to ISO 14855-1:2005; aerated composting vessel at 58 °C ± 2 °C	Solvent-cast films	Macroscopic observation of film degradation, CO_2_ evolution	90.3% biodegradation after 110 days (cellulose reference: 83.1%)	Weng et al. (2011)
P(3HB-*co*-4HB)	Sludge	*C. necator* H16	Incubation in activated sludge from a sewage treatment plant at Tokyo Institute of Technology, Japan, under aeration at 30 °C	Solvent-cast films (0.7 mm thickness)	Macroscopic observation of samples	P(3HB-*co*-10%-4HB) films almost completely decomposed after 2 weeks; completely disappeared after 5 weeks	Doi et al. (1990)
P(3HB-*co*-4HB)	Soil	Mirel™ (Telles) (P(3HB*-co-*4HB); 4HB fraction not reported)	Soil from experimental field of the Agricultural University of Athens (AUA) in Spata (clay loam, pH-value 8.2)	Films mimicking agricultural mulching films	Macroscopic observation of samples and photographic documentation	After 1–2 months: only a few tiny film fragments visible (about 1–2% of original surface area). After three months: complete disappearance of films	Rudnik and Briassoulis (2011)
P(3HB-*co*-4HB)	Soil	*Cupriavidus eutrophus* B10646, Russian Academy of Sciences. Glucose as carbon source, γ-butyrolactone as 4HB-precursor P(3HB-*co*-10-mol-%-4HB)	“Agro-transformed soil”, humus-rich and slightly alkaline, collected in the temperate zone of Siberia	Polymer disks (solvent-casted) 30 mm in diameter, 0.035–0.045 mm thickness, and 35 ± 5 mg of mass	Mass loss determination, change of crystallinity and molecular mass	21 °C: about 15% mass loss after 7 days, about 80% mass loss after 21 days 28 °C: 30% mass loss after 7 days, complete degradation after 21 days Drastic increase of degree of crystallinity, significant decrease of molecular mass and strong increase of polydispersity	Volova et al. (2017)
P(3HB-*co*-4HB)	Soil	Prepared at Universiti Sains Malaysia	Garden of Biological School of University Sains, Malaysia	Solvent-casted P(3HB-*co*-4HB) films (1.2 × 1.2 cm, strongly varying thickness from 0.3 to 6 mm, average mass 0.02 g)	Mass loss determination	After 5 weeks: degradation of about 35.1, 84.2, and 98.8% of the P(3HB-*co*-14-mol-%-4HB), P(3HB-*co*-47-mol-%-4HB), and P(3HB-*co*-87-mol-%-4HB) films	Vigneswari et al. (2019)
P(3HB-*co*-4HB)	Fresh water	P(3HB-*co*-16%-4HB) provided by Mitsubishi Gas Chemical Co., Inc., Japan	Freshwater from Sanshiro Pond at University of Tokyo; open containers, 28 days, 25 °C	Fibers of high tensile strength and elasticity produced by melt spinning	Mass loss determination	Seven days: no mass loss 14 days: about 20% mass loss 21 days: about 40% mass loss 28 days: complete degradation	Omura et al. (2021)
P(3HB-*co*-4HB)	Fresh water	Mitsubishi Gas Chemical Co., Inc., Japan	Freshwater from Sanshiro Pond at University of Tokyo; open containers, 28 days, 25 °C, continuous stirring	Fibers of high tensile strength (> 200 MPa) and elasticity (elongation at break of ∼200%), obtained via melt-spinning	Mass loss determination	After 2 weeks: about 20% mass loss After 3 weeks: about 40%. After 4 weeks: complete degradation	Omura et al. (2021)
P(3HB-*co*-4HB)	Fresh water	Prepared at Universiti Sains Malaysia	Lake near the School of University Sains, Malaysia	Solvent-casted P(3HB-*co*-4HB) films (1.2 × 1.2 cm, strongly varying thickness from 0.3 to 6 mm, average mass 0.02 g)	Mass loss determination	After 5 weeks: degradation of about 45, 57, and 82% of the P(3HB-*co*-14-mol-%-4HB), P(3HB-*co*-47-mol-%-4HB), and P(3HB-*co*-87-mol-%-4HB) films	Vigneswari et al. (2019)
P(3HB-*co*-4HB)	Marine	P(3HB-*co*-4HB) copolyesters produced by *C. necator* from butyric acid and GBL; 4HB content 6 and 10 mol-%	Marine water at Kanagawa Prefectural Fishery Experiment Station, Jogashima (Japan) in outdoor tank (1.5 m water depth, 22 + 3 °C)	Thin solvent-casted films (thickness 50–150 µm, 5 × 10 cm in size) for both copolyesters	Surface erosion	Surface erosion 31 and 33 µm for P(3HB-*co*-6-mol-%-3HV) and P(3HB-*co*-10-mol-%-3HV) films, respectively, at 14 °C after eight weeks; 55 and 60 µm, respectively, after 8 weeks, at 24 °C	Doi et al. (1992a, b)
P(3HB-*co*-4HB)	Marine	P(3HB-*co*-16%-4HB) provided by Mitsubishi Gas Chemical Co., Inc., Japan	Seawater from Tokyo Bay; open containers, 28 days, 25 °C	Fibers of high tensile strength and elasticity produced by melt spinning	Mass loss determination	Seven days: about 65% mass loss 14 days: about 85% mass loss 21 days: about 95% mass loss 28 days: complete degradation	Omura et al. (2021)
P(3HB-*co*-4HB)	Marine	P(3HB-*co*-8.9-mol-%-4HB) from Mirel	Bathyal seafloor off Misaki port in the northern Pacific Ocean, Japan. 757 m depth	Uniform spherical microbeads 50–150 µm in size, prepared via melt homogenization	Mass loss determination	52% mass loss after 5 months in deep sea	Hyodo et al. (2024)
P(3HB-*co*-4HB)	Marine	P(3HB-*co*-8.9-mol-%-4HB) from Mirel	Seawater and soil samples collected from Tokyo Bay. 5 L of seawater mixed with 1 kg of soil; 27 °C	Uniform spherical microbeads 50–150 µm in size, prepared via melt homogenization	BOD	83% degradation after 25 days (reference cellulose: 77%)	Hyodo et al. (2024)
P(3HB-*co*-3HHx)	Soil	*C. eutrophus* B10646, Russian Academy of Sciences. 12% 3HHx	“Agro-transformed field soil, village Minino, Krasnoyarsk”; 28 days, 28 °C, 50% humidity	Films (no info in size, thickness and mass)	Mass loss determination	After 21 days: about 80% mass loss After 28 days: about 90% mass loss	Prudnikova et al. (2017)
P(3HB-*co*-3HHx)	Soil	Genetically modified *C. necator* (Re2058/pCB113)	Composted soil from a farm of Chubu University, Japan. 28 days, 34 °C, 90% humidity, pH-value 5.3	Solvent-casted thin films (0.3 mm thickness, 1.5 cm x 1.5 cm, mass about 20 mg)	Mass loss determination	92.6% mass loss after 28 days of burial for P(3HB-*co*-6%-3HHx) films 98.1% mass loss after 28 days of burial for P(3HB-*co*-17%-3HHx) films	Baidurah et al. (2019)
P(3HB-*co*-3HHx)	Soil	P(3HB-*co*-5-mol%-3HHx); *C. necator* PHB^−^4/pBBREE32d13	“Intermediate mangrove compartment” in George Town, Malaysia, at the estuary of the Pinang River. Degradation on surface and after burial	Solvent-cast films	Microscopic observation of film degradation, mass loss determination	About 50–70% mass loss after eight weeks for all tested surface conditions. For buried samples, mass loss amounted to 80 to almost 100% after 8 weeks	Sridewi et al. (2006)
P(3HB-*co*-3HHx)	Soil	*Cupriavidus eutrophus* B10646, Russian Academy of Sciences. Glucose as carbon source, hexanoate as 3HHx-precursor P(3HB-*co*-12-mol-%-3HHx)	“Agro-transformed soil”, humus-rich and slightly alkaline, collected in the temperate zone of Siberia	Polymer disks (solvent-casted) 30 mm in diameter, 0.035–0.045 mm thickness, and 35 ± 5 mg of mass	Mass loss determination, change of crystallinity and molecular mass	21 °C: about 70% mass loss after 28 days, more than 90% after 35 days 28 °C: more than 90% mass loss after 28 days, complete degradation after 35 days Significant increase of degree of crystallinity, slight decrease of molecular mass, almost constant polydispersity	Volova et al. (2017)
P(3HB-*co*-3HHx)	Fresh water	P(3HB-*co*-3HHx), Kaneka Corporation, Hyogo, Japan. 3HHx content and production strain not disclosed	Fresh water mixed with 0.5 g L^–1^ NH_4_Cl as nitrogen source and 0.1 g L^–1^ KH_2_PO_4_ as phosphate source; 30 °C, 2 weeks	Films prepared by T die cast extrusion; thickness of 100 μm and a size of 1 × 1.5 cm	Isolation of microbes present in biofilms formed during incubation; testing of isolated microbes in liquid media and on solid agar containing P(3HB-*co*-3HHx) as sole carbon source	No quantitative data; observation of clearing zones on agar and change of film properties (transparency, formation of cracks and rough surfaces)	Morohoshi et al. (2018)
P(3HB-*co*-3HHx)	Marine	Kaneka PHBH; 11 mol-% 3HHx	Sea water from Osaka-Nanko Bay area, Japan, 27 °C	Powder	O_2_ consumption	55% after 28 days	Sashiwa et al. (2018)
P(3HB-*co*-3HHx)	Marine	Danimer Scientific (Nodax)	Sea water from the coast of Georgia, USA	Flakes	CO_2_ evolution	83% after 6 months for poly(3HB-*co*-6.5%-3HHx)	Wang et al. (2018)
P(3HB-*co*-3HHx)	Marine	P(3HB-*co*-6-mol-%-3HHx) and P(3HB-*co*-11-mol-%-3HHx) (Aonilex X131A and Aonilex X151A, respectively. Kaneka Corporation, Japan)	NF EN ISO 19679 test method (aerobic biodegradation of non-floating plastics at a seawater/sediment interface), 25 °C	Microbeads obtained by an emulsion-evaporation process (diameter 50 to 100 µm)	CO_2_ evolution	62% and 80% after 250 days for P(3HB-*co*-6-mol-%-3HHx) and P(3HB-*co*-11-mol-%-3HHx), respectively	Volant et al. (2022)
P(3HB-*co*-3HHx)	Marine	P(3HB-*co*-6-mol-%-3HHx) from Kaneka	Bathyal seafloor off Misaki port in the northern Pacific Ocean, Japan. 757 m depth	Uniform spherical microbeads 50–150 µm in size, prepared via melt homogenization	Mass loss determination	20% mass loss after 5 months in deep sea	Hyodo et al. (2024)
P(3HB-*co*-3HHx)	Marine	P(3HB-*co*-6-mol-%-3HHx) from Mirel	Seawater and soil samples collected from Tokyo Bay. 5 L of seawater mixed with 1 kg of soil; 27 °C	Uniform spherical microbeads 50–150 µm in size, prepared via melt homogenization	BOD	68% degradation after 25 days (reference material cellulose: 77%)	Hyodo et al. (2024)
P(3HB-*co*-3HHx)	Marine	P(3HB-*co*-9-mol-%-3HHx) (Kaneka Corporation)	Five different deep-sea floor locations in Pacific Ocean: three bathyal sites [off Misaki Port (depth 757 m), off Hatsushima Island (855 m), and Myojin Knoll (1292 m)], and two abyssal sites [Kuroshio Extension Observatory (5503 m) and Minamitorishima Island (5552 m)]	Injection molded films (1 cm × 3 cm × 0.4 cm) and melt-pressed films (4 cm × 4 cm × 300 μm)	Mass loss, reduction in film thickness, and surface morphological changes	After 1 year: thickness decreased by ~ 70 μm at the shore, ~ 110 μm off Hatsushima Island (757 m), and ~ 10 μm at Minamitorishima Island (1292 m) (original thickness: 4000 µm) At Hatsushima Island sampling site (855 m): mass loss of 22% in 3 months and 52% in 8 months. Film thickness minus 35 μm for 3 months and 70 μm over 8 months (initially 225 μm)	Omura et al. (2024)
P(3HB-*co*-3HHx)	Anaerobic	Procter & Gamble	Anaerobic environment containing anaerobic biosolids from an anaerobic digester at a wastewater treatment plant; constant temperature of 37 °C	Films (0.3 mm thickness)	Mass loss, biogas formation	28% mass loss after 7 days of anaerobic incubation for P(3HB-*co*-3.8%-3HHx) films, complete mass loss after 12 days 80% mass loss after 7 days of anaerobic incubation for P(3HB-*co*-10%-3HHx) films, complete mass loss after 10 days	Morse et al. (2011)
P(3HB-*co*-3HHx)	Anaerobic	Danimer Scientific (Nodax)	Inoculum from an anerobic digester of a wastewater treatment plant, containing sludge and some lipids; constant temperature of 38 °C	Sheets and flakes	Biogas formation	About 55% of carbon of P(3HB-*co*-6.5%-3HHx) flakes and 55% of carbon of P(3HB-*co*-7.1%-3HHx) sheets converted to biogas after 40–60 days of incubation	Wang et al. (2018)
P(3HB-*co*-3HHx)	Activated sludge	RWDC Industries	Sludge from an operational wastewater treatment plant (WWTP) in an aerobic aeration basin	Microbeads, epoxy-resin bound microbeads, and films	CO_2_ evolution	Carbon mineralization of 90, 89, 95, and 8% for epoxy-resin bound P(3HB-*co*-3HHx) microbeads, free P(3HB-*co*-3HHx) microbeads, P(3HB-*co*-3HHx) films, and PLA films, respectively (cellulose as control: 100%). Biodegradation rate: 32-, 30-, 18-, and 19-mL CO_2_·g^–1^·day^–1^ for cellulose, P(3HB-*co*-3HHx) films, free P(3HB-*co*-3HHx) microbeads, and epoxy-resin bound P(3HB-*co*-3HHx) microbeads, respectively	White et al. (2021)
P(3HB-*co*-3HHx)	Activated sludge	P(3HB-*co*-12%-3HHx) from Biotech Company, Guangdong, PR China	Immersed in reactors filled with nutrient-depleted activated sludge at RT	Thin solvent-cast films, 100 µm thickness	Mass loss determination	40% mass loss after 18 days (reference material Ecoflex: 5% mass loss after 18 days; P(3HB): 20%)	Wang et al. (2004)
Different types of mcl-PHA	Soil	P(6.9-mol-%-3HHx-*co*-58.4-mol-%-3HO-*co*-26.7-mol-%-3HD-*co*-6.5-mol-%-3HDD-*co*-1.0-mol-%-3HTD-*co*-0.5-mol-%-3HHxD) from *Pseudomonas putida* PGA1 on saponified palm kernel oil produced in two-stage process	Acidic forest soil well-shaded under fallen decomposing leaves (FS), unshaded alkaline forest soil along of a freshwater stream (FSst), and brackish mangrove soil (MS); Malaysia Films buried in 2 cam depth	Solvent-cast films	Mass loss determination, observation of surface erosion, molecular mass determination, thermophysical data, monomeric composition	After 112 days in soil: 16.7% in FS, 3.0% in FSst, and 4.5% in MS (Parallel test with P(3HB): 4.6% in FS, 73.5% in FSst, and 100% in MS)	Lim et al. (2005)
Different types of mcl-PHA	Soil	P(3HO) Bioplastech Ltd., Dublin, Ireland	Soil consisting of 1/3 field soil and 2/3 forest soil	Melt-processed samples produced via compression-molding to 20 × 20 × 0.2 rectangular specimens	According to ISO 17556; determination of the amount of CO_2_ evolved by CO_2_ absorption in KOH solution	6% after 2 years; 90% for blend P(3HO)/P(3HB) (15/85)	Narancic et al. (2018)
Different types of mcl-PHA	Fresh water	P(54-mol-%-3HO-*co*-23-mol-%-3HD-*co*-15-mol-%-3HDD-*co*-8-mol-%-3HTD) from *P. putida* PGA1 on saponified palm kernel oil	Water from the Kayu Ara River, Malaysia. pH-value 7.47, 28 °C. Long term experiments for 86 days with stirring and aeration with CO_2_-free air. Short term experiments (28 days) with sterilized and non-sterilized water, with and without stirring	Solvent-cast films (15 × 15 mm, thickness not reported)	CO_2_ evolution according to adapted ASTM D5209-01 standard test method Mass loss determination Surface morphology observation Determination of change of monomer contents	71.3% mass loss after 86 days (100% for P(3HB) films tested in parallel) CO_2_ evolution: 133.76 mg per film after 70 days, 115.72 for control (no PHA added)	Ho et al. (2002)
Different types of mcl-PHA	Fresh water	P(3HO) Bioplastech Ltd., Dublin, Ireland	Defined aqueous medium at 21 °C with microbes from activated sludge	Melt-processed samples produced via compression-molding to 20 × 20 × 0.2 rectangular specimens	According to ISO 14851. Determination of the amount of CO_2_ evolved by CO_2_ absorption in KOH solution	About 50% after 56 days; 70% for blend P(3HO)/P(3HB) (15/85) after 56 days	Narancic et al. (2018)
Different types of mcl-PHA	Marine	P(5.5%-3HHx-*co*-89%-3HO-*co*-5.5%-3HD) (“PHO”), P(14%-3HHp-*co*-4%-3HO-*co*-58.1%-3HN-*co*-24%-3HD) (“PHN”), and P(23%-3HHp-*co*-74%-3HN-*co*-2%-3HD) (“PHNac produced by *Pseudomonas putida* KT2440 on fatty acids	Marine water (Northwest Atlantic) and microbial consortium previously detached from PHA samples incubated in sea water	Solvent-casted *mcl*-PHA disks of 6 mm^2^ diameter	Oxygen consumption	0.18 × 10^−3^, 0.70 × 10^−3^, and 0.31 × 10^−3^ µmol(O_2_) per mm^2^ *mcl*-PHA film for “PHO”, “PHN”, and “PHNac”, respectively, after 2 months	Derippe et al. (2024)
Different types of mcl-PHA	Marine	P(3HO) Bioplastech Ltd., Dublin, Ireland	Incubation in seawater from Belgium at 30 °C	Melt-processed samples produced via compression-molding to 20 × 20 × 0.2 rectangular specimens	According to ASTM D6691; determination of the amount of CO_2_ evolved by CO_2_ absorption in KOH solution	40% after 56 days; complete degradation observed after 56 days for blend P(3HO)/P(3HB) (15/85)	Narancic et al. (2018)
Different types of mcl-PHA	Anaerobic	P(3HB-*co*-10-mol%-3HO) (^14^C-radio-labeled and chemically synthesized)	Digester sludge, septage sediments, and landfill reactors	Polymer precipitates from chloroform solution	Evolution of ^14^CO_2_ and ^14^CH_4_	More than 50% mineralization within less than 30 days under all anaerobic degradation conditions, about 90% after 60 days	Federle et al. (2002)

### PHA biodegradation in soil

**Fig. 6:**
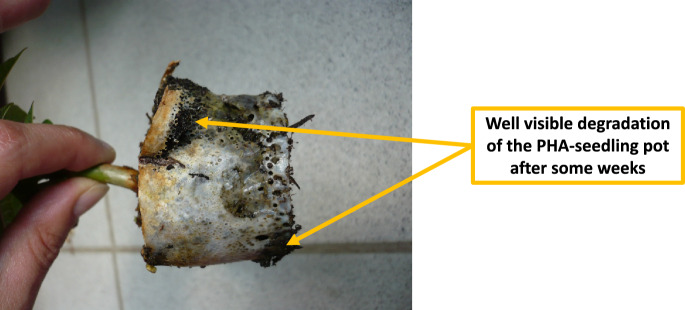
Degradation of a solvent-cast PHA seedling pot when filled with plant soil after a few weeks. Picture: A. Reiterer, TU Graz, 2013

Several factors impact biodegradation of PHA in soil, including environmental conditions (humidity, pH-value, temperature, nutrient availability), material characteristics (composition, crystallinity, shape), and the presence of PHA-degrading microbes (bacteria and fungi), which are all decisive for biodegradation. This complexity of PHA biodegradation in soil was comprehensively summarized in a seminal review article (Fernandes et al. 2020), and only recently substantiated by Prudnikova et al., who monitored different P(3HB) biodegradation rates in soil samples of different microflora, chemical composition, temperature, and humidity (Prudnikova et al. 2024). Figure 6 shows an illustrative example for PHA biodegradation in soil: Small seedling pots were prepared from solvent-casted PHA in the laboratories of one of the authors of this article. After filling with plant soil, planting a flower in it, and irrigation, degradation of the originally intact pot, recognizable by its progressing dissolution, was well visible already after a few weeks.

The following paragraphs provide case studies reported on biodegradation of different types of PHA in soil of different quality.

#### P(3HB) homopolyester in soil

Even the highly crystalline and somewhat recalcitrant homopolyester P(3HB), which constitutes the best studied member of the PHA family, was shown to be biodegradable in soil. This was demonstrated as far back as 1992, when Luzier presented pioneering and seminal data for ICI’s BIOPOL™ P(3HB) film degradation in soil. The homopolyester was ^14^C-labeled, and biodegradation was monitored by measuring radioactive CO_*2*_ gas evolvement. In this study, two different soil qualities were used, and water was added periodically to maintain sufficient humidity. During an 18-week period, 70% of the P(3HB) film degraded in the more microbially active soil, while the same film showed 30–35% less degradation in the inactive soil sample (Luzier 1992). Two decades later, radio-labeled PHA was again used by other authors to monitor mineralization of P(3HB) under anaerobic conditions by release of ^14^CO_2_ and ^14^CH_4_ with similar results (Federle et al. 2002).

Soon after Luzier, Mergaert et al. studied biodegradation of PHA biopolyesters in Belgian soil types of different composition (sandy soil, clay soil, loamy soil, hardwood forest soil, and pinewood forest soil) at 15, 28 and 40 °C, having 12–44% relative humidity (RH), and at constant pH-value. They used P(3HB) from ICI, UK in the form of injection-molded, dumbbell-shaped tensile-test pieces (83 mm × 2 mm, mass 1.75 g). Authors noticed that during degradation in soil, the PHA surfaces became rougher (biodeterioration phase), thus enabling microbial attack, and degradation (mass loss) which was linear over time. Higher temperatures favored degradation rates: At 15 °C, daily mass loss of P(3HB) amounted to about 0.05, 0.02, 0.06, 0.12, and 0.10% in hardwood-, sandy-, pinewood-, clay-, and loamy soil, respectively. At 28 °C, these daily mass loss values increased to 0.12, 0.06, 0.12, 0.10, and 0.04% for the same soil samples. At 40 °C, daily mass loss of 0.14, 0.29, 0.21, 0.48, and 0.46% respectively was observed. Comparison experiments carried out by incubation in sterile phosphate buffer systems did not show any degradation (monitored via mass loss determination) at pH 4 and 7.4, 4 at 15, 28, 40, and 55 °C even after 98 days, although significant molecular mass reduction was observed at higher temperature (55 °C) (Mergaert et al. 1993).

In a later confirmatory study by Kim and colleagues, P(3HB) homopolyester samples obtained from ICI were tested for biodegradability. Here, biodegradability of thin P(3HB) sheets obtained via solvent-casting was monitored at 28, 37, and 60 °C by burying them in forest soil covered with fallen leaves, sandy soil on a riverbank, activated sludge soil located near a wastewater treatment facility, and in farm soil. After an incubation period of 25 days, buried P(3HB) sheets displayed nearly complete degradation (average mass loss of five replicates: 98.9%) in activated sludge soil at 37 °C, while degradation in farm soil resulted in a mass loss of only 68.8% after 25 days at the same temperature. In sandy soil and forest soil, biodegradation was considerably slower, yet still detectable (10% and 7% mass loss after 25 days and 37 °C, respectively). At lower temperature (28 °C), lower mass loss was observed in sandy soil, activated sludge soil, and farm soil (5.8, 68.9, and 41.3%, respectively); only in forest soil, biodegradation slightly increased (10.5% mass loss). At 60 °C, biodegradation drastically decreased due to the deactivation of microbial enzymes at these thermophilic conditions; 4.9, 4.5, 30.5, and 14.8% mass loss were observed for P(3HB) buried in forest-, sandy-, activated sludge-, and sludge soil, respectively. These differences substantiate the significant impact of the specific environmental conditions (temperature, soil composition and microbial community) on biodegradability of PHA. However, biodegradation of P(3HB) indeed occurred in all soil qualities tested at all temperature levels. Noteworthy, P(3HB) biodegradability determined in this study even outperformed results for biodegradability of Novamont’s Mater-Bi, a thermoplastic starch (TPS)-based composite material, which was used as “positive reference” material in form of compression-molded thin films, and which is well known for its rapid disintegration when used as disposable “bioplastic” bag in the organic waste bin. It was remarkable that complete biodegradation of the TPS (Mater-Bi) bags did not occur under any of the tested conditions in soil: 72.1% degradation was determined in activated sludge after 55 days [more than double duration compared to P(3HB)] at 60 °C as the highest degree of degradation (65.0 and 60.0% at 28 °C and 37°C, respectively), while all other temperature/soil combinations gave rather modest mass losses between 12.4% (forest soil at 37 °C) and 27.1% (farm soil at 28 °C). The second reference material tested in this study under the same temperature conditions in the same types of soil was SoGreen, an aliphatic polyester of succinic acid, adipic acid, butanediol and ethylene glycol commercialized as a “biodegradable” material. Like the P(3HB) specimens, it was studied as compression-molded film samples. In activated sludge, biodegradation of SoGreen films at 28 °C, 37 °C, and 60 °C resulted in mass losses of 77.5, 69.1, and 62.8%, respectively, which still is a poor performance compared to the 98.9% mass loss of P(3HB) at 37 °C (Kim et al. 2000).

P(3HB) biodegradability in soil was also established by Prudnikova et al., who tested P(3HB) and related copolyester films (results for copolyesters tested in parallel *vide infra* in individual sections) from *C. eutrophus* strain B10646 in in laboratory-scale soil environment (“Agro-transformed field soil, village Minino, Krasnoyarsk, Russia”) for 35 days, at a temperature of 28 °C, and 50% RH. After only 21 days, 60% of the highly crystalline (78% structural conformity) P(3HB) films were degraded, as determined via mass loss determination; after 35 days, only about 10% of the initial mass remained (Prudnikova et al. 2024).

Monitoring of biodegradation of ICI-P(3HB) powder and thin films in soil from the coastal area near Ravenna, Italy, via CO_2_ evolution was first established by Modelli et al. according to the test method ASTM D 5988-96. Plotting CO_2_ evolution as a function of time, it was shown by the authors that the degradation mechanism is analogous to a classical Michelis–Menten model for enzymatic reactions. Complete degradation of the P(3HB) powder was observed after less than three months. This study once again showed the significance of high surface-to-volume ratio of a polymer to be degraded: While after 60 days at 22 ± 3 °C, about 20 mmol CO_2_ were released from degradation of a 0.5 g P(3HB) sample, about 10% of this CO_2_ quantity evolved from the same mass of the same P(3HB) quality after the same test period when applied as a thin compression-molded film. Moreover, this study confirmed previous assumptions about PHA biocatalytic degradation occurring on the polymer surface (Modelli et al. 1999).

Soil humidity is important for the biodegradation of PHA. This was demonstrated only recently by Kim et al. (2023), who studied P(3HB) biodegradation in soil of two different humidity levels: soil completely water-saturated (100% relative humidity, RH), and soil with 40% RH. P(3HB) samples, prepared as chloroform-cast films with about 15 µm thickness from Goodfellow, USA, degraded completely after two weeks in the 100% RH soil; already after 3 days, significant reductions in mechanical properties were observed by the authors. In contrast, P(3HB) samples exposed to soil with 40% RH did not show excessive changes after six weeks of exposure (Kim et al. 2023).

The impact of different soil conditions on P(3HB) biodegradation was evaluated by Boyandin et al., who tested thin P(3HB) test films obtained via solvent-casting (30 mm diameter, 0.1 mm thickness, about 73 mg mass), in addition to granules obtained by pelletizing P(3HB) powder via cold compaction. Two different climate test stations in Vietnam were selected for the soil biodegradation tests, which lasted 10 and 12 months, respectively. Samples were placed in meshed gauze jackets, and buried in a soil depth of 15 cm. Degradation was monitored in 30 days intervals via mass loss and determination of molecular mass and crystallinity. After 10 months, P(3HB) films buried at the first test station, which experienced more rain fall at the beginning of the study, were almost completely degraded, while pelletized (granular) P(3HB) subjected to the same conditions remained about 45% of its initial mass. The less humid conditions at the second test station resulted in lower degradation: 47 and 28% mass loss were observed for P(3HB) films and pellets, respectively, after 12 months. Expression of the degradation rate as daily mass loss was 0.33% and 0.13% from the two sites, respectively; for granules, these values amounted to 0.18 and 0.08% daily. Notably, the mass of PE films used as negative controls remained unchanged throughout the test period (Boyandin et al. 2013).

Jain and Tiwari (2015) tested biodegradation of solvent-cast P(3HB) films produced by *C. necator* in garden soil during an incubation period of 6 months. Samples were monitored for mass loss in 15-day intervals. P(3HB) films had 64.3% mass loss within 6 months. Interestingly, films consisting of blends of P(3HB) with the compatible thermoplastic material cellulose acetate butyrate at different ratios tested in parallel for biodegradation in garden soil revealed slower biodegradation despite the blends being more amorphous; however, this lower crystallinity was compensated by higher hydrophobicity of the blends than for the pristine P(3HB), which may have made them less susceptible towards microbial attack. Unfortunately, no information about the exact conditions governing the biodegradation process was reported, such as humidity, temperature, or pH-value (Jain and Tiwari 2015).

Metabolix P(3HB) films were studied by Gómez and Michel Jr. (2013) for biodegradability in soil based on ASTM D5988-03 (“Standard test method for determining aerobic biodegradation in soil of plastic materials or residual plastic materials after composting, Standard D5988-03”) determining biodegradation via CO_2_ evolution. Soil used for the study was a mixture of 43% certified organic topsoil, 43% no-till farm soil, and 14% sand. Moisture was adjusted to 60% of moisture hold capacity. After 660 days of incubation, about 70% of the biopolyester samples (injection-molded films with about 0.6 mm thickness) were degraded, a rate similar to parallel setups with the positive control cellulose. No degradation was observed for negative controls PE and PP, which were also buried in soil as part of the same study (Gómez and Michel Jr. 2013).

Vigneswari et al. (2019) studied biodegradation of solvent-cast P(3HB) films (Sigma Aldrich; 1.2 × 1.2 cm, strongly varying thickness from 0.3 to 6 mm, average mass 0.02 g) in the tropical garden of Biological School of University Sains, Malaysia. Samples were placed in meshes, and buried at a depth of 10 cm. After 5 weeks, a P(3HB) mass reduction of about 43% was observed (Vigneswari et al. 2019).

Sridewi et al. (2006) studied biodegradation of P(3HB) films during eight weeks at three different zones in an “intermediate mangrove compartment” in George Town, Malaysia, at the estuary of the Pinang River. P(3HB) was obtained from PHB Industrial S/A, Brazil, and processed to 1 × 1 cm films (thickness not reported) via solvent-casting. The three different test sites differed regarding the period of sea water inundation, frequency of sea water intrusion, and proneness to wave action. The films were either incubated at the surface of sediments or buried in the individual sediments. Biodegradation was monitored via mass loss of sample films. About 50–70% mass loss for P(3HB) films was monitored after eight weeks for all tested surface conditions. For buried samples, mass loss amounted to 80 to almost 100% after the same time. Remarkably, P(3HB) samples containing 38% wt-% TiO_2_ as additive displayed slightly slower biodegradation (Sridewi et al. 2006).

Following the ASTM D5988-18 standard, Quin et al*.* demonstrated that P(3HB) biodegradation in soil (mixture of soil from local forests in Illinois; room temperature, pH-value 7.4) reached more than 80% degradation within 90 days for microbially produced isotactic P(3HB). Under the same conditions, “syndio-rich P(3HB)” (chemically synthesized from cyclic meso-dimethyl diolide; the semicrystalline products display many stereo-defects randomly distributed along the polyester chain) achieved about 54% biodegradation after 90 days. This value amounted to about 70% for synthetic (*R*)-P(3HB), which was obtained by enantioselective ring-opening-polymerization of *rac*-meso-dimethyl diolide. Test setups were designed as follows: 150 g soil plus 41.8 mL water and 0.538 g PHA were mixed per test bottle; microcrystalline cellulose was used as positive reference. Through calculations based on first-order kinetics, authors predicted that “syndio-rich P(3HB)” will reach 90% biodegradation in soil after 268 days, while synthetic (*R*)-P(3HB) will reach this value already after 145 days, and 105 days will suffice for 90% biodegradation of microbial P(3HB) under the given conditions (Quinn et al*.*, 126).

Volova et al*.* compared degradation of the homopolyester P(3HB) and three different PHA copolyesters in laboratory soil microecosystems. Soil samples consisted of “agro-transformed soil”, humus-rich and slightly alkaline, collected in the temperate zone of Siberia. Solvent-casted PHA disks 30 mm in diameter, 0.035–0.045 mm thickness, and 35 ± 5 mg of mass were placed in nylon mesh bags and buried in soil in a depth of 2 cm for 35 days at a temperature of 21–28 °C and 50% moisture content. In seven-days intervals, mass loss and change of molecular mass and crystallinity were monitored. Moreover, microbial soil community attached to the polymer samples was isolated, and specific and common PHA-degrading species on individual polymer samples were identified. P(3HB) biodegradation according to mass loss occurred faster at 28 °C (97% mass loss) than at 21 °C (about 60% mass loss). Interestingly, the degree of crystallinity of P(3HB) samples remained constant for 35 days, indicating a similar degradation of crystalline and amorphous regions under the prevailing soil and microbial conditions. Molecular mass and molecular mass distribution of samples also changed only insignificantly during the first 4 weeks of degradation. Identification of microbial species involved in P(3HB) degradation revealed an abundance of the genera *Mitsuaria*, *Chitinophaga*, and *Acidovorax* (Volova et al. 2017).

A recent study by Prudnikova et al. investigated biodegradation of P(3HB) produced by *C. necator* B-10646 obtained via solvent-casting. Films (thickness 0.035–0.045 mm, mass 35 ± 5 mg, degree of crystallinity 78%) were placed in mesh bags and buried in soil in a depth of 5–10 cm. Two drastically different climatic regions were selected for the study: One location was in Eastern Siberia, where tests were carried out in “chernozem soil” (fertile black soil) under a sharply continental climate during the summer season for three months. The second location was in Kerala, India, where samples were buried for five months under tropical conditions in red ferralitic soil. It was shown that films buried in the Siberian “chernozem soil” degraded faster than in the red Indian soil due to a lower microflora, lower humidity and lower nutrient concentration in the latter. While half of the film mass was lost after 64.8 days in Siberian soil, it took 126.4 days for 50% mass loss under the conditions prevailing in the Indian soil. Determination of crystallinity and molecular mass before and after the degradation tests revealed the priority biodegradation of the amorphous polymer phase by the microflora (Prudnikova et al. 2024).

#### P(3HB) blends in soil

Narancic et al. (2018) studied biodegradation of P(3HB) composites with different other biopolymers, such as PLA, PCL, and PBS. Melt mixing of the polymers mixtures was carried out using a Brabender melt processor. Melt-processed samples were compression-molded to 20 × 20 × 0.2 rectangular specimens. These specimens were subjected to soil biodegradation tests according to ISO 17556. Used soil consisted of 1/3 field soil and 2/3 forest soil. After the test period, P(3HB) blends with P(3-hydroxyoctanoate) (85/15) and PCL (60/40) showed complete soil biodegradability according to the standard, while blend of P(3HB) and PBS (50/50), and P(3HB) and PLA (20/80) did not meet the requirements defined in the standard (about 55% for P(3HB)/PBS, not biodegradation for P(3HB)/PLA). This was expectable due to the negative contribution of PLA and PBS to soil biodegradation: Parallel to blends, also the pristine biopolymers were tested; here, P(3HB) and PCL showed biodegradation in soil according to the norm, in contrast to PLA and PBS.

#### P(3HB-*co*-3HV) copolyesters in soil

In 1993, Mergaert et al. reported on the faster biodegradation of P(3HB-*co*-10%-3HV) copolyesters in soil compared to P(3HB) under identical conditions (15, 28 and 40 °C, water content of soil 12–44% RH, constant pH-value). Different soil qualities were used in this study: sandy soil, clay soil, loamy soil, hardwood forest soil, and pinewood forest soil; tested PHA samples were injection-molded, dumbbell-shaped tensile-test pieces (83 mm × 2 mm, mass 1.75 g); copolyester biodegradation outperformed the rate for P(3HB) (*vide supra*) for all soil qualities and all temperature levels. Also, for P(3HB-*co*-10%-3HV), higher temperature favored higher degradation rates: At 15 °C, daily mass loss of P(3HB-*co*-10%-3HV) amounted to about 0.07, 0.03, 0.10, 0.14, and 0.07% in hardwood-, sandy-, pinewood-, clay-, and loamy soil, respectively. At 28 °C, these daily mass loss values increased to 0.17, 0.05, 0.17, 0.24, and 0.13 for the same soil qualities. At 40 °C, daily mass loss of 0.64, 0.33, 0.36, 0.48, and 0.51% was observed (Mergaert et al. 1993).

Arcos-Hernandez et al. studied soil biodegradation of solvent-cast films of P(3HB-*co*-3HV) copolyesters with 3HV content ranging from 12 to 72 mol-%; average thickness of the films was 0.06 mm. Biodegradation was referenced to cellulose and starch as positive standards and monitored via measuring CO_2_ evolution according to the ASTM method D 5988-03. The soil used for the study was a mixture of 90% of a natural soil and 10% of organic compost. This composition matches the requirements for standardized cellulose degradability according to ASTM D 5988-03. 1 g polymer was added to 400 g of soil mixture and kept in a constant environment (25 °C, 65% RH). After 4 months, physical disintegration, characterized by cracks and holes, was easily visible for all samples. For biodegradation rates, it was shown that values for the first 10–15 weeks were relatively constant for all tested samples; more than 40% (by mass) of all samples were degraded after that time. Using the Hill model, the authors estimated from the generated data that after 6 months, at least 60% of all polymer samples were degraded, and after 9 months all films were anticipated to reach about 80% biodegradation; all samples were expected to reach 90% biodegradation between 10.7 and 22.2 months and 50% between 3.3 and 4.4 months (Arcos-Hernandez et al. 2012).

Humidity also plays a key role during biodegradation in soil for P(3HB-*co*-3HV) copolyesters, like that shown for P(3HB). This was recently shown in experiments comparing 100% and 40% RH performed by Kim et al. (2023) in parallel to their setups studying P(3HB) degradation. For P(3HB-*co*-8%-3HV) copolyester films (10 µm thickness) obtained by Goodfellow, at 40% RH degradation occurred slowly compared to 100% humidity where polymer specimens were completely degraded after two weeks (Kim et al. 2023).

Boyandin et al. (2013) studied P(3HB) and P(3HB-*co*-3HV) biodegradation in the tropical Vietnamese soil in parallel although they did not report 3HV content for the copolyesters. Surprisingly, copolyesters degraded slower than the homopolyester P(3HB) under the same test conditions, which the authors attributed to substrate specificity of extracellular depolymerase enzymes excreted by the microbial community prevailing in the studied soil environments (Boyandin et al. 2013). It should be noted that this specialization of microbes to degrade specific types of PHA was later investigated and confirmed by Prudnikova et al. (2017), who showed that that the dominance of species in a PHA-degrading microbial consortium differs for diverse types of PHA, even when tested in the same environment. 61% of P(3HB-*co*-3HV) films degraded under the more humid conditions at test station 1 within 10 months, which can be compared with 35% mass loss for P(3HB-*co*-3HV) granules tested at the same location. At the less humid test station 2, 14% mass loss was observed for P(3HB-*co*-3HV) films, and only 8% for P(3HB-*co*-3HV) in pelletized form (Boyandin et al. 2013).

P(3HB-*co*-12 mol-%-3HV) copolyester biodegradability in soil was also established by Prudnikova et al. who tested copolyester films from *Cupriavidus eutrophus* strain B10646 in laboratory-scale in soil environment over 28 days, at 28 °C, and 50% relative humidity. After 21 days, the P(3HB-*co*-12 mol-%-3HV) films (crystallinity about 60%) degraded by about 70%, and by more than 80% after 28 days (Prudnikova et al. 2017).

Gonçalves et al. studied biodegradation rates of P(3HB-*co*-6.2 mol%-3HV) films and compared them to films of polypropylene (PP) and PP/P(3HB-*co*-3HV) blend (ratio 4/1) buried in soil over 120 days. Films were prepared via hot pressing, and test specimens of 5 × 5 cm × 0.1 mm were used. Experiments were carried out in boxes containing garden soil (from the campus of UNESP, Rio Claro, Brazil, 35.6% RH, pH-value 5.1), and sampled after 30, 60, 90, and 120 days. After 30 days, P(3HB-*co*-6.2 mol%-3HV) films had completely degraded. PP/P(3HB-*co*-3HV) blend films were microbially attacked, which resulted in a rearrangement of the polymer chains within the PP amorphous phase and interphase of the two polymers. The pure PP films exhibited a type of “chemi-crystallization”, which affected the polymer matrix and the morphology, but films did not biodegrade (Gonçalves et al. 2009).

Kulkarni et al. studied biodegradation of P(3HB-*co*-4 mol%-3HV) films in soil. The biopolyesters were produced by the alkali-tolerant *Halomonas campisalis* (MCB B-1027) and processed into thin (20 µm) solvent cast films 9 × 6 cm in size. After confirming the principal biodegradability of the material according to ASTM D5209-91 (incubation of microbes in a minimal medium with PHA as sole carbon source and monitoring CO_2_ evolution), films were place in plastic containers and buried in garden soil having relative humidity of 15%, 20%, 25% and 30% and incubated at 28 ± 2 °C. Biodegradation was monitored in seven days intervals over two months via mass loss determination. The rate of biodegradation after eight weeks was 12% and 23.6%, respectively, in soil containing 15% and 20% humidity. At a humidity of 25% and 30%, PHA films had disintegrated and possibly degraded as well, into tiny pieces after six weeks, and 95% mass loss was observed after eight weeks (Kulkarni et al. 2011).

In parallel to the experiments with P(3HB) films as has been described by Sridewi et al. and reported here earlier, these authors also tested P(3HB-*co*-5 mol%-3HV) films under the same conditions (surface exposure and soil burial at three different sites). The polymer was provided by the Polymer Chemistry Laboratory of RIKEN Institute, Japan. Under these conditions, degradation rates were very similar to those observed for P(3HB) films (Sridewi et al. 2006).

In addition to homopolyester P(3HB) (*vide supra*), Volova et al. (2017) also compared degradation of the P(3HB-*co*-12-mol-%-3HV) copolyesters (disks 30 mm in diameter, 0.035–0.045 mm thickness, and 35 ± 5 mg of mass, degree of crystallinity 60%) in laboratory soil microecosystems based on soil collected in the temperate zone of Siberia. Similar to P(3HB), biodegradation according to mass loss occurred faster at 28 °C (about 85% mass loss after 28 days, complete degradation after 35 days) than at 21 °C (about 60% mass loss after 28 days, about 90% after 35 days). In contrast to P(3HB), the degree of crystallinity of P(3HB-*co*-12-mol-%-3HV) samples slightly increased during the degradation period, indicating that amorphous regions were degraded at higher rates. Molecular mass decreased, while molecular mass distribution of samples (polydispersity) increased during the first 4 weeks of degradation, clearly showing that polyester chains were depolymerized, causing a growing number of fragments with different degrees of polymerization. Identification of microbial species involved in P(3HB-*co*-12-mol-%-3HV) degradation revealed *Roseomonas massiliae* and *Delftia acidovorans* as the predominant degraders (Volova et al. 2017).

#### P(3HB-*co*-4HB) copolyesters in soil

In 2013, Weng et al. compared the biodegradability of P(3HB-*co*-10%-4HB) copolyester as well as P(3HB-*co*-4HB)/PLA blends with a weight share of 25, 50, and 75% PLA in real soil environments; the blends were produced by melt-extrusion after high-speed pre-mixing. It was shown that unblended, pristine P(3HB-*co*-4HB) degraded faster than the blends: As the PLA fractions increased in the blends biodegradation rate decreased. P(3HB-*co*-10%-4HB) was produced by GreenBio, PR China. Studies were carried out in an experimental soil pond in Beijing; plants were planted in the soil to mimic real environmental conditions. Samples were buried in depths of 20 cm (to mimic aerobic conditions) and 40 cm (to mimic anaerobic conditions). During the 150 days of testing, temperature at a depth of 20 cm fluctuated between 10 and 25 °C. Biodegradation of films was monitored via visual testing and photographic documentation. In 20 cm depth, P(3HB-*co*-10%-4HB) films almost completely degraded after two months, and blends became fragmented to different degrees depending on the P(3HB-*co*-10%-4HB) content. After three months, only PLA fragments were found in the soil, while the P(3HB-*co*-10%-4HB) fraction had completely disappeared. Importantly, authors noted in their article that “*the films were either digested by the microorganisms or broken into small fragments*”, which describes the shortcoming of the used test method: It is not really known whether all of the PLA fraction remined in the soil, whether some had degraded and if there were particles remaining that were too small for the naked eye to observe. A similar trend was observed for samples buried at a depth of 40 cm: the higher the P(3HB-*co*-10%-4HB) content in the blends, the faster the degradation rate. After one month, the pure P(3HB-*co*-10%-4HB) films started to disintegrate, and after three months, they disappeared (Weng et al. 2013).

Wen and Lu studied biodegradation of solvent-cast P(3HB-*co*-4HB) copolyester (GreenBio, PR China) films of 0.1 to 0.3 mm thickness with different 4HB content (5, 7, 10, 15 and 20 mol-%) in garden soil during a period of 60 days at room temperature. Biodegradation was periodically measured by mass loss and molecular mass changes. These authors showed that biodegradation rate of P(3HB-*co*-4HB) copolyesters in soil strongly depends on crystallinity, which in turn was dependent on the 4HB fraction: 54, 70, 80, 93, and 82% mass loss were observed after 28 days for P(3HB-*co*-4HB) copolyesters with 5, 7, 10, 15 and 20 mol-% 4HB, respectively. The higher degradability of P(3HB-*co*-20%-4HB) in comparison to P(3HB-*co*-15%-4HB) was explained by the authors in the following manner: P(3HB-*co*-20%-4HB) being sufficiently flexible to form uniform and compact structures, which in turn generated denser and smoother surfaces which were less prone to microbial attack (Wen and Lu 2012).

Prudnikova et al. (2017) tested P(3HB-*co*-10-mol-%-4HB) soil biodegradability in laboratory-scale soil environment for 28 days, at a temperature of 28 °C, and 50% humidity. After 28 days, the low-crystalline copolyester films (X_C_ = 50%) had completely degraded (97% after 21 days) (Prudnikova et al. 2017).

Vigneswari et al. (2019) studied biodegradation of solvent-cast P(3HB-*co*-4HB) films (prepared at Universiti Sains Malaysia; 1.2 × 1.2 cm, strongly varying thickness from 0.3 to 6 mm, average mass 0.02 g) in the garden of Biological School of University Sains, Malaysia. 4HB contents amounted to 14, 47, and 87 mol-%. Samples were placed in meshes, and buried at a depth of 10 cm. After 5 weeks, about 35.1, 84.2, and 98.8% of the P(3HB-*co*-14-mol-%-4HB), P(3HB-*co*-47-mol-%-4HB), and P(3HB-*co*-87-mol-%-4HB) films, respectively, were degraded (Vigneswari et al. 2019).

During the period June 2008 to January 2009, Rudnik and Briassoulis carried out long-term soil biodegradation studies with films of Mirel™ P(3HB-*co*-4HB) copolyesters, produced by the Metabolix-ADM joint venture Telles. Conditions for the study simulated agricultural mulching films in soil at the end of life. It was shown that microorganisms easily attacked the surface of the PHA films from the very beginning of soil burial exposure, independent of fluctuating environmental temperature conditions. After 1 to 2 months, only a few tiny fragments of the films were still visible, amounting to about 1–2% of the original film surface area. After three months, no P(3HB-*co*-4HB) films were visible anymore. PLA films tested in parallel showed significantly slower degradation; the first visual changes of PLA films were seen only after 4 to 5 months, when cracks started to appear. Polyethylene (PE) films were used as the negative control, for which no degradation was observed during this period. In contrast, cellulose, used as positive reference, had disappeared after one month of soil exposure. The progress of degradation was documented by the authors by a comprehensive compilation of photographs (Rudnik and Briassoulis 2011).

In addition to P(3HB) and P(3HB-*co*-3HV) (*vide supra*), Volova et al. (2017) also compared degradation of the P(3HB-*co*-10-mol-%-4HB) copolyesters (disks 30 mm in diameter, 0.035–0.045 mm thickness, and 35 ± 5 mg of mass, degree of crystallinity 50%) in laboratory soil microecosystems based on soil collected in the temperate zone of Siberia. Like P(3HB) and P(3HB-*co*-3HV), P(3HB-*co*-10-mol-%-4HB) biodegradation monitored via mass loss also occurred faster at 28 °C than at 21 °C. Already after seven days, 30% mass loss was determined at 28 °C; after 21 days, the samples already completely vanished. At 21 °C, mass loss amounted to about 15% after 7 days, almost 80% after 21 days, while complete degradation was monitored after 35 days. The degree of crystallinity of P(3HB-*co*-10-mol-%-4HB) drastically increased during the degradation period, indicating that rapid degradation of amorphous regions: at 21 °C, degree of crystallinity increased from 50 to 63%, and to 61% at 28 °C. Molecular mass of P(3HB-*co*-10-mol-%-4HB) significantly decreased, while and molecular mass distribution of samples increased already during the first week of incubation in soil. *Roseateles depolymerans*, *Streptomyces gardneri*, and *Cupriavidus* sp. were identified as specific degraders of P(3HB-*co*-10-mol-%-4HB) (Volova et al. 2017).

#### P(3HB-*co*-3HHx) copolyesters in soil

Biodegradability of *C. eutrophus* B10646 P(3HB-*co*-12 mol-%-3HHx) films in soil was tested at a temperature of 28 °C, and 50% humidity. After 21 and 28 days, a mass loss of more than 80 and more than 90%, respectively, of the original copolyester films (X_C_ = 56%) was observed (Prudnikova et al. 2017).

Baidurah et al. (2019) reported that during P(3HB-*co*-3HHx) films biodegradation in soil, 3HHx moieties in polyester chains were preferentially biodegraded due to low crystallinity, as shown by the authors via thermally assisted hydrolysis and methylation-gas chromatography (THM-GC), which allowed for determination of the monomer composition of P(3HB-*co*-3HHx) during soil burial studies. While the fraction of 3HB monomers in P(3HB-*co*-3HHx) increased during degradation, the content of 3HHx steadily decreased coinciding with the loss of mass loss of the entire test piece. The P(3HB-*co*-3HHx) samples were produced by genetically modified *C. necator*, and processed to thin films (0.3 mm thickness, 1.5 × 1.5 cm, mass about 20 mg) via solvent casting. Soil burial tests were carried out in an incubator at a RH of about 90%, a constant temperature of 34 °C, and a pH-value of 5.3. Composted soil from a farm of Chubu University, Japan, was used for the tests. After 28 days of incubation, about 7.4% of the mass of P(3HB-*co*-6%-3HHx) films remained; for P(3HB-*co*-17%-3HHx) films, only about 1.9% of the mass was left over after the same time (Baidurah et al. 2019).

In addition to P(3HB) and P(3HB-*co*-3HV), Sridewi et al. (2006) has also reported on P(3HB-*co*-5 mol%-3HHx) films under the same conditions (surface exposure and soil burial at three different sites). These copolyesters were produced by the strain *C. necator* PHB^−^4/pBBREE32d13, with reported degradation rates for P(3HB-*co*-5 mol%-3HHx) films similar to those observed for P(3HB) and P(3HB-*co*-3HV) films (Sridewi et al. 2006).

In addition to P(3HB) and P(3HB-*co*-3HV) and P(3HB-*co*-4HB) (*vide supra*), Volova et al. (2017) also compared degradation of the P(3HB-*co*-12-mol-%-3HHx) copolyesters (disks 30 mm in diameter, 0.035–0.045 mm thickness, and 35 ± 5 mg of mass, degree of crystallinity 56%) in laboratory soil microecosystems based on soil collected in the temperate zone of Siberia. Again, P(3HB-*co*-12-mol-%-3HHx) biodegradation according to mass loss occurred faster at 28 °C (more than 90% mass loss after 28 days, complete degradation after 35 days) than at 21 °C (about 70% mass loss after 28 days, more than 90% after 35 days). Similar as for P(3HB-*co*-3HV) and P(3HB-*co*-4HB) samples, the degree of crystallinity of P(3HB-*co*-12-mol-%-3HHx) samples increased during 28 days from 56 to 60%, indicating that amorphous regions were degraded at higher rates. This increase was less pronounced as for P(3HB-*co*-4HB) samples, but more than for P(3HB-*co*-3HV) samples tested in parallel (from 50 to 63% and from 60 to 64%, respectively). Molecular mass slightly decreased for 28 days, with the molecular mass distribution (polydispersity) of samples remaining almost constant. Identification of microbial species involved in P(3HB-*co*-12-mol-%-3HHx) degradation revealed the species *Pseudoxanthomonas* sp., *Pseudomonas fluorescens*, *Ensifer adhaerens*, and *Bacillus pumilus* as specific P(3HB-*co*-3HHx) degraders (Volova et al. 2017).

#### *Mcl*-PHA copolyester in soil

Lim et al. (2005) tested degradation of *mcl*-PHA produced by *Pseudomonas putida* PGA1 from saponified palm kernel oil in three different types of Malaysian soil after 112 days: acidic forest soil well-shaded under fallen decomposing leaves (FS), unshaded alkaline forest soil along a freshwater stream (FSst), and brackish mangrove soil (MS). Polymer samples (thin solvent-cast films of P(6.9-mol-%-3HHx-*co*-58.4-mol-%-3HO-*co*-26.7-mol-%-3HD-*co*-6.5-mol-%-3HDD-*co*-1.0-mol-%-3HTD-*co*-0.5-mol-%-3HHxD) were buried and encased in nylon nets at a depth of 2 cm. Degradation was determined via mass loss and amounted to 16.7% for samples buried in FS, 3.0% in FSst, and 4.5% in MS. Due to the fact that film samples did not show major change in molecular mass, and no change at all for melting temperature, glass transition temperature, and monomer composition, authors assumed that biodegradation took place at the polymer surface, not in the bulk. Via SEM, holes and cracks were easily visible, indicating the biodeterioration process taking place. P(3HB) and PE samples were used as positive and negative controls in all three sites. P(3HB) disintegrated completely in MS, and by 73.5% in FSst, but only by 4.6% in FS, demonstrating the significance of water movement during biodegradation of biopolymers. On the other hand, PE films did not show any mass loss at any test site. Monomeric composition of the *mcl*-PHA samples did not vary significantly during degradation form the original composition of the starting material, which indicated that depolymerases were not able to enter the polymer bulk under the given degradation conditions in soil, and degradation occurred predominately at the polymer surface (Lim et al. 2005).

Soil biodegradation of the *mcl*-PHA homopolyester poly(3-hydroxyoctanoate) (P3HO) produced by the company Bioplastech and its blends with PLA, the *scl*-PHA P(3HB), and PCL was tested by Narancic et al. (2018) according to ISO 17556. P(3HO) is an elastomeric material with expected medical applications (Bagdadi et al. 2018; Malagurski et al. 2021). The material is described as less prone to hydrolytic degradation than other types of PHA (Mallardé et al. 1998). Pristine P(3HO) did not reach more than about 6% biodegradation after 2 years, which demonstrates the high recalcitrance of this *mcl*-PHA towards biodegradation in comparison to the top-selling representatives of the PHA family. For PLA/P(3HO) (85/15) blends, no significant biodegradation was observed after the same time (pristine PLA did not biodegrade at all in soil under these test conditions). The blend of P(3HO)/P(3HB) (15/85) achieved the benchmark for “biodegradability” in the soil test environment, hence, 90% relative biodegradation within two years, the same was the case for blends of P(3HO) and PCL (15/85), which was also the case for pristine P(3HB) and PCL.

### Fresh water studies

#### P(3HB) homopolyester in fresh water

An early study conducted in 1995 by Mergaert et al. tested P(3HB) homopolyester biodegradation in natural freshwater ponds. Authors monitored mass loss, and changes in molecular mass and tensile strength of the materials. After 6 months, P(3HB) achieved 7% mass loss in a freshwater canal; after almost one year of submersion 34% mass loss was observed for this crystalline material (Mergaert et al. 1995).

Biodegradation of P(3HB) (manufacturer: Goodfellow) in freshwater (rainwater collected from ponds at University of Bayreuth, Germany) during a one year period was studied by Bagheri et al*.* (2017) and compared to the biodegradation of other “biopolyesters”: poly(lactic-*co*-glycolic acid) (PLGA), poly(ε-caprolactone) (PCL), polylactic acid (PLA), and Ecoflex (petrochemical polyester by BASF SE, consisting of the 1,4-butandiol, adipic acid and terephthalic acid monomers). The petrochemical polyester PET was also subjected to the same degradation study. All polyesters were processed into films by compression molding granules above their melt temperatures of granulates and tested in vials, which had openings enabling gas exchange. Vials were artificially illuminated by 16h/8h light/dark cycles to mimic day and night, and incubated at a constant temperature of 25 °C. The medium was refreshed at two-week intervals. After one year, P(3HB) degradation, expressed as mass loss, amounted to about 8.5%. This degradation during the year occurred as a linear function, and extrapolating the trend in the data showed that complete degradation might be achieved after about one decade. As expected, no biodegradation as mass loss was observed for the fossil PET; in addition. It was remarkable to see that fossil-based Ecoflex lost only roughly 1% of mass under the prevailing freshwater conditions, and PLA revealed no significant mass loss at all. P(3HB) degradation over one year also outperformed PCL, which lost only about 1% during this period. Among tested polyesters, only PLGA surmounted the biodegradation performance of P(3HB); PGLA was completely degraded after about 270 days, with rapid mass loss starting after about 4 months. Authors explained the slower degradation of P(3HB) relative to PGLA by the fact that in the highly crystalline P(3HB) degradation occurred via surface erosion only whereas the inner regions (bulk) of the P(3HB) was not reached easily by microbes and their enzymes during the test period, while the highly amorphous PGLA underwent water diffusion into the bulk of the sample, thus accelerating chain scission. These findings were substantiated by scanning electron microscope (SEM) and molecular mass determination, with the later confirming a constant molecular mass in P(3HB) sample through the year, while PGLA molecular mass dramatically decreased during degradation due to chain scission in the bulk of the sample. It is important to emphasize that these conditions operated with a low-density microbial community, in comparison to conditions prevailing in other natural freshwater bodies, such as lakes and ponds, which explained the modest degradation rates obtained (Bagheri et al. 2017). In general, PHA biodegradation is typically slower in fresh water than in marine environments due to the different concentrations of microorganisms, which was also substantiated later by Komiyama et al. (2021), who made direct comparison of PHA biodegradability in fresh and marine waters.

Vigneswari et al. studied biodegradation of solvent-cast P(3HB) films (Sigma Aldrich; 1.2 × 1.2 cm, strongly varying thickness from 0.3 to 6 mm, average mass 0.02 g) in a lake near the School of University Sains, Malaysia. Samples were placed in meshes. After 5 weeks, about 43% of the P(3HB) mass was shown to have degraded (Vigneswari et al. 2019).

Quinn et al. (2023) repeated their biodegradation studies for microbial P(3HB), synthetic P(3HB) and “syndio-rich P(3HB)” in soil (*vide supra*) in fresh water (25 °C, pH-value 7.2; 7.2 mg activated sludge was mixed with 192.8 mL mineral salt solution and 16.9 mg PHA samples). Glucose was used as positive control. Following the ISO14851 standard, microbially produced isotactic P(3HB) biodegradation in fresh water reached 50% within 90 days, which was in the same range for “syndio-rich P(3HB)” and for synthetic (*R*)-P(3HB). Through calculations based on first-order kinetics, authors predicted that “syndio-rich P(3HB)” will reach 90% biodegradation in soil after 268 days, while synthetic (*R*)-P(3HB) will reach this value already after 383 days, and microbial P(3HB) after 282 days under the given conditions. Comparing the biodegradation studies both in soil and in fresh water, authors argue that all three types of tested P(3HB) (microbial, synthetic, and “syndio-rich”), if accidently leaked into the environment, will readily biodegrade within a reasonable period of time (Quinn et al. 2023).

#### P(3HB) blends in fresh water

Marine biodegradation of blends of P(3HB), PBS, and PCL were studied by Narancic et al. (2018) according to standard ISO 14851 at 21 °C. According to this standard, biodegradation was monitored via CO_2_ evolution. While pristine P(3HB) was completely degraded after the test period, blends with PCL (40%) and PBS (50%) were degraded by about 35% and 60%, respectively. Blends of P(3HB) and PLA were not degraded at all under these conditions, again underlining the high recalcitrance of PLA against biodegradation under natural conditions, while not blended P(3HB) was completely degraded.

#### P(3HB-*co*-3HV) copolyesters in fresh water

The study by Mergaert et al. (1995) described above also tested P(3HB-*co*-10%-3HV) and P(3HB-*co*-20%-3HV) copolyesters in fresh water. After 358 days in a freshwater canal, 77% mass loss was observed in the P(3HB-*co*-10%-3-HV) samples, while the less crystalline P(3HB-*co*-20%-3HV) samples had already completely disappeared during the same time, while [P(3HB)] exhibited 34% mass loss in the same freshwater biodegradation experiment (*vide supra*) (Mergaert et al. 1995).

Komiyama et al. (2021) studied biodegradation of P(3HB-*co*-12-mol-%-3HV) copolyester prepared by the Monsanto Co. in fresh water with the samples having various shapes and forms, namely powder, cast film (100 µm thickness), undrawn monofilament fibers (0.5 mm thickness), and fivefold-drawn fibers (0.2 mm thickness), with the fibers having been produced via melt spinning. All samples were used for biodegradability tests in 10 mg quantities in 200 mL water, which was phosphate-buffered. Biochemical oxygen demand (BOD), mass loss, and scanning electron microscopy were used to monitor biodegradation. Pulverized polymer showed the highest degradation rate due to its larger surface area, which facilitated the best biofilm formation on the polymer particles. Degradation tests on the same samples were also studied in pond water at the Hongo campus of University of Tokyo. Temperature in ponds fluctuated from 14 to 28 °C, the pH-value was around 6.5–7.0, and the number of microorganisms from 41,000 to 61,000 CFUs per mL. Experiments were carried out by immersing the samples at 25 °C for 28 days under continuous stirring. Biochemical Oxygen Demand (BOD) tests revealed biodegradability for powder after 28 days of about 20%, which was observed to be the same for films and monofilament fibers, while only 7–8% of fivefold-drawn fibers degraded during the same period according to BOD. Interestingly, the large surface area of the powder made it easy for microorganisms to attach to the polyester particles to form a biofilm; here, biodegradation started earlier than was observed for the other samples, where a lag phase of 1–2 weeks was visible. Mass loss carried out showed that the films degraded faster than and undrawn fibers and significantly faster than the fivefold-drawn fibers. Again, higher surface area was beneficial for faster biodegradation. Although the fivefold-drawn fibers had a higher surface area than undrawn fibers (50 and 40 mm^2^, respectively), biodegradation of less crystalline undrawn fibers was faster. More than 90% of the films were degraded based on mass loss after 28 days, while for undrawn and fivefold-drawn fibers, degraded with about 30 and 65% remaining respectively (Komiyama et al. 2021).

#### P(3HB-*co*-4HB) copolyesters in fresh water

Omura et al. (2021) studied marine biodegradability of P(3HB-*co*-4HB) fibers of high tensile strength (> 200 MPa) and elasticity (elongation at break of ∼200%), obtained via melt-spinning. The polymer was obtained from Mitsubishi Gas Chemical Co., Inc., Japan, and had a 4HB content of 16 mol-%. Biodegradation tests were carried out using freshwater from Sanshiro Pond located at the University of Tokyo in open containers during over a period of 28 days at 25 °C with continuous stirring to emulate realistic and dynamic conditions; mass loss of samples was tested in seven-day intervals. After 7 days, no mass loss was observed, which was explained by the authors by the insufficient time needed by microbes to build a biofilm. After 14 days, about 20% mass loss was observed, and about 40% mass loss was observed after 3 weeks. Thereafter, degradation rate increased, and after 28 days, all samples degraded completely (Omura et al. 2021).

Vigneswari et al. (2019) studied biodegradation of solvent-cast P(3HB-*co*-4HB) films (prepared at Universiti Sains Malaysia; 1.2 × 1.2 cm, strongly varying thickness from 0.3 to 6 mm, average mass 0.02 g) in a lake near the School of University Sains, Malaysia. 4HB contents amounted to 14, 47, and 87 mol-%. Samples were placed in meshes. After 5 weeks, about 45, 57, and 82% of the P(3HB-*co*-14-mol-%-4HB), P(3HB-*co*-47-mol-%-4HB), and P(3HB-*co*-87-mol-%-4HB) films, respectively, were degraded (Vigneswari et al. 2019).

#### P(3HB-*co*-3HHx) copolyesters in fresh water

The biodegradation of P(3HB-*co*-3HHx) films in freshwater samples was studied in Japan in 2017 at Utsunomiya University in collaboration with PHA producer Kaneka Corporation, Hyogo, Japan. Six types of polyester films approximately 100 μm thick and 1 × 1–5 cm in size were tested for biodegradability: The plastics films consisted of poly(lactic acid) (PLA), poly(butylene adipate-*co*-terephthalate) (PBAT), poly(butylene succinate) (PBS), poly(butylene succinate-*co*-butylene adipate) (PBSA), poly(ε-caprolactone) (PCL), and microbial P(3HB-*co*-3HHx). Films were prepared by T-die cast extrusion. Biofilms formed on film surfaces after incubation in fresh water mixed with 0.5 g L^–1^ NH_4_Cl as the N source and 0.1 g L^–1^ KH_2_PO_4_ as the P source at 30 °C for 2 weeks. After incubation for 2 weeks, biofilm formation occurred on the surfaces of most tested films except PLA, which did not show any surface changes. For P(3HB-*co*-3HHx) and PCL films, surfaces were entirely covered with biofilm. For P(3HB-*co*-3HHx) films, rugged surface and formation of holes were observed, demonstrating the P(3HB-*co*-3HHx)-degrading activity of the bacterial community being present in the biofilm. 16S rRNA gene sequencing of the microbial community showed the dominance of the genera *Acidovorax* and *Undibacterium* in most biofilms formed on P(3HB-*co*-3HHx) films. To determine P(3HB-*co*-3HHx)-degrading activity, R2A agar medium containing 1 g L^–1^ P(3HB-*co*-3HHx) powder (Kaneka Corporation, Hyogo, Japan) as the sole carbon source was prepared in 24-well microplates. Sections of biofilms were placed on the center of R2A-PHBH agar plates. After incubation at 30 °C for one week, clearing zones around the colonies occurred due to P(3HB-*co*-3HHx) degradation. A total of 28 strains showed a clearing zone on these R2A-P(3HB-*co*-3HHx) plates; among them, 25 strains were closely related to *Acidovorax*. Five isolated strains were cultivated for three days on R2A-PHBH agar plates and in R2A liquid medium containing P(3HB-*co*-3HHx) films. All three isolates generated clearing zones on R2A-P(3HB-*co*-3HHx) agar plates; in addition, the transparency of the biofilm-coated P(3HB-*co*-3HHx) films decreased, and cracks and rough surfaces were observed, demonstrating P(3HB-*co*-3HHx) biodegradation (Morohoshi et al. 2018).

#### *Mcl*-PHA copolyester in fresh water

Ho et al. (2002) studied *mcl*-PHA biodegradation in tropical river water using an adapted ASTM D5209-01 test method in 2002. In this method strain *Pseudomonas putida* PGA1, an organism having Class II PHA synthase activity, was cultivated on saponified palm kernel oil as the sole carbon source for production of *mcl*-PHA constituting a P(54-mol-%-3HO-*co*-23-mol-%-3HD-*co*-15-mol-%-3HDD-*co*-8-mol-%-3HTD) copolyester. *Mcl*-PHA samples (solvent-cast films) were immersed in water from the Kayu Ara River, Malaysia, and degradation was evaluated by monitoring mass loss, changes in film surface morphology, monomer content, and the infrared (IR) spectrum of the PHA film. Test conditions involved water sampling, aerating, settling and removal of debris, and overnight storage at 4 °C before enrichment with mineral salts. 15 × 15 mm *mcl*-PHA films (thickness not disclosed) were methanol-sterilized and placed in nylon net bags, which were then placed in a reactor aerated with CO_2_-depleted air and stirred continuously at 28 °C. The outlet gas was transferred into a Ba(OH)_2_ solution to trap CO_2_, which resulted in the precipitation of the generated BaCO_3_. The remaining Ba(OH)_2_ was measured to determine CO_2_ evolution in the reaction setup. Incubation was performed with sampling after 0, 6, 20, 41, 60, and 86 days. As negative control, an empty nylon net was incubated, while P(3HB) acted as the positive control. Two additional experiments were carried out to test *mcl*-PHA degradation in unstirred sterilized and non-sterilized river water. During the process, pH-value of the water and the microbial population were monitored. The major outcomes of the study can be summarized as follows: in the short-term experiment (28 days), 10.6% mass loss was observed in unstirred and sterilized water, indicating a certain degradation without microbial attack. However, it is not described how this mass loss happened mechanistically (disintegration into smaller particles only without mineralization is most likely when compared with other abiotic PHA degradation studies). A parallel setup in sterilized, non-stirred water showed an increase of mass after 28 days; however, this was due to attachment of a microbial biofilm, which was not removed before mass determination; under these conditions, pH-value significantly dropped from 7.47 to 4.33 due to formation of hydroxyalkanoic acids and their oligomers, while only to 6.40 for sterilized water. CO_2_ formation was not followed in these short-term setups. For the long-term setups (86 days), complete disintegration of both *mcl*-PHA and the control P(3HB) was observed at the end of the test, but particles were found in the aqueous phase when testing *mcl*-PHA; these particles were collected and quantified, showing a degradation of 71.3% for the *mcl*-PHA film, and 100% for the P(3HB) control film. Sample surfaces were observed microscopically (SEM) during the process, exhibiting a transition from a smooth surface (start) to a surface showing cracks and holes already after 6 days (biodeterioration phase takes place). After 60 days, surfaces appeared uneven, and films changed from transparent to opaque. This contrasts with the short-term experiment in sterilized water, where films stayed transparent and surfaces did not change microscopically. In non-sterilized water used for parallel short-term study, samples became opaque, with a rough surface after 28 days. Moreover, it was shown that stirring accelerated the degradation process by mechanically eroding the surface, warranting a more efficient oxygen supply, and by rapid removal of water-soluble products generated. Interestingly, the monomeric composition of the *mcl*-PHA changed during the degradation process in the long-term experiment: after 6 days, 3HO (C8) units disappeared, while 3HTD (C14) occurred in highest proportion; this trend persisted until the end of the process. Hence, it was assumed that monomers with small chains were more quickly removed than the longer units (Ho et al. 2002). This in turn also showed that even for this completely amorphous *mcl*-PHA, degradation not only occurs at the surface, like that observed in highly crystalline PHA [e.g., P(3HB)] (Doi et al. 1990), but also the polymer bulk is easily accessible for water and depolymerase enzymes. 133.76 mg of CO_2_ evolved in the setup with *mcl*-PHA film set-up after 70 days, which was significantly higher than 115.72 for the control setup without *mcl*-PHA. This was attributed to the CO_2_ released from other carbon sources present in river water (Ho et al. 2002).

Biodegradation tests in fresh water according to ISO 14851 with P(3HO) from the company Bioplastech (Dublin, Ireland) and its blends with PLA, P(3HB), and PCL were carried out by Narancic et al. (2018) at 21 °C. Pristine P(3HO) showed about 50% biodegradation in fresh water environment after 56 days [P(3HB): about 90% after the same time]. For PLA/P(3HO) (85/15) blends, no significant biodegradation was observed after 56 days, the same goes for pristine PLA, which did not degrade at all. The blend of P(3HO)/P(3HB) (15/85) degraded by about 70% over 56 days [pristine P(3HB): 90%]. Test with P(3HO)/PCL blends (15/85) showed no significant degradation after 56 days (pristine PCL: about 50%).

### Marine water studies

Plastic pollution of oceans is one of the major concerns of the current age. It is estimated that about 8 million tons of plastic waste is currently discharged into marine environments *per annum*, mainly via wind and by river currents. According to the Ellen MacArthur Foundation, the total mass of plastics swimming in the oceans is expected to surpass the total mass of marine fish by 2050 in case the environmental input of plastic continues at current rates (World economic forum, reviewed by Rudnik and Briassoulis 2011; Suzuki et al., 2021). Therefore, PHA has been touted as an appealing solution to reduce marine plastic pollution. Indeed, microorganisms producing extracellular PHA-degrading enzymes [e.g., P(3HB) depolymerase (EC 3.1.1.75)] are common in the marine microbiome. A meta-study by Dilkes-Hoffman et al. from 2019 collates data for marine PHA biodegradation, assuming that mean rate of PHA biodegradation amounts to 0.04–0.09 mg·PHA day^−1^·cm^−2^ surface area, indicating that a bottle made of PHA has a mean lifetime of 1.5–3.5 years, while a thin PHA film will exist only 0.1–0.2 years (45–60 days) in the marine environment (Dilkes-Hoffman et al. 2019). Hence, a range of studies focused on biodegradability of PHA in marine settings, as detailed in the next sections for different types of commercially interesting types of PHA.

#### P(3HB) homopolyester in marine water

Doi et al. reported for the first time in 1992 on the biodegradation of microbial PHA biopolyesters in a marine-environment. The authors monitored weight loss of PHA films, plates, and fibers in sea water and recognized that samples were degraded via surface erosion, which depended on sea water temperature. P(3HB) homopolyester produced by *C. necator* from butyric acid was processed to thin film (thickness 50–150 µm, 5 × 10 cm in size) by chloroform-based solvent casting. Biodegradation experiments were carried out at the Kanagawa Prefectural Fishery Experiment Station, Jogashima (Japan). Samples were placed in nylon nets put in a cage made of stainless-steel and incubated in an outdoor tank (10 × 10 m × 3 m in depth) in 1.5 m water depth. Fresh sea water was allowed to continuously flow through the tank. Mass loss and surface erosion was monitored for 1 year, with sea water temperature fluctuating between 13 and 26 °C. After three weeks, the surface erosion for P(3HB) films amounted to about 12 µm, when water temperature was (22 + 3 °C). Unfortunately, mass loss data for the setups for P(3HB) were not disclosed in the publication. Interestingly, the authors also carried out degradation studies with sterilized sea water, showing that no mass loss occurred after four weeks of incubation, underlining the need for the appropriate microbiome for PHA biodegradation (Doi et al. 1992a, b).

P(3HB) samples studied by Mergaert et al. (1993) in seawater in the Zeebrugge harbor (Belgium) displayed 31% mass loss within 270 days of submersion. Interestingly, no changes in molecular mass were observed in this study, which indicates that degradation occurs predominantly at the polymer surface (Mergaert et al. 1993).

The now withdrawn standardized method ASTM 6691 (“Standard Test Method for Determining Aerobic Biodegradation of Plastic Materials in the Marine Environment by a Defined Microbial Consortium or Natural Sea Water Inoculum”) was used to evaluate marine biodegradability of extruded Metabolix P(3HB) films with an inoculum consisting of 13 marine microbial species at 30 °C. The mineralization was monitored via respiratory measurements using glucose as reference material. Resulting films were highly biodegradable in the microbe-enriched marine environment according to biodegradation requirements postulated by ASTM 6691; a minimum mineralization of 70% was reached after 40 days and surpassed 80% after 100 days. Additional biodegradation experiments were carried out via static and dynamic incubation in seawater. Under static conditions, films 1.27 × 1.27 cm in size were incubated in natural seawater at 21 °C, with or without natural sea sediment as inoculum. Dynamic testing of biodegradation was accomplished in tanks with samples exposed to continuously flowing seawater with and without contact to a sediment surface to mimic the open sea conditions and seasonal effects like temperature (in tests: 12–22 °C), pH-value (here: 7.9–8.1) and natural variation of nutrients availability (ammonia: 0.10–0.41 mg/L, phosphate: 0.01–0.095 mg/L). Dynamic tests lasted for 90 days. Dynamic conditions constitute a more realistic marine environment and is a better mirror of the biodegradation process in real-life settings. The lower and fluctuating water temperatures and the limiting nutrient supply in the dynamic tests resulted in slower biodegradation than in the static experiments. A mass loss of about 90% and 20–30% was monitored after 18 days of static incubation with and without addition of sea sediment, respectively. While under dynamic conditions, it took 63 days to lose about 50% and 20% with and without sediment addition, respectively (Thellen et al. 2008).

In 2010, Volova and colleagues studied biodegradation of P(3HB) and P(3HB-*co*-3HV) in the form of thin films and as compacted pellets in tropical marine environments in the South China Sea (Nha Trang, Vietnam). Temperature of the sea water only slightly fluctuated between 27 and 30 °C, pH-value varying between 7.1 and 7.5, and salinity between 3.2 and 3.5 ppm. The polymers were incubated for 160 days. The residual weight of P(3HB) and P(3HB-*co*-3HV) films was determined to be 58% and 54%, respectively, demonstrating a slightly faster degradation of the P(3HB-*co*-3HV) films relative to P(3HB). Interestingly, the mass of polymer pellets did not change in the first 80 days of incubation in sea water. The pellets did lose mass between 80 and 160 days, measured to be about 40% after 180 days which can be attributed to them having smaller surface area and therefore, a smaller polymer/water interface area compared to the thin PHA films (Volova et al. 2010).

Bagheri et al.’s freshwater degradation studies for the polyesters P(3HB), PLA, PCL, Ecoflex, PGLA, and PET were repeated under conditions mimicking marine environments using artificial seawater (AB Reef Salt, Aqua Medic, Germany) from a coral reef aquarium. These tests were also carried out for one year and gave similar results to those reported above for fresh water. No degradation was observed for PET and PLA, slight mass loss of about 1% for the Ecoflex and PCL films, about 6% for P(3HB), while complete degradation for PGLA was observed after about 270 days (Bagheri et al. 2017).

Tsuji and Suzuyoshi (2002) compared degradation of P(3HB), PLA, and PCL films in natural dynamic and static sea water at Akabane Fishing Port, Japan. Degradation and mechanical properties were monitored. Thin films (50 µm thickness) of the materials were fixed in meshes and put in cages that were lowered into the sea water to a depth of about 1 m. During the 5-week test period the water temperature was measured to be between 19 and 26 °C. After 2 weeks, mass loss of about 60% was observed in the P(3HB) films; mass loss for PCL was significantly lower (about 30%), while no mass loss was monitored for PLA after 2 weeks. At 5 weeks, the mass of the P(3HB) and PCL remained constant, while sudden increase of PLA mass loss was shown. Comparison experiments were conducted under controlled static seawater conditions (Tsuji and Suzuyoshi 2002). These tests gave a linear degradation trend for P(3HB), like the results disclosed by Bagheri et al. 2017, the P(3HB) lost about 5% of its mass after 5 weeks. The PCL film lost about 10% of its weight after 5 weeks, while no weight loss at all was observed for the PLA films, which matches well the findings previously reported by Bagheri et al. (2017). The authors noticed that dynamic conditions caused accelerated degradation of all tested films compared with controlled static conditions and explained this as being caused by the mechanical stress and strain experienced by the samples in the dynamic regime. Interestingly, this contrasts with the above-reported findings by Thellen et al. (2008), who found lower degradation rates under dynamic conditions. However, considering the discrepancies of PLA degradation in this study (no mass loss under static conditions, sudden mass loss under dynamic conditions), it is probable that under dynamic conditions prevailing in this study, strong mechanical fragmentation of films into smaller pieces might have occurred, which caused PLA mass loss via disintegration and not associated with PLA biodegradation via mineralization (Tsuji and Suzuyoshi 2022).

A sophisticated study by Derippe et al. (2024) reported on the impact of the chemical structure of PHA, together with the specificity of a biofilm settling on PHA specimens, on PHA biodegradation under marine conditions. P(3HB) produced by *Halomonas* sp. SF2003 on glucose as the carbon source was solvent-cast into films 80 to 120 µm thick and having a surface area of 13.5 cm^2^. These films were incubated for one month in an aquarium containing continuously circulating seawater from the Northwestern Mediterranean Sea, with temperature ranging between 19 °C and 24 °C and constant salinity of 38.5 g/L. Generated biofilms were detached from the PHA films and used for subsequent biodegradation studies. Here, sterile solvent-casted P(3HB) disks of 6 mm^2^ diameter were incubated in media containing the detached microbial biofilm for two months. The biopolymer acted as the sole carbon source in these setups. Biodegradation was followed via consumption of dissolved oxygen, PE and cellulose acted as negative and positive control, respectively. After two months, oxygen consumption reached about 4.5 × 10^−3^ µmol(O_2_) per mm^2^ P(3HB) surface. For cellulose films, this value amounted to 2.1 × 10^−3^ µmol(O_2_) per mm^2^ surface; no oxygen consumption was measured for the negative control PE (Derippe et al. 2024).

Microbeads consisting of fossil-based polymers like PE are frequently added to personal care products like shower gels, toothpaste, or face washes to achieve a peeling and scrubbing effect, or to act as sunscreens in sun protection products (Zhou et al. 2023). In all these cases, microplastic beads are added by default to generate the desired technological effect, hence, they are typical primary microplastics. A considerable part thereof is not efficiently separated from wastewater in sewage treatment plants, thus contributing to microplastic pollution of the ocean. Here, replacement of PHA microbeads offers a sustainable alternative. Hyodo et al. (2024) recently prepared uniformly shaped spherical P(3HB) microbeads with diameters ranging from about 50 μm to 150 μm via a simple, scalable melt homogenization method in silicone oil. Marine degradation of P(3HB) microbeads (degree of crystallinity: 65%; manufacturer: ICI) was tested *in situ* at the seabed off Misaki port in the northern Pacific Ocean, Japan, in a depth of 757 m. Mass loss tests were carried out to determine biodegradation. After 5 months, already 45% of tested P(3HB) microbeads were degraded. Moreover, biodegradability tests were also carried out with P(3HB) microbeads in seawater collected at Tokyo Bay at a water temperature of 27 °C by mixing 5 L seawater with 1 kg soil. After 25 days only, 85% of P(3HB) was degraded according to the BOD test, which was even higher than the value obtained for cellulose used as positive reference (77%) (Hyodo et al. 2024).

During the same study, authors carried out short- and long-term biodegradation tests at five different deep-sea floor locations in Pacific Ocean: three bathyal sites [off Misaki Port (BMS, depth 757 m), off Hatsushima Island (BHT, depth 855 m), and Myojin Knoll (BMJ, depth 1292 m)], and two abyssal sites [Kuroshio Extension Observatory (AKR, depth 5503 m) and Minamitorishima Island (AMN, depth 5552 m)]. Authors studied mass loss, reduction in film thickness, and morphological changes during deep-sea floor testing, biofilm formation, microbial accumulation, and genes responsible for biopolymer degradation under sea conditions. In addition to P(3HB) (manufacturer ICI), PHA copolyesters, other “bioplastics” like PLA, PCL, PBSA, etc., cellulose and its derivates, and petroplastics (PE, PP, PS, and PET) were studied. P(3HB) was processed to injection molded and melt-pressed films (1 cm × 3 cm × 0.4 cm, and 4 cm × 4 cm × 300 μm, respectively). PLA and petroplastics did not degrade at all. It was shown that the rate of biodegradation for PHA samples decreased with water depth. Analysis of available metagenomic datasets indicated that microorganisms encoding “bioplastic”-degrading enzymes such as PHA depolymerases are indeed present in extreme deep-sea locations (Hyodo et al. 2024).

#### P(3HB) blends in marine water

Marine biodegradation of blends of P(3HB), PBS, and PCL in natural seawater stemming from the Belgian coast were studied by Narancic et al. (2018) according to standard ASTM D6691 at 30 °C. Biodegradation was monitored via CO_2_ evolution. While pristine P(3HB) was completely degraded after the test period, blends with PLA (80%), PCL (40%), and PBS (50%) were degraded by about 15%, 85% (56 days), and 80% (56 days), respectively.

#### P(3HB-*co*-3HV) copolyester in marine water

Parallel to the studies with P(3HB) films at the Kanagawa Prefectural Fishery Experiment Station, Jogashima (Japan) (*vide supra*), Doi et al. (1992a, b) also studied biodegradation of P(3HB-*co*-3HV) copolyesters produced by *C. necator* from butyric and valeric acid. Samples had 3HV contents of 4 to 64 mol-%. Samples with 3HV contents of 4, 21, and 61 mol-% were processed to solvent-cast films, melt-extruded plates (2050–2100 µm thickness) were prepared from samples with 9, 13, and 15 mol-%. From a sample with 14 mol-% 3HV, monofilament fibers (260 µm diameter) were produced via melt-spinning. Surface erosion after 3 weeks of incubation in sea water amounted to about 13, 22, and 15 µm for films consisting of P(3HB-*co*-4-mol-%-3HV), P(3HB-*co*-21-mol-%-3HV), and P(3HB-*co*-61-mol-%-3HV), respectively, at (22 + 3 °C). Melt-extruded plates showed surface erosion of 130, 140, and 100 µm for P(3HB-*co*-9-mol-%-3HV), P(3HB-*co*-13-mol-%-3HV), and, P(3HB-*co*-15-mol-%-3HV), respectively, after 17 weeks of incubation in sea water at 21–+ 6 °C. Mass loss was determined only for the monofilament fiber consisting of P(3HB-*co*-14-mol-%-3HV): Mass loss of 25% was observed after four weeks of incubation, and a loss of 65% was observed after eight weeks at 21.6 °C (Doi et al. 1992a, b).

In the same study, described above, where Mergaert et al. (1995) investigated P(3HB) degradation in sea water, P(3HB-*co*-10%-3HV) and P(3HB-*co*-20%-3HV) copolyesters were also tested in the same marine environment (Zeebrugge harbor). These two polymers lost 49–52% of mass within 270 days, which is higher than the value reported by them for P(3HB) (31%, *vide supra*). Like P(3HB), the degradation rates of these copolyesters were higher during the summer compared to that in colder conditions (Mergaert et al. 1995). Volova et al. (2017) also reported slower biodegradation rates and similar mass loss for the same copolyesters at lower temperatures after 160 days (Volova et al. 2017). The impact of the 3HV fraction on the biodegradation rate of P(3HB-*co*-3HV) in natural environments matches *in vitro* findings reported by Choi et al. (2023) when P(3HB-*co*-3HV) samples were exposed to artificial solutions of extracellular fungal PHA depolymerase. These authors also noted that with increasing 3HV fraction the copolyester’s crystallinity decreased, which in turn resulted in faster biodegradation (Choi et al. 2023). This agrees with findings by Numata et al. (2008) and Shang et al. (2012), who describe that hydrolytic enzymes (extracellular PHA depolymerases) prefer amorphous polymer surfaces; less crystalline polymers like copolyesters are attacked more easily because of their less ordered structure, which makes the polymer chains more accessible to the enzymatic reactions.

Parallel to above-described tests with P(3HB), Thellen et al. (2008) also tested P(3HB-*co*-3HV) biodegradation according to ASTM 6691 under static and dynamic incubation in seawater with and without addition of marine sediment. This test using a marine consortium of 13 species resulted in about 80% mineralization of all Metabolix P(3HB-*co*-3HV) samples (5, 8, and 12% 3HV) after 3 weeks, and almost complete mineralization after 100 days. Under static conditions, P(3HB-*co*-12%-3HV) films displayed almost complete mass loss after 49 days when marine sediment was added, and almost 90% after the same time without sediment addition. Under dynamic conditions, the mass loss with and without sediment addition amounted to about 35% and 25%, respectively, after 63 days, and to about 50% and 32% after 90 days of incubation (Thellen et al. 2008).

In addition to P(3HB-*co*-3HV) films, Komiyama et al. (2021) also studied the biodegradation of undrawn monofilament fibers (0.5 mm thickness) of P(3HB-*co*-12-mol-%-3HV) copolyester obtained from the Monsanto Co. in sea water; fibers were prepared by melt spinning. Biochemical oxygen demand (BOD), mass loss, and scanning electron microscopy (SEM) were used to monitor biodegradation. Sample degradation was studied in seawater taken from Tokyo Bay. Temperature of the sea water was 30 °C, the pH-value was around 7.7, and the number of microorganisms was 223,000 CFUs per mL. Experiments were carried out by immersion of samples at 25 °C for 28 days under continuous stirring. BOD tests revealed 25% biodegradation of the fibers after 28 days and 90% degradation of the films during the same time (Komiyama et al. 2021).

Derippe et al. (2024) also carried out biodegradation of P(3HB-*co*-3HV) under the same conditions as their study on P(3HB) (mentioned above) in marine conditions with a detached microbial biofilm consortium produced by *Halomonas* sp. SF2003. The P(3HB-*co*-3HV) solvent-cast films 80 to 120 µm thick and a size of 13.5 cm^2^ were incubated for one month in continuously circulating seawater under the same conditions as described in the study for P(3HB). The generated biofilms were then detached from the films. In subsequent biodegradation studies, P(3HB-*co*-3HV) disks of 6 mm^2^ diameter were incubated for 2 months in media containing the detached microbial biofilm. After 2 months, oxygen consumption reached about 4.4 × 10^−3^µmol(O_2_) and 3.4 × 10^−3^ µmol(O_2_) per mm^2^ P(3HB-*co*-3HV) surface area for the two discs having 6 and 11% 3HV content respectively, which was marginally lower than that reported for the P(3HB) homopolyester [4.5 × 10^−3^ µmol(O_2_)] discs, but higher than for cellulose (Derippe et al. 2024).

Deroiné et al. (2015) studied aging of P(3HB-*co*-8-mol-%-3HV) extruded films (200 × 120 mm × 200 µm, produced from ENMAT Y1000P, Tianan Biological Materials Co. Ltd., PR China) in natural seawater in Lorient harbor, France, for 180 days by monitoring degradation via mass loss, SEM, DSC, and molecular mass determination. The sea water temperature fluctuated between 11 and 20 °C. Films displayed mass loss of about 36%, together with significant surface erosion and reduction of film thickness. Over 120 days of immersion, mass loss increased slowly but progressively to about 11%. After the initial 120 days, mass loss increased exponentially. After 9 months of immersion in natural seawater, films were completely disintegrated. Further, biodegradation of P(3HB-*co*-3HV) was studied via respirometric CO_2_ release in three different marine environments: a solid inoculum with foreshore sand, a solid–liquid inoculum with sand and seawater and a liquid inoculum with seawater. Cellulose was used as positive control. In foreshore sand, the biodegradation of the cellulose reached 97% after 600 days of incubation (theoretical biodegradation degree of 100% after 690 days), and 80% for P(3HB-*co*-8-mol-%-3HV) (theoretical 100% after 1690 days). This confirms the biodegradability of both materials according to standard NF U52-001, which require 70% degradation after 600 days. In a solid/liquid medium containing foreshore sand and seawater, mimicking a typical natural environment, biodegradation occurred even faster than in the solid foreshore sand medium: 90% degradation of P(3HB-*co*-8-mol-%-3HV) films was reached after 210 days. Biodegradation tests carried out in seawater inoculum with two concentrations of biofilm (5 and 50%) showed that P(3HB-*co*-8-mol-%-3HV) in 5% bacterial inoculum reached 97% biodegradation after 200 days of incubation, and 90% after 300 days for the 50% inoculum (Deroiné et al. 2015).

Volant et al. (2022) tested biodegradation of P(3HB-*co*-3-mol-%-3HV) microbeads with diameters ranging from 50 to 100 µm, also produced from ENMAT Y1000P PHA, by an emulsion-evaporation process. Biodegradability was studied according to the NF EN ISO 19679 test method that determined aerobic biodegradation of non-floating plastics at a seawater/sediment interface. Under continuous aeration in sealed systems, 5 g of microbeads were mixed with seawater (100 mL) and sediment (30 g), placed in a first test chamber, while the second chamber contained a NaOH absorbing solution and distilled water. A negative control without PHA was also tested in parallel as blank respiration of the seawater, while micronized cellulose acted as positive control. In addition, PLA microbeads were also tested in parallel setups. The experiment was carried out for 250 days at 25 °C. P(3HB-*co*-3HV) microbeads after 250 days of immersion reached 90% biodegradation, significantly higher than the PLA microbeads which reached about 22% degradation during the same period and even outperformed micronized cellulose, which reached about 75% degradation after 250 days (Volant et al. 2022).

In parallel to the tests for marine biodegradation of P(3HB) microbeads described above, Hyodo et al. (2024) also tested P(3HB-*co*-8-mol-%-3HV) microbeads (degree of crystallinity: 64%; manufacturer ICI) prepared analogously to the P(3HB) microbeads. In this case, biodegradability assessed via mass loss determination amounted to 44% after 5 months. Biodegradation tests based on BOD determination for P(3HB-*co*-8-mol-%-3HV) microbeads amounted to 74% after 25 days (Hyodo et al. 2024).

#### P(3HB-*co*-4HB) copolyester in marine water

In parallel to the studies with P(3HB) films and P(3HB-*co*-3HV) films, plates, and monofilament fibers at the Kanagawa Prefectural Fishery Experiment Station, Jogashima (Japan) (*vide supra*), Doi et al. (1992a, b) also studied biodegradation of P(3HB-*co*-4HB) copolyesters produced by *C. necator* from butyric acid and *γ*-butyrolactone (GBL). Samples containing 6 and 10 mol-% 4HB were studied. Both samples were processed to solvent-cast films (50–150 µm thickness). After eight weeks, films showed surface erosion of 31 and 33 µm for P(3HB-*co*-6-mol-%-4HB) and P(3HB-*co*-10-mol-%-4HB), respectively at a temperature of 14 °C, while in the warmer season (24 °C), surface erosion amounted to 55 and 60 µm, respectively, after eight weeks of incubation (Doi et al. 1992a, b).

Omura et al. (2024) studied marine biodegradability of P(3HB-*co*-4HB) fibers with very high tensile strength (> 200 MPa) and elasticity (elongation at break of ∼200%), obtained via melt-spinning. The polymer was supplied by Mitsubishi Gas Chemical Co., Inc., Japan., and had a 16 mol-% 4HB content. Biodegradation tests were carried out using seawater from Tokyo Bay in open containers for 28 days at 25 °C with continuous stirring to emulate realistic dynamic conditions; mass of the samples was measured in seven-day intervals. After 28 days, samples were completely degraded. After only seven days, 65% mass loss was observed; about 85% after 14 days, and about 95% after 21 days. This rapid degradation in comparison to freshwater experiments with samples produced in an identical way (*vide supra*) was explained by the authors by the five-fold higher number of microorganisms present in the studied marine environment than in the fresh water (Omura et al. 2024).

In addition to P(3HB) and P(3HB-*co*-8-mol-%-3HV) microbeads (*vide supra*), Hyodo et al. (2024) also tested P(3HB-*co*-8.9-mol-%-4HB) microbeads (degree of crystallinity: 50%; manufacturer: Mirel™) prepared analogously to the P(3HB) and P(3HB-*co*-8-mol-%-3HV) microbeads. In this case, biodegradability assessed via mass loss determination amounted to 52% after 5 months. BOD determination of biodegradation revealed a degree of degradation of 83% after 25 days of immersion in sea water (Hyodo et al. 2024).

#### P(3HB-*co*-3HHx) copolyester in marine water

The emerging hybrid *scl*-*mcl*-PHA copolyester P(3HB-*co*-3HHx) is becoming a popular choice for commercial production and use with several producers such as Kaneka, Danimer Scientific, RWDC, and BluePha (Koller and Mukherjee 2022) introducing them in the market in the last several years. The biodegradability of P(3HB-*co*-3HHx) in marine setting was established by a study by Sashiwa et al. (2018), who tested P(3HB-*co*-11-mol-%-3HHx) (X151A, KANEKA^©^ Biodegradable polymer™ PHBH, Kaneka Co., Osaka, Japan) and its blends with PLA (Ingeo 10361D, Nature Works, USA), PBAT (Ecoflex C-1200, BASF SE), or PBS (Bionolle 1020MD, Showa Denko, Japan) in seawater during a 28 days period. Pristine P(3HB-*co*-3HHx) degraded to 51% of its mass (depending on particle size) when tested as powder. This was higher than the degradation of the blends that were also tested in parallel. Blends were prepared via melt extrusion; resulting pellets were freeze pulverized into powder for and used for the biodegradation studies. Biodegradation was assessed via measuring microbial O_2_ consumption. P(3HB-*co*-3HHx) blends with PBAT, PLA, or PBS showed lower biodegradation after 28 days with decreasing P(3HB-*co*-3HHx) content. Pure PBAT, PBS and PLA exhibited biodegradation by about 1% after 28 days which is well within the experimental error of the tests (Sashiwa et al. 2018).

Under aerobic seawater conditions, canola oil-derived Nodax poly(3HB-*co*-7.1%-3HHx) sheets and poly(3HB-*co*-6.5%-3HHx) flakes from DaniMer Scientific were incubated for 148–195 days at room temperature in seawater taken from the coast of Georgia, USA. Degradation experiments were done with continuous shaking mimicking dynamic sea conditions. Up to 83% of gaseous carbon loss were monitored (as CO_2_ evolution) after 6 months for poly(3HB-*co*-6.5%-3HHx) flakes, which was like that shown for cellulose, used as a positive control. In contrast to poly(3HB-*co*-6.5%-3HHx) flakes, degradation of poly(3HB-*co*-7.1%-3HHx) sheets had a high variance, probably due to a highly diverse microbial activity on the samples’ surfaces (Wang et al. 2018).

Parallel to the tests on P(3HB-*co*-3-mol-%-3HV) microbeads using the standard NF EN ISO 19679, Volant et al. (2022) tested biodegradation of microbeads produced from Aonilex X131A P(3HB-*co*-6-mol-%-3HHx) and Aonilex X151A (P(3HB-*co*-11-mol-%-3HHx), from Kaneka Corporation (Japan). Microbeads (50 to 100 µm diameter) of these materials also were obtained by an emulsion-evaporation process, and biodegradation was studied following the standard NF EN ISO 19679 for 250 days at 25 °C. Aonilex X131A P(3HB-*co*-6-mol-%-3HHx) and Aonilex X151A (P(3HB-*co*-11-mol-%-3HHx) microbeads reached 62% and 80% biodegradation respectively, lower than that reported for the P(3HB-*co*-3-mol-%-3HV) microbeads, but is markedly higher than the PLA microbeads (about 22% biodegradation) also tested in parallel, and similar to the micronized cellulose (about 75% degradation after 250 days) used as a positive control. This study underlines the high impact the chemical composition of PHA has on the biodegradation rate under the same test conditions (Volant et al. 2022).

P(3HB-*co*-6-mol-%3HHx) microbeads were tested as the fourth group of PHA biopolyesters in the deep-water test series of Hyodo et al. (2024). The polymer was obtained from Kaneka, Japan. Here, deep sea degradation under extreme conditions in a depth of 757 m resulted in a mass loss of 20% after 5 months. Immersion in sea water at 27 °C, monitored via BOD determination, showed a degree of degradation of 68 °C (Hyodo et al. 2024).

During the same study, authors carried out short- and long-term biodegradation tests (*vide supra*) for P(3HB-*co*-9-mol-%-3HHx) (Kaneka Corporation). Detailed photographic biodegradation after one year revealed that the surface morphology before degradation was very smooth, sample surfaces after placement on the shore or the deep-sea floor appeared uneven, indicating that degradation took place. It was shown that degradation proceeded from the surface homogeneously; this was evident from the strong decrease in thickness of samples from the shore (PJM12). In about 1 year, thickness of the samples decreased by ~ 700 μm at the shore (PJM12), ~ 110 μm off Hatsushima Island (BHT14), and ~ 10 μm at Minamitorishima Island (AMN13) (original thickness: 4000 µm). At the Hatsushima Island sampling site (BHT, depth 855 m), P(3HB-*co*-3HHx) film mass decreased by 22% in 3 months and 52% in 8 months. From initially 225 μm, film thickness decreased by 35 μm over 3 months and 70 μm during 8 months (Hyodo et al. 2024).

#### *Mcl*-PHA copolyester in marine water

Derippe et al. (2024) studied the biodegradability of *mcl*-PHA films in the same experiments conducted on P(3HB) and P(3HB-*co*-3HV), using detached biofilm generated on PHA films in marine water. *Pseudomonas putida* KT2440 was used as the *mcl*-PHA production strain; octanoic, heptanoic or nonanoic acid or a mix of nonanoic acid and acrylic acid were used to produce *mcl*-PHA samples of different compositions: samples included “PHO”, “PHN”, and “PHNac” were P(5.5%-3HHx-*co*-89%-3HO-*co*-5.5%-3HD), P(14%-3HHp-*co*-4%-3HO-*co*-58.1%-3HN-*co*-24%-3HD), and P(23%-3HHp-*co*-74%-3HN-*co*-2%-3HD). They showed that oxygen consumption during 2 months of incubation, representing PHA biodegradation, was lower than for tested *scl*-PHA samples compared to the *mcl*-PHA terpolyesters, and amounted to 0.18 × 10^−3^, 0.70 × 10^−3^, and 0.31 × 10^−3^ µmol(O_2_) per mm^2^ *mcl*-PHA film. This indicates that *mcl*-PHA was slowly but still noticeably mineralized under the given conditions, and that for individual *mcl*-PHA samples, the chemical composition is highly relevant for the biodegradation rate, while no oxygen consumption was observed for the fossil-based negative control PE (Derippe et al. 2024).

Biodegradation tests according to ASTM D6691 with P(3HO) from the company Bioplastech and its blends with PLA, P(3HB), and PCL were carried out by Narancic et al. (2018) at 30 °C. Pristine P(3HO) showed about 40% biodegradation in marine environment after 56 days (P(3HB): about 90% after the same time). For PLA/P(3HO) (85/15) blends, no significant biodegradation was observed after 56 days, while pristine PLA showed a biodegradation rate of only about 10% after this time. The blend of P(3HO)/P(3HB) (15/85) had a slower degradation rate (about 80%) during the first ten days compared to pristine P(3HB); after 56 days, however the blend reached almost the same level of biodegradation as pristine P(3HB) in marine test conditions. Test with P(3HO)/PCL blends (15/85) were degraded by about 65% after 56 days (pristine PCL: about 80%).

### P(3HB) blends under industrial composting conditions

Industrial composting of blends of P(3HB), PBS, and PCL under managed conditions according to ISO 14855 at 58 °C were studied by Narancic et al. (2018). Not blended pristine P(3HB) was completely composted after about 25 days only, the blend of P(3HB)/PCL (60/40) after only 20 days. P(3HB)/PBS (50/50) blends reached complete composting after about 60 days.

### P(3HB) blends under home composting conditions

Home composting of blends of P(3HB), PBS, and PCL under managed conditions according to ISO 14855 at 28 °C were mimicked by Narancic et al. (2018). Not blended pristine P(3HB) turned out to fulfill the home composting criteria, as well as the blend of P(3HB)/PCL (60/40) and the P(3HB)/PBS (50/50) blend. In contrast, the P(3HB)/PLA (20/80) blend did not show home compostability.

### Biodegradation in aerobic wastewater treatment plants

#### P(3HB-*co*-4HB) copolyester under aerobic activated sludge conditions

Doi et al. (1990) carried out biodegradation studies with solvent-cast P(3HB-*co*-10%-4HB) films with a thickness of 0.7 mm in an activated sludge from a sewage treatment plant at Tokyo Institute of Technology, Japan, under aeration at 30 °C. In comparison, biodegradation of P(3HB) samples was tested. PHA was produced by *C. necator* H16. Degradation of test specimens was monitored simply by observing the appearance after 2 and 5 weeks. P(3HB-*co*-10%-4HB) films were almost completely decomposed after 2 weeks, and completely disappeared after 5 weeks, which was faster than biodegradation of the P(3HB) test films. Unfortunately, no quantitative data were provided by the authors for the biodegradation rate (Doi et al. 1990).

#### P(3HB-*co*-3HHx) copolyester under aerobic wastewater treatment conditions

White et al. (2021) studied P(3HB-*co*-3HHx) samples from RWDC Industries (3HHx content not reported) exposed to an operational wastewater treatment plant (WWTP) in an aerobic aeration basin during 13 weeks via RAMAN microscopy, thermogravimetric analysis, and differential scanning calorimetry (DSC). In parallel, activated sludge from this WWTP was used as inoculum to study biodegradation under controlled respiratory laboratory experiments. P(3HB-*co*-3HHx) microbead samples were studied in an epoxy-resin bound form, as free microbeads, as films; for comparison, PLA films were also studied. A carbon mineralization of 90, 89, 95, and 8% was achieved for epoxy-resin bound P(3HB-*co*-3HHx) microbeads, free P(3HB-*co*-3HHx) microbeads, P(3HB-*co*-3HHx) films, and PLA films, respectively. These calculations were made based on the 100% mineralized positive reference cellulose. Calculating the biological degradation rate for tested materials resulted in 32-, 30-, 18-, and 19-mL CO_2_·g^−1^·day^−1^ for cellulose, P(3HB-*co*-3HHx) films, free P(3HB-*co*-3HHx) microbeads, and epoxy-resin bound P(3HB-*co*-3HHx) microbeads, respectively (White et al. 2021).

### P(3HB) homopolyester and P(3HB-*co*-3HHx) copolyester under activated sludge conditions

P(3HB-*co*-12%-3HHx) copolyester films (manufacturer: Shantou Lianyi Biotech Company, Guangdong, PR China; produced via solvent casting, 100 µm thickness) were subjected to immersion in reactors filled with nutrient-depleted activated sludge (origin not disclosed by the authors) at room temperature. After 18 days, 40% of the films were degraded according to mass loss determination, which is considerably higher than values obtained for analogously obtained P(3HB) films (20% mass loss after 18 days) and Ecoflex-films from BASF SE (5% mass loss after 18 days) (Morse et al. 2011).

### Anaerobic biodegradation studies

#### P(3HB) under anaerobic conditions

Narancic et al. studied aqueous anaerobic degradation of P(3HB) and its blends according to ISO 14853. Activated sludge was suspended in an oxygen-free medium at a constant temperature of 35 °C; CO_2_ and CH_4_ evolution was measured to quantify degradation. Under these conditions, the non-blended P(3HB) was completely degraded, as well as the blends of P(3HB) and PLA (20/80) and P(3HB) and PCL (60/40). Degradation of the blend P(3HB) and PBS (50/50) under these conditions amounted to almost 80%.

#### P(3HB-*co*-3HHx) copolyester under anaerobic conditions

Morse and colleagues carried out detailed studies to explore the effects of monomeric P(3HB-*co*-3HHx) copolyester composition (3HHx fraction), degree of crystallinity, and morphology on anaerobic biodegradation of P(3HB-*co*-3HHx) films, containing molar 3HHx fractions of 3.8–10% at random distribution. Films of 0.3 mm thickness were prepared from Procter & Gamble’s P(3HB-*co*-3HHx) powder by hydraulic hot pressing. Anaerobic biodegradation was studied in an environment containing anaerobic biosolids from an anaerobic digester at a wastewater treatment plant at a constant temperature of 37 °C. Biogas production was monitored during the process for twelve days, and samples were monitored for mass loss. Authors observed that increased 3HHx content correlated with decreased crystallinity and increased degradation rate. While samples of poly(3HB-*co*-10%-3HHx) lost 80% of its original mass after seven days of incubation, mass loss for poly(3HB-*co*-3.8%-3HHx) tested amounted to only 28%. After 10 days, the poly(3HB-*co*-10%-3HHx) samples had completely disappeared, and the poly(3HB-*co*-3.8%-3HHx) had completely biodegraded after twelve days. SEM micrographs indicated that  degradation proceeds at the polymer surface, and preferred amorphous regions, exposing crystalline spherulites inside the sample which degraded after the amorphous regions. Importantly, the higher the 3HHx fraction samples contained smaller the crystalline spherulites which favored rapid biodegradation. Finally, it was also shown by the authors that annealing at 70 °C strongly enhances biodegradability by forming voids in the sample’s semi-crystalline structure, which facilitate penetration of water and enzymes into the polymer structure (Morse et al. 2011).

Wang et al. (2018) studied anaerobic biodegradation of P(3HB-*co*-3HHx) using an inoculum from an anaerobic digester of a wastewater treatment plant, containing sludge and some lipids. Tests were carried out under continuous shaking of digester vessels at 38 °C for 85 days. Biogas formation was regularly measured to monitor P(3HB-*co*-3HHx) biodegradation. After 40–60 days of incubation, about 55, 77, and 63% of the total carbon contained in P(3HB-*co*-6.5%-3HHx) flakes, P(3HB-*co*-7.1%-3HHx) sheets, and the positive control cellulose were converted to biogas. For the negative reference polypropene (PP), no carbon loss as biogas was monitored (Wang et al. 2018).

#### P(3HB-*co*-3HO) copolyester under anaerobic conditions

Anaerobic biodegradation of the hybrid *scl-mcl*-PHA P(3HB-*co*-10-mol%-3HO) was tested by Federle et al. (2002) and compared to anaerobic biodegradation of the fossil-based polyester PCL. P(3HB-*co*-10-mol%-3HO) was radio-labeled and chemically synthesized, and incubated with samples of anaerobic digester sludge, septage, freshwater sediment, and marine sediment under conditions similar to those occurring *in situ*. In addition, laboratory-scale landfill reactors were used for incubation experiments. For comparison, radio-labeled PCL was investigated during incubation in anaerobic digester sludge and in landfill reactors. Biodegradation of both polyesters was monitored via evolution of ^14^CO_2_ and ^14^CH_4_. While P(3HB-*co*-10-mol%-3HO) was rapidly biodegraded (half-lives less than 30 days) in digester sludge, septage sediments, and landfill reactors, PCL had not significantly degraded in digester sludge over 122 days and showed slow degradation in landfill reactors. For example, in anaerobic digester sludge incubated at 35 °C, mineralization of P(3HB-*co*-10-mol%-3HO) amounted to about 90% after 60 days, which is much more than for lignocellulose (about 20%) and PCL (almost no gas evolution) tested in parallel. Similar degree of mineralization was observed for P(3HB-*co*-10-mol%-3HO) in septage, marine sediment, and freshwater sediment. In all setups, P(3HB-*co*-10-mol%-3HO) degradation was faster than for lignocellulose. This study indicates that even an “unusual” type of PHA, P(3HB-*co*-10-mol%-3HO), is readily mineralized under diverse anaerobic conditions, while biodegradation of PCL, often termed a “biodegradable plastic”, occurs at considerably lower rates (Federle et al. 2002).

## Conclusions

Environmental pollution from fossil plastics and plastic accumulation in every biome on this beautiful planet continues unabated. The only plan humanity has come up with so far is to landfill trash or incinerate it. Incineration (mostly in the EU) is putting massive additions of planet-warming CO_2_ into the atmosphere, while landfilling (everywhere else) creates an opportunity for microplastics to persist well past the year 10,000 C.E. It is not clear if *homo sapiens* have reached a point of no return where Earth will be forever contaminated, but the next decade will see more dramatic and negative consequences from fossil plastic use than in the last 75 years since their invention. There is a significant increase in scientific reports from physicians, microbiologists, medical scientists, and zoologists on the presence of microplastics in every imaginable body part of humans and animals examined. These reports are only starting to scratch the surface of the negative health effects imbued by microplastics for all forms of life. Within a sufficiently long timeframe, all plastics will break down. The broken-down plastics are creating micro- and nanoplastics that then exude additives and chemicals that embody myriad threats to the eco- and biosphere.

PHA biopolyesters have firmly established themselves as possessing attributes and functionality to be truly sustainable and circular materials that conform to the principles of green chemistry. This perfectly aligns with the current endeavors of the European Green Deal, and the UN’s effort on reducing plastics pollution [The Legally Binding Treaty of Plastics, online resource 17, or The 3rd UN Ocean Conference (UNOC3, 9–13 June 2025, Nice, online resource 18)], empowering the ocean conomy all of which explicitly postulates that shifting towards a more sustainable society and towards a Circular Economy is closely intertwined with the way of production, use, and disposal of plastic materials in the European economy (Di Bartolo et al. 2021) and worldwide. PHAs are currently witnessing a new wave of industrialization and are growing in visibility among brand owners and converters (Koller and Mukherjee 2022). Policy makers are only beginning to understand the magnitude of the plastics pollution problem and it is difficult to grasp the negative consequences of plastic pollution on animals and humans, or the need to find substitutes like PHA biopolymers. Given the growing number of relevant studies on PHA’s attributes and functionalities, especially their diverse end of life profile (recycle, reuse, biodegrade, compost), there is an urgent need for active communication with all stakeholders to make PHA a primary target for substitution of fossil plastics. Proliferating PHA use will contribute to meeting the ambitious 2050 net-zero emissions goals set by the European Union, and similar endeavors worldwide (Di Bartolo et al. 2021).

This review attempts to highlight in clear terms with appropriate references to authoritative studies that the biodegradability of PHA biopolymers under diverse and realistic natural and industrial conditions have been demonstrated beyond a reasonable doubt.

It has been shown that all types of natural PHA biopolyesters examined to date are biodegradable in all studies, including often in challenging environments, be it in soil, fresh- or sea water, in activated sludge and under anaerobic conditions. For all major types of commercially available, industrialized PHA which can be used to replace 50% of consumer plastics, these projections are iron-clad. It is obvious that environmental conditions affect PHA degradation; e.g., in tropical sea waters PHA will biodegrade faster than in the Northern hemisphere, and the composition and humidity of soil strongly affects PHA degradation rates. In addition, the structure of a given PHA impacts its morphology, including crystallinity, and is also decisive in their biodegradation rates. This dependence of biodegradability on environmental conditions is also true for all organic matter including cellulose and other biopolymers.

Despite strong background studies, the bulk of data to date use the residual mass as the key parameter to evaluate biodegradability of PHA. Mass loss is also influenced by other abiotic parameters, such as water currents, UV irradiation, thermal and mechanical stresses. These parameters cause disintegration and fragmentation of all materials including PHA, but this disintegration and fragmentation is distinct from biodegradation, which is defined as the complete mineralization of a material, generally a biomaterial such as PHA, by a microorganism. Biological degradation, defined in widely used standards like ASTM D6400, ISO 14855, and EN 13432:2000, is defined based on the amount of CO_2_ released during the mineralization process. Despite the enormous body of studies describing the biodegradation of emerging types of PHA, such as P(3HB), P(4HB), their copolymers and various *mcl*-PHA, under optimized conditions using isolated highly concentrated enzyme formations, there is a significant lack of data for their biodegradation under real life environmental conditions. Here, the authors of the current review note an urgent need for additional studies to provide a holistic picture of environmental PHA biodegradation.

In a nod to practical science, these studies should focus on the biodegradation of commercial PHA based products, be it bottles, cutlery, dishes, bags, etc., made by various manufacturers from different types of PHA, under realistic environmental conditions. The mere use of thin PHA films or microbeads, as is the case in many studies, highlighted in this review, does not mirror the realistic situation of bioplastic items disposed in the environment, be it littered accidently or due to a lack of appropriate industrial waste management system. Here, biodegradability performance needs to be benchmarked against values obtained from standardized tests, where specimens are typically subjected to biodegradation tests in an ideal shape and size in an optimum environment. The authors foresee that such tests would merely confirm what is already known and described in this review: that PHA biopolymers are biodegradable in soil, fresh- and sea water and in anerobic conditions. However, this demonstration of the biodegradation of PHA-based articles and products is necessary and essential to provide fuel for brand owners, policy makers and the consumer to switch to PHA biopolymers.

## Data Availability

No datasets were generated or analysed during the current study.
